# Subcutaneous white adipose tissue–derived extracellular vesicles maintain intestinal homeostasis via IgA biosynthesis in aging mice

**DOI:** 10.1172/JCI188947

**Published:** 2025-11-17

**Authors:** KeKao Long, Pujie Liu, Yi Wang, Jordy Evan Sulaiman, Moinul Hoque, Gloria Hoi Yee Li, Daisy Danyue Zhao, Pui-Kei Lee, Gilman Kit-hang Siu, Annie Wing-tung Lee, Zhuohao Liu, Pui-kin So, Yin Cai, Connie Wai-hong Woo, Chi-bun Chan, Aimin Xu, Kenneth King-yip Cheng

**Affiliations:** 1 Department of Health Technology and Informatics,; 2 Shenzhen Research Institute, and; 3 Department of Food Science and Nutrition, The Hong Kong Polytechnic University, Hong Kong, China.; 4 Department of Neurosurgery, Shenzhen Hospital, Southern Medical University, Shenzhen, Guangdong, China.; 5 University Research Facility in Life Sciences, The Hong Kong Polytechnic University, Hong Kong, China.; 6 The State Key Laboratory of Pharmaceutical Biotechnology,; 7 Department of Pharmacology and Pharmacy,; 8 School of Biological Sciences,; 9 Guangdong–Hong Kong Joint Laboratory for Metabolic Medicine, and; 10 Department of Medicine, The University of Hong Kong, Hong Kong, China.

**Keywords:** Aging, Endocrinology, Immunology, Adipose tissue, B cells, Immunoglobulins

## Abstract

Intestinal function and white adipose tissue (WAT) function deteriorate with age, but whether and how their deterioration is intertwined remains unknown. Increased gut permeability, microbiota dysbiosis, and aberrant immune microenvironment are the hallmarks of intestinal dysfunctions in aging. Here, we show that subcutaneous WAT dysfunction triggered aging-like intestinal dysfunctions in mouse models. Removal of inguinal subcutaneous WAT (iWAT) increased intestinal permeability and inflammation and altered gut microbiota composition as well as susceptibility to pathogen infection in mouse models. These intestinal dysfunctions were accompanied by a reduction of immunoglobulin A–producing (IgA-producing) cells and IgA biosynthesis in the lamina propria of the small intestine. Retinoic acid (RA) is a key cargo within iWAT-derived extracellular vesicles (iWAT-EVs), which, at least in part, elicits IgA class-switching and production in the small intestine and maintains microbiota homeostasis. RA content in iWAT-EVs and intestinal IgA biosynthesis are reduced during aging in mice. Replenishment of “young” iWAT-EVs rejuvenates intestinal IgA production machinery and shifts microbiota composition of aged mice to a “youth” status, which alleviates leaky gut via RA. In conclusion, our findings suggest that iWAT-EVs with RA orchestrate IgA-mediated gut microbiota homeostasis by acting on intestinal B cells, thereby maintaining intestinal health during aging.

## Abstract 2

iWAT-EVs with RA orchestrate IgA-mediated gut microbiota homeostasis by acting on intestinal B cells, thereby maintaining intestinal health during aging.

## Graphical abstract

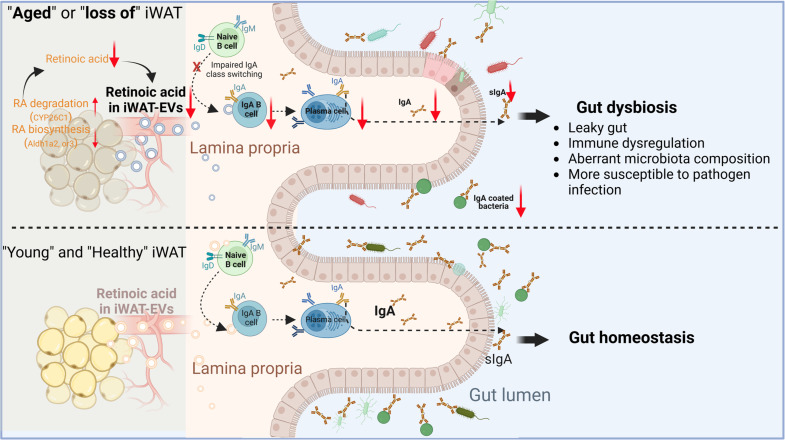

## Introduction

White adipose tissue (WAT) maintains systemic metabolic and immune homeostasis. WAT at different anatomical sites exhibits distinct properties, secretome, and functions. Generally, excessive visceral WAT (vWAT; surrounding the internal organs) is associated with increased risks of metabolic diseases, whereas expansion of subcutaneous WAT (sWAT; under the skin) is thought to be metabolically protective (1). White adipocytes within WAT exert their metabolic and immunological actions by secreting numerous bioactive factors, collectively adipokines (2, 3). These adipokines act as endocrine messengers to mediate crosstalk between WAT and distal metabolic tissues. For instance, adiponectin promotes insulin sensitivity and fatty acid oxidation in the liver and skeletal muscle and has antiinflammatory effects on the blood vessels (4). Apart from the protein factors, new classes of adipokines, such as extracellular vesicles (EVs), RNA, and small metabolites, have been identified (5–8), yet their physiological relevance remains poorly characterized.

The intestinal tract harbors over a trillion commensal microorganisms that regulate almost every aspect of the host’s health. Gut microbiota homeostasis is tightly controlled by interaction with host intestinal immunity and nutritional factors. Importantly, the interaction changes with age and metabolic status, which in turn regulates intestinal functions and health (9). Immunoglobulin A (IgA) is the most abundant antibody in the intestine, produced by the IgA plasma cells, and is required to maintain intestinal homeostasis and microbiota composition, immunity, energy balance, and glucose homeostasis (10–15). The generation of intestinal IgA-producing cells requires interactions among host immune cells, i.e., B cells, T cells, and dendritic cells, as well as gut microbiota and dietary factors (16–18). In aging, intestinal B cell senescence leads to reduced IgA abundance and diversity, thereby altering microbiota composition (19). However, whether extra-intestinal factors, such as adipokines, affect intestinal IgA production and functions during aging has not been explored.

The regulatory actions of adipose tissues on intestinal functions have been recently reported. For instance, disruption of the endocannabinoid system in adipocytes induces obesity and glucose intolerance, partly through alterations in the gut microbiota (20). Adiponectin has been shown to protect against acute colitis by inhibiting immune cell infiltration in the large intestine of mice (21). Wei et al. demonstrated that vWAT controls intestinal inflammation by modulating M1 macrophage polarization through the secretion of exosomes containing microRNA-155 in obesity-related Crohn’s disease (6). Additionally, Zhang et al. found that adipocytes with reduced iron uptake prevent diet-induced obesity by decreasing lipid absorption in the intestine via an unknown adipokine (22). Notably, most previous studies have focused on the relationship between vWAT and the intestinal system in the context of obesity and Crohn’s disease, but not in aging.

Multiomics studies indicate that WAT is the first tissue to show functional decline in aging (23, 24). Proinflammatory secretome is commonly observed in elderly humans and aged rodents. Concurrently, intestinal dysfunction including disruption of microbiota balance, reduced IgA production, and increased gut permeability occurs with aging (19, 25). We thus hypothesize that WAT dysfunction might contribute to intestinal dysfunction in aging. Using surgical, genetically modified, and natural aging animal models, we herein demonstrate that inguinal sWAT (iWAT) secretes EVs containing retinoic acid, which selectively deliver to the small intestine, where IgA class switching and IgA production are promoted, thereby maintaining gut homeostasis. Such regulatory actions of iWAT on the intestinal immune system deteriorate during aging. Replenishing “young” iWAT-EVs is sufficient to restore IgA biosynthesis and microbiota balance in the natural aging mouse model.

## Results

### Loss of iWAT disrupts intestinal functions and gut microbiota composition.

**Figure 1:**
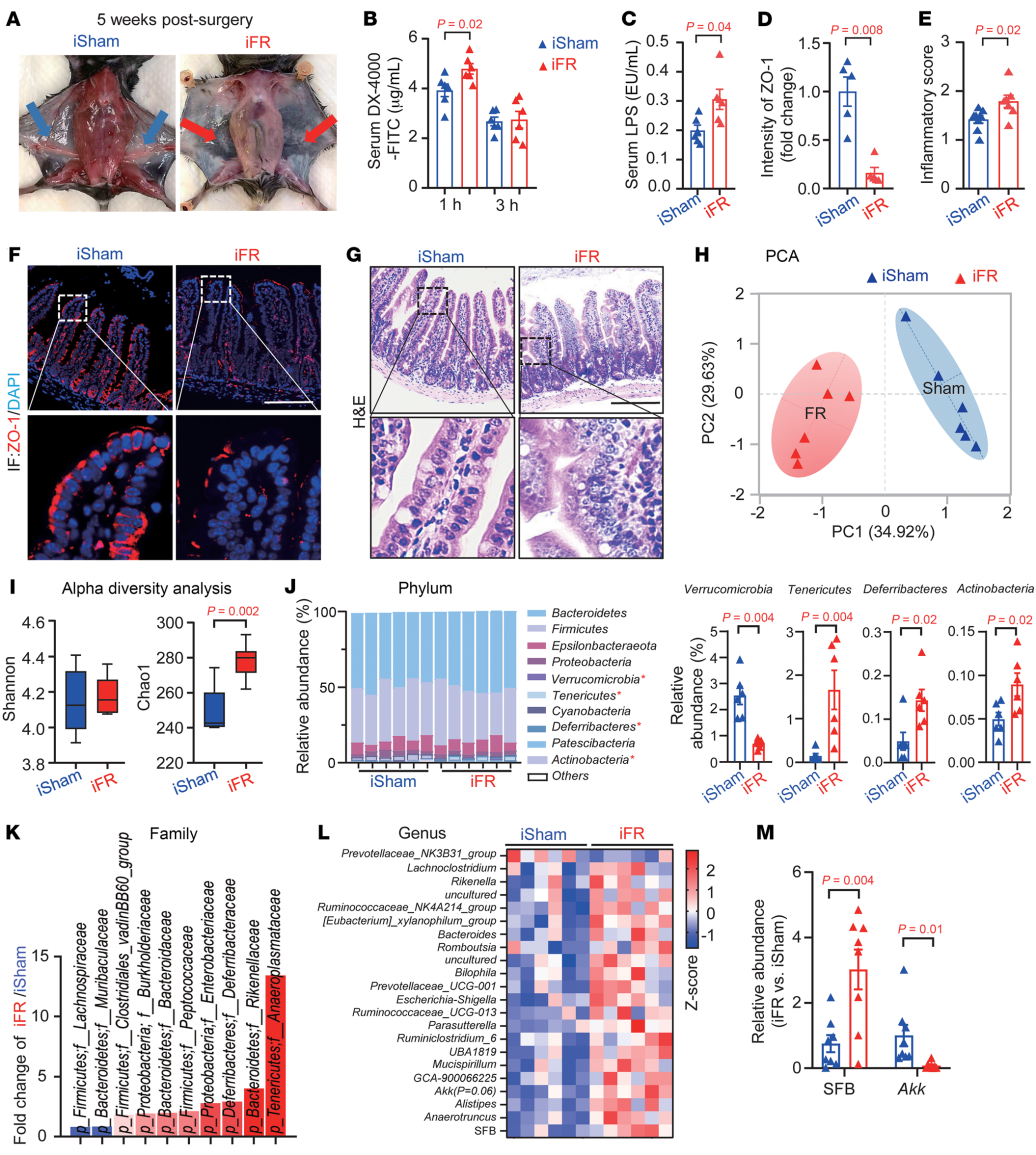
Inguinal subcutaneous white adipose tissue (iWAT) removal alters intestinal functions and gut microbiota homeostasis. Twelve-week-old male C57BL/6J mice underwent surgical inguinal subcutaneous fat removal (iFR) or a sham operation (iSham) for 5 weeks. (**A**) Representative images of the inguinal subcutaneous fat region in iSham mice (blue arrows) and iFR mice (red arrows). (**B**) In vivo gut permeability test (*n* = 6). (**C**) Circulating LPS levels (*n* = 6). (**D**) The fold change of ZO-1 intensity in the ileum (*n* = 5). (**E**) Intestinal inflammatory score in the ileum (*n* = 8). (**F** and **G**) Representative images of immunofluorescent staining of ZO-1 (**F**) and H&E staining (**G**) in the ileum. Scale bar: 100 μm. (**H**–**L**) Microbiota in the cecum were subjected to 16S rRNA sequencing (*n* = 6). (**H**) Principal component analysis (PCA) of gut microbiota composition at the species level. (**I**) α-Diversity analysis of the Shannon index and Chao1 index. (**J** and **K**) Relative abundances of microbial phyla (**J**) and family-level microbiota (**K**) with significant differences shown (actual *P* values are presented in Supplemental Table 6). (**L**) Heatmap displaying significant differences in microbiota at the genus level (actual *P* values are presented in Supplemental Table 6). (**M**) qPCR analysis of segmented filamentous bacteria (SFB) and *Akkermansia* (*Akk*) normalized to the abundance of all bacteria (*n* = 8). Data are presented as the mean ± SEM. Statistical significance was determined using a 2-tailed Mann-Whitney *U* test for **C** and **M**, while other panels used a 2-tailed Student’s *t* test.

To elucidate the role of sWAT in intestinal functions, we conducted surgical bilateral excision of iWAT (the largest subcutaneous fat depot in mice) in 12-week-old male C57BL/6J mice (so-called inguinal fat removal [iFR] mice). Post-surgical assessments confirmed the successful removal of iWAT after 5 weeks (Figure 1A). Five weeks after surgery, the iFR mice exhibited no obvious changes in body weight, food intake, serum glucose level, triglyceride levels, adiponectin and leptin levels, and liver injury (alanine transaminase and aspartate transaminase levels) compared with those that received sham operation (so-called iSham mice), suggesting that acute iWAT loss has no obvious impact on systemic metabolic homeostasis (Supplemental Table 1; supplemental material available online with this article; https://doi.org/10.1172/JCI188947DS1). However, the iFR mice showed a modest increase in gut permeability, as indicated by the higher circulating levels of FITC-labeled dextran (DX-4000-FITC) and LPS (a gut microbiota–derived endotoxin), at weeks 4 and 5, respectively (Figure 1, B and C). Consistently, a significant reduction in the tight junction protein zonula occludens-1 (ZO-1) was observed in the ileum tissue of iFR mice compared with the iSham mice (Figure 1, D and F). iWAT removal led to increased intestinal inflammation (Figure 1, E and G), and increased infiltration of neutrophils (Supplemental Figure 1E). However, iFR and iSham mice had comparable intestinal morphology, including mucosal thickness, tunica mucosa, and villus-to-crypt ratio (Supplemental Figure 1, A–C). In addition, iWAT removal did not affect mucin production and secretion in the goblet cells (Supplemental Figure 1D).

Next, we investigated the impact of iWAT removal on gut microbiota composition. Five weeks after surgery, cecal microbiota were subjected to 16S rRNA gene amplicon sequencing. Principal component analysis (PCA) revealed a distinct microbial community composition between iFR and iSham mice (Figure 1H). Moreover, iWAT removal significantly increased the Chao1 index (but not the Shannon index) of bacterial diversity (Figure 1I), as in naturally aged mice (19, 26). Compared with the iSham mice, a significant increase in the abundance of Verrucomicrobia, Tenericutes, Deferribacteres, and Actinobacteria phyla was observed in the iFR mice (Figure 1J). At the family level, there was a lower abundance of Lachnospiraceae and Muribaculaceae, and a higher abundance of Clostridia_vadinBB60_group and Desulfovibrionaceae (Figure 1K), which has been recently shown to be associated with aging (19, 26). At the genus level, 16S data showed an upregulation of segmented filamentous bacteria (SFB) and a trend of downregulation of *Akkermansia* (*P* = 0.06) in iFR mice (Figure 1L). We further validated the significant changes of SFB and *Akkermansia* by quantitative PCR (qPCR) analysis with a larger sample size (Figure 1M).

**Figure 2:**
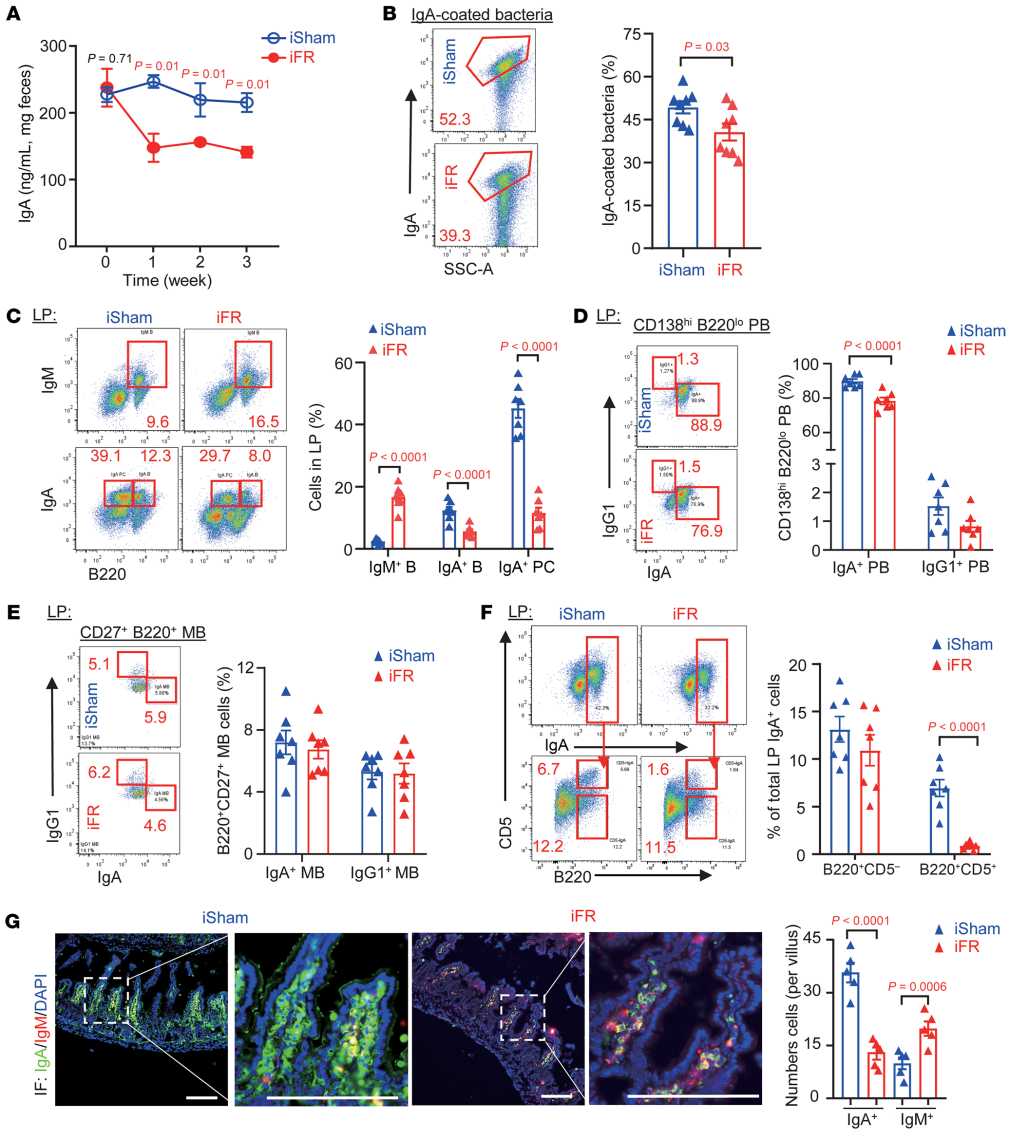
Reduction of IgA^+^ cell populations in the LP of iFR mice. (**A**) Fecal IgA levels from week 0 to week 3 (*n* = 8). (**B**) The percentage of IgA-coated bacteria in the ileal content (*n* = 8). (**C**–**F**) Lamina propria (LP) isolated from the ileum was subjected to flow cytometric analysis. (**C**) Percentage of IgM^+^ B cells (IgM^+^ B), IgA^+^ B cells (IgA^+^ B), and IgA^+^ B220^–^ plasma cells (IgA^+^ PC). (**D**) Percentage of CD138^hi^B220^lo^ plasmablasts (PBs) gated on IgA^+^ PB and IgG1^+^ PB cells. (**E**) Percentage of CD27^+^ B220^+^ memory B cells (MBs) gated on IgA^+^ and IgG1^+^ MBs. (**F**) Percentage of IgA^+^ B220^+^ cells gated on CD5^+^ IgA^+^ B cells and CD5^–^ IgA^+^ B cells. All frequencies of cells were gated from CD45^+^ cells (*n* = 7). (**G**) Immunofluorescent staining of IgA, IgM, and DAPI. The bar chart shows the quantification of IgA^+^ and IgM^+^ cells per villus. Scale bars: 100 μm. (*n* = 5.) Representative images are shown. Data are presented as the mean ± SEM. Statistical significance was determined using a 2-tailed Student’s *t* test.

The expansion of SFB has been linked to IgA deficiency (27), whereas the reduction of *Akkermansia* is associated with aging, metabolic diseases, and intestinal inflammation (28). IgA is the most abundant immunoglobulin produced by intestinal IgA^+^ plasma cells (IgA^+^ PCs), which maintains intestinal microbiota homeostasis and immunity (27, 29). We hypothesized that iWAT removal disrupts intestinal homeostasis by impairing IgA biosynthesis. Immunoassay analysis showed that iFR mice had a gradual decrease in fecal IgA levels (but not fecal IgM nor circulating IgA) (Figure 2A and Supplemental Table 1), accompanied by a dramatic reduction of IgA-coated bacteria in the ileum 5 weeks after surgery (Figure 2B). Taken together, these findings suggest that the intestinal dysfunctions in the iFR mouse model, including deteriorated epithelial barrier function, increased circulating LPS, defective IgA production, and altered gut microbiota composition, are closely associated with the aging condition (12, 30, 31).

### iWAT loss disrupts intestinal IgA production and IgA^+^ cell population homeostasis.

Lamina propria (LP) and Peyer’s patches (PPs) in the small intestine are key sites for the generation of IgA-producing cells (32). We measured the immune cell populations related to IgA production in the small intestines of the mice 5 weeks after surgery. In the LP, frequencies of IgA-producing cells (IgA^+^ B220^+^ B cells [IgA^+^ B cells] and IgA^+^ PCs) were reduced by iWAT removal (Figure 2C). Consistently, iWAT removal led to a lower frequency of IgA^+^CD138^hi^ B220^lo^ plasmablasts (IgA^+^ PBs) (Figure 2D). In contrast, iWAT removal had no obvious impact on IgG1^+^ PBs, IgA^+^ memory B cells (MBs), and IgG1^+^ MB populations (Figure 2, D and E), but increased IgM^+^ B cells (Figure 2C). The reduction in IgA^+^ B cells was mainly related to fewer CD5^+^IgA^+^ B subset cells but not CD5^–^IgA^+^ B subset cells (Figure 2F). Notably, CD5^+^IgA^+^ B subset cells derived from IgM-expressing cells can migrate to LP, where they differentiate into IgA-producing PCs, whereas CD5^–^IgA^+^ B subset cells are activated by T cell–dependent antigens derived from organized lymphoid tissue in the PPs (33, 34). Consistent with the flow cytometry analysis, immunofluorescent staining also revealed a significant decrease and increase of IgA^+^ cells and IgM^+^ cells, respectively, within the villi of the small intestine of iFR mice (Figure 2G).

iWAT removal did not alter the numbers of PPs on the small intestine (Supplemental Figure 2A). Unlike the changes in the LP of iFR mice, the majority of IgA-producing-related B lymphocyte populations, including unswitched B cells (B220^+^ gated IgM^+^IgD^+^ cells), IgM^+^ B cells, and IgA-producing cells in PPs, were similar between the 2 groups, despite a reduction in IgA^+^ PB cells (Supplemental Figure 2, B–E). Immunofluorescent staining revealed that the percentage of IgA^+^ and IgM^+^ cells in the PPs was comparable between iFR and iSham mice (Supplemental Figure 2F). Furthermore, IgA^+^ B cells and IgA^+^ PCs in mesenteric lymph node and spleen were similar between iFR and iSham mice (Supplemental Figure 3, A and B).

Intestinal IgA class switch recombination primarily occurs in PPs via a T cell–dependent and –independent manner. Upon interaction with activated T follicular helper (Tfh) cells within PPs, B cells are activated and become germinal center B cells that express activation-induced cytidine deaminase (AID) to mediate class switch recombination and somatic hypermutation (35). In addition, Tfh cells are also important for the generation of memory B cells (36). Flow cytometry analysis showed that the frequency and number of B220^+^GL-7^+^ germinal center B cells and CXCR5^+^PD-1^+^ Tfh cells (gated on CD4^+^ cells) in PPs were indistinguishable between iFR and iSham control mice. There was no difference in Th17 and regulatory T cell populations as well as AID expression in PPs between the 2 groups (Supplemental Figure 4, A–E).

B cells themselves cannot produce retinoic acid (RA) and require an external source of RA and other cofactors such as TGF-β1, APRIL, and BAFF for their IgA class switching and IgA production (37). A subset of LP CD11b^+^CD103^+^ dendritic cells produce RA via retinal dehydrogenase type 2 (*Aldh1a2*), which are known to mediate T cell–independent IgA class switching (38). Flow cytometry analysis revealed that the number of this dendritic cell population and its RA production ability were similar between the iFR and iSham mice (Supplemental Figure 5, A and B). Additionally, mRNA expression of the RA biosynthesis enzymes, including *Aldh1a1*, *Aldh1a2*, and *Aldh1a3*, was not altered in the small intestine of iFR mice (Supplemental Figure 5C). Intestinal expression of the conventional IgA class switching–promoting factors such as *Tnfsf13*, *Tnfsf13b*, *Tgfb1*, and *Il10* (which mediate the T cell–independent pathway) was similar among the 2 groups (Supplemental Figure 5C). There was no difference in TGF-β1^+^ T cell and TGF-β1^+^ myeloid cell populations within LP (Supplemental Figure 5, D and E). These findings suggest that the machinery or factors mediating intestinal T cell–dependent and –independent IgA production are intact.

Apart from PPs and mesenteric lymph node (MLN), in situ IgA class switching of B cells and AID expression have also been detected in LP (39). We measured the expressions of the molecular markers for class switch recombination, including Iμ-Cα (expressed by the IgH locus after class switch recombination), Iα-Cμ transcripts (α*CT*) from the circle DNA of IgA switching, α-germline transcripts (α*GT*), and AID (39, 40), in the LP by semiquantitative PCR, qPCR, and immunohistochemical staining. These analyses showed a substantial reduction of these class switching markers in the LP of iFR mice compared with iSham controls (Supplemental Figure 1, F–I). In addition, the frequencies and numbers of unswitched B cells and IgM^+^ B cell population in the LP were higher than those in the iSham mice (Supplemental Figure 1J and Figure 2C).

To generalize the findings, we assessed the impact of iWAT removal on intestinal B cell homeostasis and microbiota composition using another mouse strain, BALB/c mice. C57BL/6J and BALB/c mice exhibit differences in immune responses and gut microbiome compositions even under identical housing conditions (41). iWAT removal also led to increased gut permeability, decreased fecal IgA levels and IgA-coated bacteria, and reduction of IgA^+^ B cells and IgA^+^ PCs in the LP of BALB/c mice (Supplemental Figure 6). Additionally, iWAT removal shifted gut microbiota composition in BALB/c mice (Supplemental Figure 7, A–F). Although fat removal led to some differences in gut microbiota alterations between C57BL/6J and BALB/c mice, the altered phyla, families, and genera that overlap between the 2 strains exhibited the same directionality in terms of fold change in comparison with their corresponding iSham controls (100% for phyla, 67% for families, and 83% for genera) (Supplemental Figure 8, A and B). Overall, our findings suggest that iWAT removal disrupts intestinal IgA biosynthesis and induces gut microbiota dysbiosis in rodents.

### iWAT removal diminishes SFB-induced intestinal IgA responses.

While IgA deficiency is known to increase SFB expansion (27), SFB triggers Th17 cell activation and IgA responses in the intestine (42, 43). Next, we investigated whether iWAT removal selectively modulates SFB-induced immune response in the gut. To this end, C57BL/6J mice that received iWAT removal and sham operation were subjected to antibiotic (ABX) treatment to eliminate intestinal SFB, followed by recolonization with SFB to induce SFB-specific Th17 cells and IgA responses (Supplemental Figure 9A). There were 5 groups: (a) iSham, (b) iSham-ABX, (c) iFR-ABX, (d) iSham-ABX+SFB, and (e) iFR-ABX+SFB. qPCR analysis confirmed that ABX treatment successfully reduced intestinal SFB to a virtually undetectable level in iSham-ABX and iFR-ABX mice. SFB levels increased by around 300-fold in iSham-ABX+SFB and iFR-ABX+SFB mice compared with iSham mice (Supplemental Figure 9B). As expected, recolonization of SFB induced Th17 cell population in PPs and IL-17 secretion in the LP lymphocytes (LPLs) of iSham-ABX+SFB mice, and such induction was further potentiated by iWAT removal (Supplemental Figure 9, C–E). Consistent with the previous studies (27, 42), recolonization of SFB induced intestinal IgA responses (iSham-ABX mice vs. iSham-ABX+SFB mice), as exemplified by increased number of IgA secretory cells in LP by ELISpot and IgA secretion in conditional medium from LPLs, increased fecal IgA, and upregulated gene expression of *Aicda* (Supplemental Figure 9F and Supplemental Figure 10, D–G). However, iWAT removal blunted this SFB-induced IgA response in the intestines (i.e., 0 weeks vs. 1 week after iWAT removal and iSham-ABX+SFB mice vs. iFR-ABX+SFB mice) (Supplemental Figure 10, A–G). iWAT removal had no obvious effect on SFB-induced upregulation of *Tnfsf13* and *Tnfsf13b* and *Pigr* (the IgA transporter) (Supplemental Figure 9F). The effect of iWAT removal on Th17 and IgA responses appeared independent of intestinal barrier functions, as *ZO-1* expression was comparable among iSham-ABX+SFB mice and iFR-ABX+SFB mice (Supplemental Figure 9F). Taken together, our findings suggest that iWAT removal selectively diminishes SFB-induced IgA responses, and such effect is independent of Th17 cells or barrier function.

### iWAT protects intestinal function after C. rodentium infection in mice.

IgA is also known to protect the intestine from pathogenic microorganism infection (44). Thus we investigated the impact of iWAT removal on intestinal infection against *Citrobacter rodentium*, a well-established murine model pathogen to investigate human intestinal diseases, including enteropathogenic *Escherichia coli* and enterohemorrhagic *E.* *coli* infections (45). To this end, iFR and iSham mice were orally gavaged with *C.* *rodentium* (Supplemental Figure 11A). Upon infection for 5 and 10 days, fecal IgA antibody against *C.* *rodentium* was significantly induced in iSham mice. Such IgA antibody response was blunted by iWAT removal (Supplemental Figure 11B). The IgA reduction led to increased *C.* *rodentium* abundance in the fecal samples of iFR mice (Supplemental Figure 11C). The changes were accompanied by reduced IgA-coated bacteria, IgA^+^ B cells, and IgA^+^ PCs in the LP of iFR mice (Supplemental Figure 11, D and E). iWAT removal also led to more severe inflammatory response and immune cell infiltration and reduced ZO-1 protein expression in the intestine, as well as more body weight loss (Supplemental Figure 11, F–H). On the other hand, IgA^+^ B cells and IgA^+^ PCs in PPs were comparable between iFR and iSham mice upon infection (Supplemental Figure 11, H–J). Taken together, these findings suggest that iWAT removal impairs pathogen-induced IgA response and hence increases pathogen infection susceptibility and severity.

### Adipocyte-derived RA promotes IgA production and class switching in B lymphocytes.

As an endocrine organ, WAT communicates with the distal tissues via adipokines (5, 7). We examined whether iWAT-derived adipokines promote IgA class switching and IgA production using ex vivo approaches. Splenic unswitched B cells were cultured with iWAT-derived conditioned medium (iWAT-CM) or RPMI 1640 medium (Supplemental Figure 12A). Treatment with iWAT-CM increased the proportion of IgA^+^ cells and IgA secretion and upregulated the IgA class switching markers, including *Aicda*, α*CT*, α*GT*, and Iμ-Cα, in comparison with those unswitched B cells treated with RPMI 1640 plain medium (Supplemental Figure 12, B–E). Furthermore, CM from the fraction of mature adipocytes of iWAT (Adipocyte-CM) exerted a more potent effect on IgA production and IgA class switching than that from the stromal vascular fraction (SVF-CM) and reached a magnitude similar to that in the iWAT-CM (Supplemental Figure 12, B–E). The minimal effect of SVF-CM might be due to its derived factors and/or copurified adipocytes.

**Figure 3:**
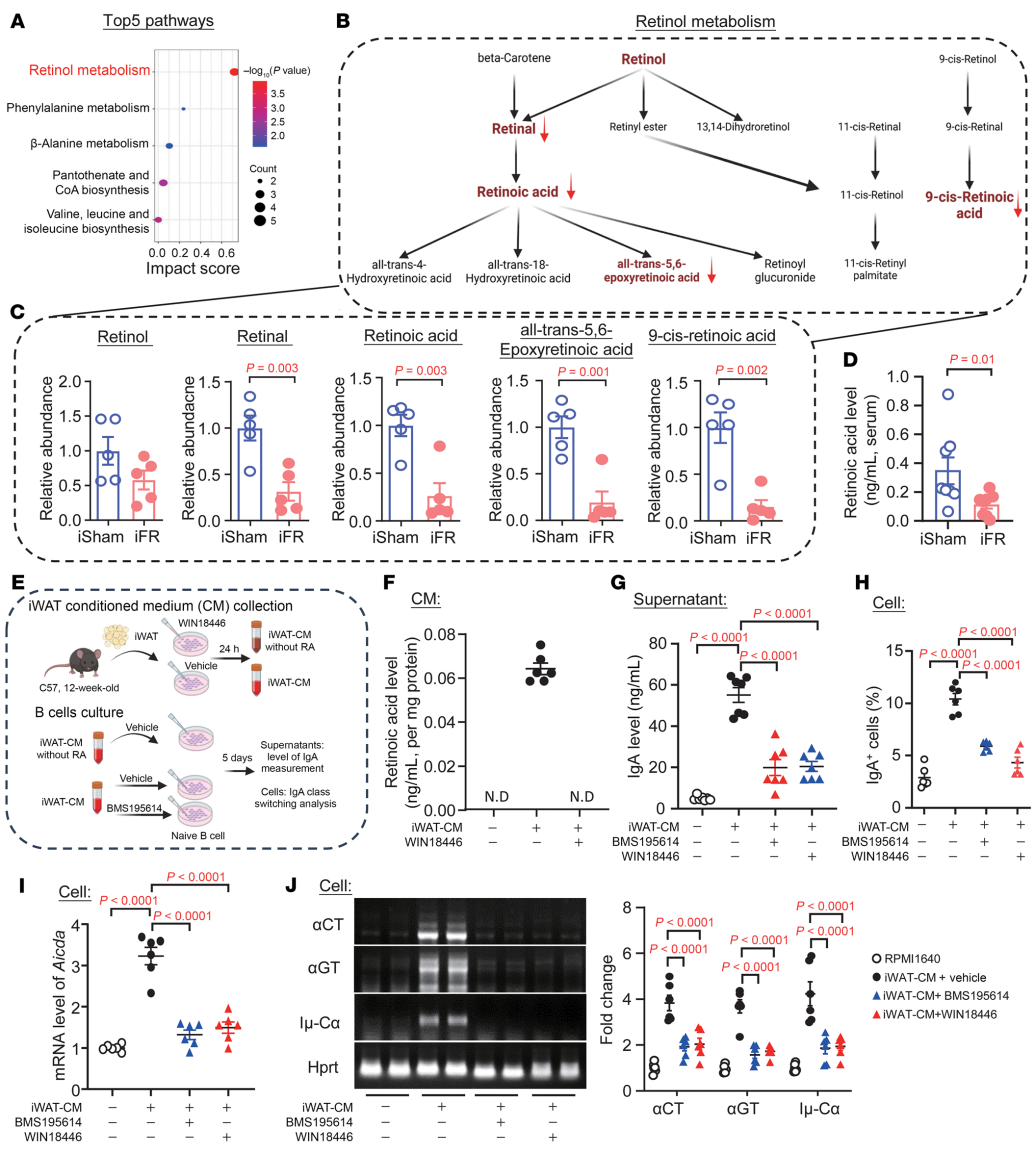
iWAT-secreted retinoic acid induces IgA class switching and IgA production in B cells. (**A**–**D**) Serum samples from iFR and iSham mice were collected for untargeted metabolomics analysis. (**A**) Pathway enrichment analysis from metabolomics data. (**B**) Schematic diagram of metabolites in the retinol metabolism pathway, with detected metabolites highlighted in red. (**C**) Relative abundance of the detected metabolites in **B** (*n* = 5). (**D**) Liquid chromatography–tandem mass spectrometry (LC-MS/MS) analysis of retinoic acid (RA) levels (*n* = 8). (**E**–**J**) In vitro culture of unswitched B cells in conditioned medium (CM) from iWAT, iWAT pretreated with WIN18446, and iWAT-CM pretreated with BMS195614, and RPMI 1640 medium as a vehicle control (*n* = 6). (**E**) Schematic diagram of the in vitro experiments (*n* = 6). (**F**) LC-MS/MS analysis of RA levels. (**G**) IgA level in the supernatants at day 5. (**H**) Flow cytometric analysis of IgA^+^ cell frequency. (**I**) Relative *Aicda* mRNA level, normalized to *Hprt*. (**J**) Representative images of semi-qPCR analysis with quantitative analysis shown in the right panel. Data are presented as the mean ± SEM. Statistical significance was determined using a 2-tailed independent Student’s *t* test for **A**, **C**, and **D**, a 2-tailed Mann-Whitney *U* test for **D**, and 1-way ANOVA with Tukey’s multiple-comparison test for **F**–**J**.

To identify the potential iWAT-derived adipokines that regulate intestinal IgA biosynthesis, we conducted untargeted metabolomics in the serum of iFR and iSham mice. A total of 214 differentially abundant metabolites were identified (log_2_[fold change] > 1 or < –1 and adjusted *P* value < 0.05) between the 2 groups. Pathway analysis showed that retinol metabolism was the pathway most significantly altered by iWAT removal (Figure 3, A and B). Even though the retinol level remained unchanged, its downstream oxidized products, including retinal, RA, and all-*trans*-5,6-epoxy-RA, were significantly downregulated in the iFR mice (Figure 3C). The reduction of RAs was further confirmed through use of a targeted liquid chromatography–tandem mass spectrometry analysis (Figure 3D).

RA, in conjunction with other cytokines such as TGF-β1, lactoferrin, and IL-5, promotes IgA class switching and IgA biosynthesis within the intestinal microenvironment (37, 46, 47). However, the potential contribution of extra-intestinal RA to intestinal IgA biosynthesis remains unexplored. Given that intestinal RA biosynthesis appeared unaffected in the iFR mice, we examined whether RA from iWAT is responsible for the IgA class switching and IgA production. To this end, iWAT was incubated with RPMI 1640 with 1% BSA in the presence or absence of RA synthesis inhibitor (WIN18446) for 48 hours, and their derived CM was used to culture B cells. We were able to detect RA in the iWAT-CM (Figure 3F). Moreover, unswitched B cells underwent IgA class switching and produced more IgA upon incubation with iWAT-CM (Figure 3, E–J), and the effect of iWAT-CM was largely blocked by the inhibition of RA synthesis (Figure 3, E–J). Pretreatment with the RA receptor α (RARα) antagonist BMS195614 also abolished the promoting effect of iWAT-CM on IgA production and its class switching in the B cells (Figure 3, E–J). Likewise, CM derived from 3T3-L1 mature adipocytes also increased IgA production and induced IgA^+^ cell population in an RA-dependent manner (Supplemental Figure 12, F–H).

**Figure 4:**
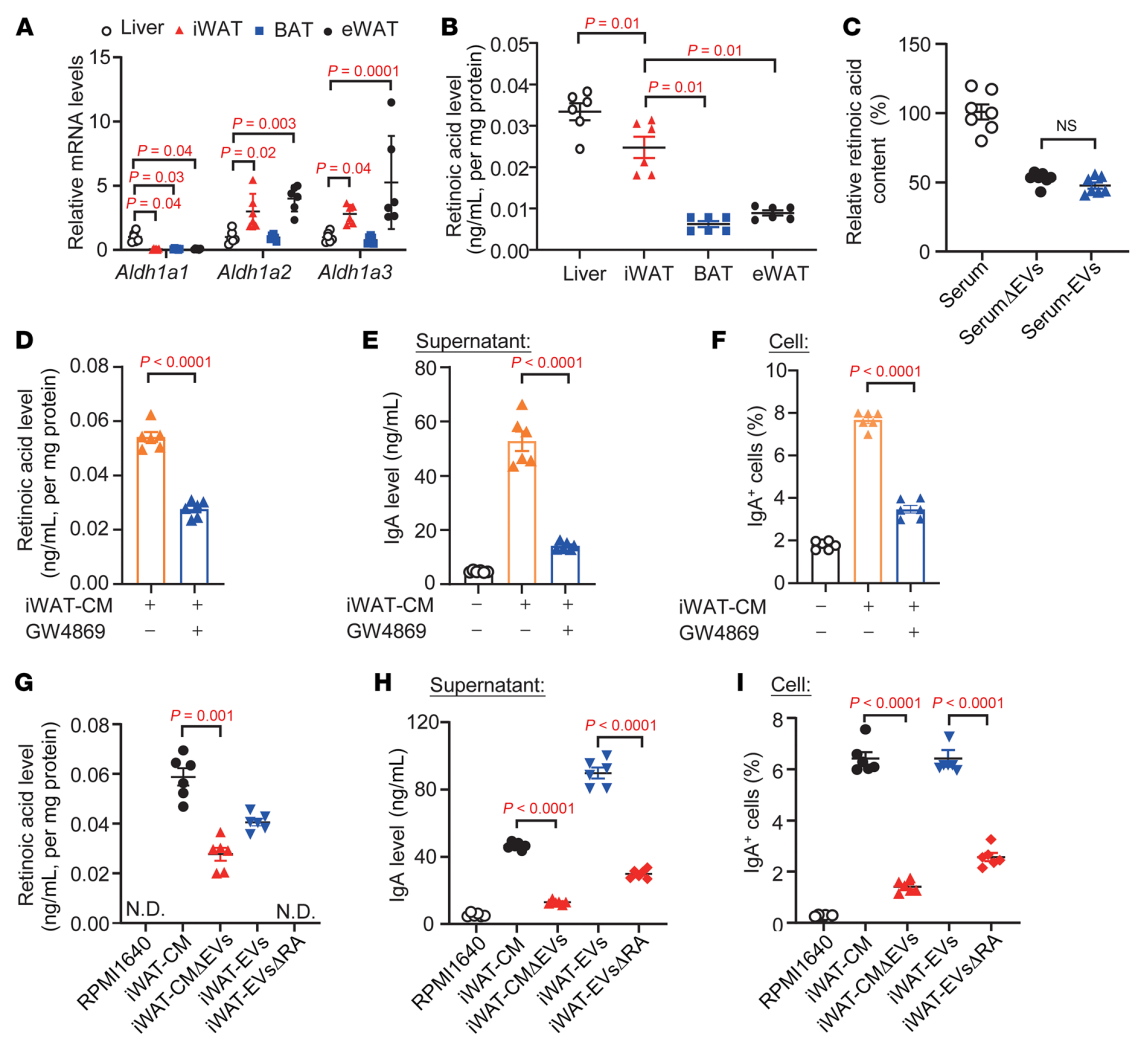
RA as cargo in the iWAT-derived extracellular vesicles promotes IgA class switching and IgA production in B cells. (**A**) qPCR analysis of *Aldh1a1*, *Aldh1a2*, and *Aldh1a3* gene expression in the liver, iWAT, brown adipose tissue (BAT), and eWAT isolated from 12-week-old male C57BL/6J mice. The targeted genes were normalized with *Gapdh* and expressed as fold change relative to liver expression (*n* = 6). (**B**) LC-MS/MS analysis of RA levels in the liver, iWAT, BAT, and eWAT (*n* = 6). (**C**) RA levels in the serum, serum without extracellular vesicles (SerumΔEVs), and serum EVs. The graph represents the percentage of RA normalized to whole serum (*n* = 6). (**D**–**F**) CM from iWAT (iWAT-CM) and iWAT pretreated with GW4869 were used to culture unswitched B cells for 5 days (*n* = 6). (**D**) RA levels in the medium. (**E**) IgA level in the supernatant. (**F**) Frequency of IgA^+^ cells. (**G**–**I**) iWAT-CM, iWAT-derived EVs (iWAT-EVs), and iWAT pretreated by WIN18446 (iWAT-EVsΔRA) and EVs removed from iWAT-CM via ultracentrifugation (iWAT CMΔEVs) were used for culture of unswitched B cells for 5 days (*n* = 6). (**G**) RA levels in indicated samples. (**H**) IgA levels in the supernatants. (**I**) Frequency of IgA^+^ cells. Data are presented as the mean ± SEM. Statistical significance was determined using 1-way ANOVA with Tukey’s multiple-comparison test. N.D., nondetectable.

Apart from the liver, WAT has been implicated in the regulation of RA homeostasis through both biosynthetic and degradative pathways (48). Consistent with previous studies (49, 50), the RA biosynthetic genes (*Aldh1a1*, *Aldh1a2*, and *Aldh1a3*) and RA were detectable in the iWAT of C57BL/6J mice (Figure 4, A and B). Notably, the RA level in iWAT was highest among the fat depots, only 30% lower than that in the liver (Figure 4B). On the other hand, the gene expressions of *Aldh1a2* and *Aldh1a3*, but not *Aldh1a1*, in iWAT were higher than in the liver (Figure 4A). These data suggest that *Aldh1a2* and *Aldh1a3* might be major isoforms responsible for RA biosynthesis in iWAT.

In addition to metabolomics, we also explored whether the circulating proteome profile was altered following iWAT removal. Proteomics analysis identified approximately 1,000 proteins in the serum, but there were only 59 differentially expressed proteins between the 2 groups (fold change > 1.2 or < –0.2 and adjusted *P* value < 0.05), and none of them have been reported to be related to IgA production or class switching (Supplemental Figure 13). We used ELISA to measure circulating B cell activation factor (BAFF) and TGF-β1, the two proteins that were shown to be derived from WAT and to promote IgA production (35, 51, 52). There was no difference in BAFF level between iFR and iSham mice, while TGF-β1 level was increased instead of decreased in iFR mice (Supplemental Table 1). Taken together, these findings indicate that the regulatory effect of iWAT on intestinal B cell function and IgA production is potentially due to RA instead of the secretory protein factors from iWAT.

### RA as a key EV cargo derived from iWAT to control intestinal homeostasis in mice.

Encapsulation of metabolites within EVs enhances their tissue targeting and stability. Adipocytes are known to produce EVs to communicate with distal organs in response to environmental and nutritional changes. In addition, RA is not stable in circulation and is hydrophobic. We hypothesized that RA from iWAT carried by EVs increases its stability and targeting to the small intestine. First, we examined whether RA could be found in iWAT-EVs and, if so, in which types of EVs. We fractionated iWAT-EVs into microvesicles (550 nm on average), exosomes (420 nm on average), and the apoptotic bodies (1,080 nm on average) by differential ultracentrifugation. Nanoparticle tracking analysis and immunoblotting analysis confirmed the successful fractionation, as revealed by their size distribution and by enriched expression of the apoptotic marker cleaved caspase-3 in the apoptotic bodies and of adiponectin in the smaller EVs, as previously reported (53). The majority of RA was found in microvesicles and exosomes; RA was barely detectable in the apoptotic bodies (Supplemental Figure 14, A–C). Given the low abundance of RA in the apoptotic bodies, we excluded them in all subsequent experiments by filtering the iWAT-EVs using a 0.22 μm filter. The purity of iWAT-EVs without the apoptotic bodies was confirmed using a transmission electron microscope and by measurement of the EV markers CD63, HSP70, and adiponectin, as well as size distribution analysis (Supplemental Figure 14, D–G). Proteomic analysis also detected multiple EV markers but not the Golgi matrix protein GM130 or the mitochondrial protein translocase of outer mitochondrial membrane 20 (TOM20) or the apoptotic markers in the filtered iWAT-EVs (Supplemental Table 3). “iWAT-EVs” hereafter refers to those without the apoptotic bodies (see Methods).

The physiological relevance of the above findings is further supported by the finding that around 50% of circulating RA was carried by EVs of C57BL/6J mice (Figure 4C). RA in serum EVs was decreased in the iFR mice compared with iSham mice, indicating that iWAT is one of the contributors of circulating EVs-RA (Supplemental Table 1). Ex vivo, inhibition of EV biosynthesis in iWAT using GW4869 reduced the RA level in iWAT-CM by half and significantly dampened the potentiating effects of iWAT-CM on IgA production and IgA^+^ cell population (Figure 4, D–F). Depleting EVs via ultracentrifugation also led to around 60% reduction of RA in iWAT-CM (Figure 4G). In vitro experiments showed that the potentiating effects of iWAT-CM on IgA production and IgA^+^ cell population were diminished by EV depletion (Figure 4, H and I).

**Figure 5:**
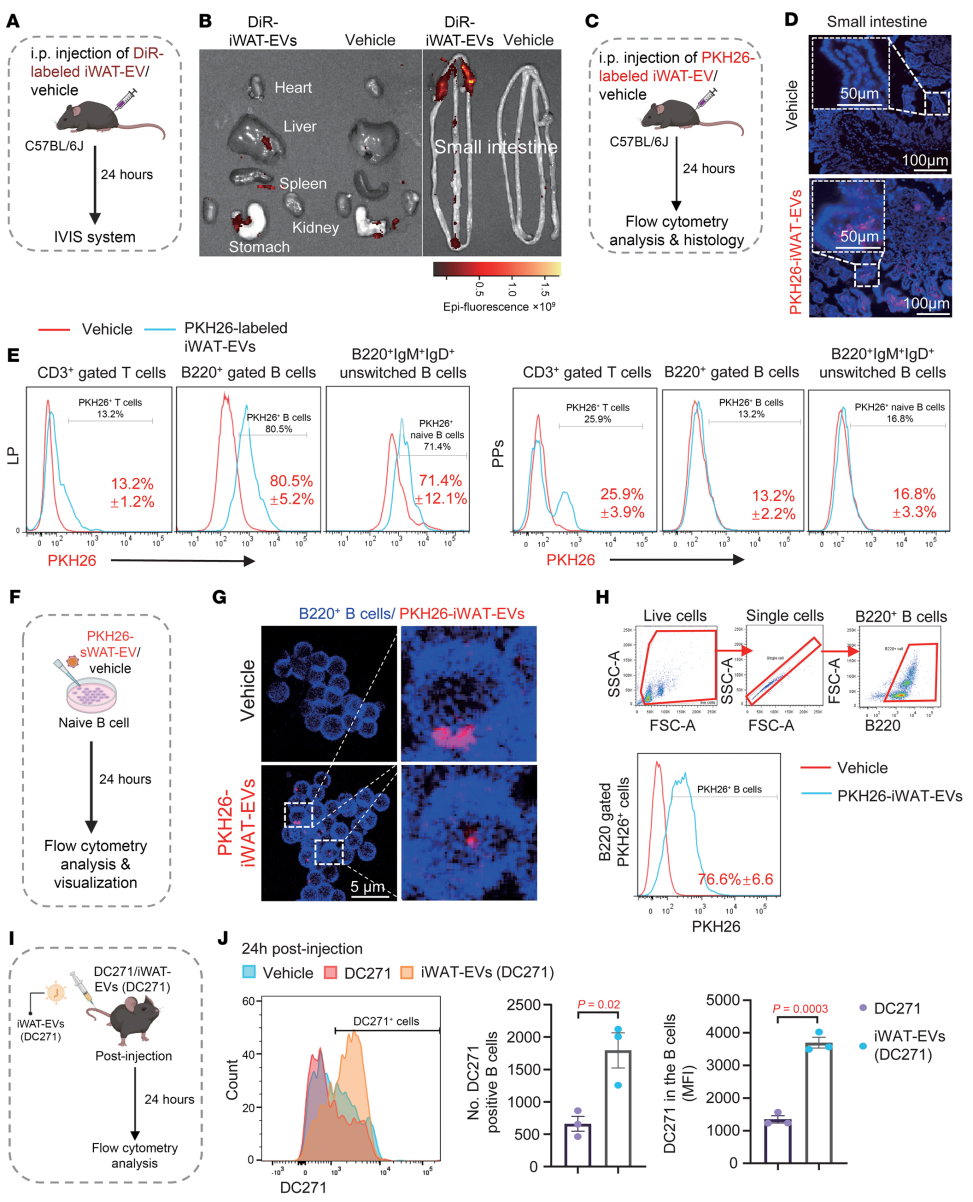
Enrichment of iWAT-EVs in LP and their uptake into B cells. (**A**) Schematic diagram of the DiR-labeled iWAT-EVs biodistribution experiment. (**B**) Visualization of DiR-labeled iWAT-EVs in various organs. (**C**) Schematic diagram of PKH26-labeled iWAT-EV uptake in mice. (**D**) Visualization of PKH26-labeled iWAT-EVs in the small intestine. Scale bars: 50 μm/100 μm. (**E**) Percentages of PKH26^+^ signals in the indicated cells from the LP and PPs. All frequencies of cells were gated from CD45^+^ cells. (**F**) Schematic diagram of the PKH26-labeled iWAT-EV uptake experiment in unswitched B cells (*n* = 3). (**G**) Representative images of PKH26-labeled iWAT-EVs in unswitched B cells. Scale bar: 5 μm. (**H**) The frequency of B220^+^PKH26^+^ cells. (**I**) Schematic diagram of in vivo iWAT-EVs (DC271) B cell uptake experiment (*n* = 3). (**J**) Flow cytometric analysis of DC271^+^ B cells in the ileum 24 hours after injection. Results are shown from 1 experiment, representing at least 2 independent experiments. Data are presented as the mean ± SEM. Representative images are shown. Statistical significance was determined using a 2-tailed Student’s *t* test for **J**.

To determine whether iWAT-EVs transport to the small intestine, we labeled iWAT-EVs with DiR (infrared fluorescence dye DiOC18) and intraperitoneally injected them into C57BL/6J mice (Figure 5, A and B). After 24 hours, DiR signals were detectable in the small intestine, liver, and stomach (Figure 5B). To investigate whether iWAT-EVs target the immune cells of LP and PPs, C57BL/6J mice were intraperitoneally injected with iWAT-EVs labeled with PKH26 (Figure 5, C and E). Flow cytometry analysis revealed that iWAT-EVs were present in the B220^+^ B cells and unswitched B cells isolated from LP, but were barely detectable in the CD3^+^ T cells in the LP and these immune cells from the PPs (Figure 5, D and E). Consistently, iWAT-EVs were incorporated into the unswitched B cells in vitro (Figure 5, F–H). Furthermore, we showed that intraperitoneal injection of DC271 (a fluorescent RA analog) encapsulated in iWAT-EVs consistently displayed a higher fluorescent signal in mouse serum in comparison with those injected with an equal amount of free DC271 (Supplemental Figure 15, A and B). In addition, DC271 encapsulated in iWAT-EVs more efficiently and selectively transported to the intestinal B cells than the free DC271 (Figure 5, I and J, and Supplemental Figure 15, D and E). We also confirmed that endogenous RA in iWAT-EVs was more stable than free RA when they were incubated in mouse serum in vitro (Supplemental Figure 15F).

**Figure 6:**
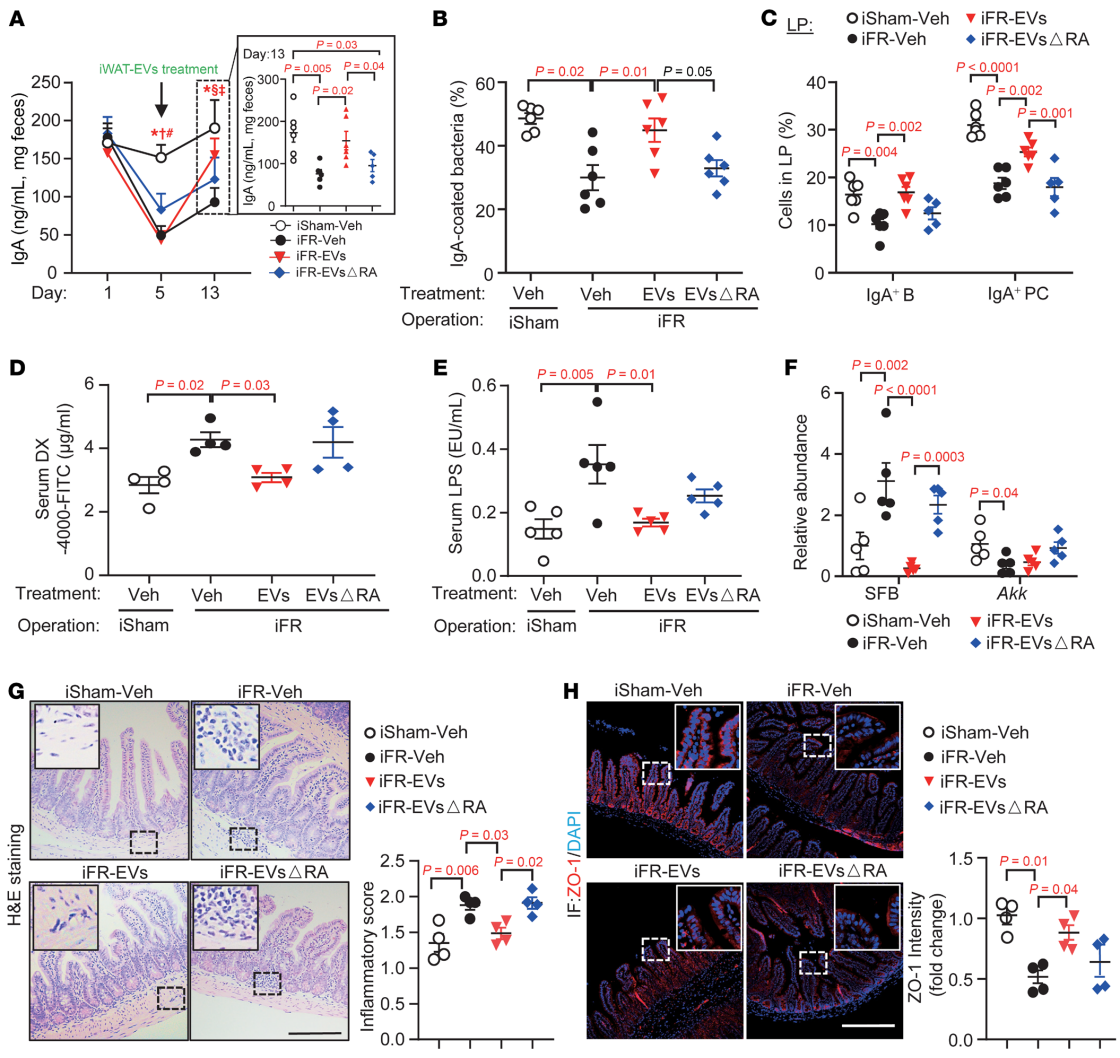
Reversal of dysregulated intestinal functions in mice with iWAT removal by treatment with iWAT-EVs. Twelve-week-old male C57BL/6J mice underwent surgical inguinal fat removal (iFR) or sham operation. Four days after surgery, mice were treated with iWAT-derived extracellular vesicles (iFR-EVs) or EVs depleted of RA (iFR-EVsΔRA) every other day for a total of 5 treatments. (**A**) Fecal IgA levels at day 1, day 5, and day 13 after EV treatments (*n* = 6). Significant *P* < 0.05: *iSham-Veh versus iFR-Veh; ^#^iSham-Veh versus iFR-EVs; ^†^iSham-Veh versus iFR-EVsΔRA; ^§^iFR-Veh versus iFR-EVs; ^‡^iFR-EVs versus iFR-EVsΔRA. (**B**) Percentage of IgA-coated bacteria. (**C**) The frequencies of IgA^+^ PCs and IgA^+^ B cells. All frequencies of cells were gated from CD45^+^ cells. iSham-Veh (*n* = 6), iFR-Veh (*n* = 6), iFR-EVs (*n* = 6), and iFR-EVsΔRA (*n* = 5). (**D**) In vivo gut permeability test (*n* = 4). (**E**) Circulating LPS levels (*n* = 5). (**F**) qPCR analysis of SFB and *Akkermansia*. The targeted microbiota was normalized to the abundance of all bacteria. Data are presented as fold change relative to Sham-Veh (*n* = 5). (**G** and **H**) H&E staining analysis (**G**) and immunofluorescent staining of ZO-1 (**H**), with the right panels showing quantitative analysis. Scale bars: 100 μm. (*n* = 4.) Representative images are shown. Data are presented as the mean ± SEM. Statistical significance was determined using 1-way ANOVA with Tukey’s multiple-comparison test.

Next, we examined the physiological role of iWAT-EVs and their encapsulated RA in intestinal functions. To this end, iWAT-EVs, iWAT-EVs without RA (i.e., EVs collected from iWAT pretreated with WIN18446; so-called iWAT-EVsΔRA), or vehicle were intraperitoneally injected in iFR mice for 13 days, followed by assessment of intestinal IgA homeostasis and functions. Consistent with our previous observations, iWAT removal sharply reduced fecal IgA on day 5 after surgery (Figure 6A). The defective IgA metabolism, indicated by a reduction of fecal IgA, IgA-coated bacteria, IgA-producing cells, and IgA class switching program, was partially reversed by the treatment with iWAT-EVs but not with the iWAT-EVsΔRA in the LP of iFR mice (Figure 6, A–C, and Supplemental Figure 16). In addition, treatment with iWAT-EVs (but not iWAT-EVsΔRA) improved intestinal permeability and inflammation, and reduced SFB expansion (Figure 6, D–H).

There is a possibility that iWAT-EVs indirectly affect IgA production and intestinal functions through modulating microbiota composition. To test this possibility, we examined the effects of iWAT removal on the intestinal system in a pseudo-germ-free iFR mouse model. As expected, ABX treatment led to a significant reduction in total bacterial DNA and bacterial content on the ileum as well as enlargement of the cecum in comparison with the mice treated with PBS (Supplemental Figure 17, B–D). Upon microbiota depletion, fecal IgA and intestinal IgA^+^ cell populations were dramatically reduced in the mice that received sham operation (Supplemental Figure 17, E and F), as previously reported (42, 43). iWAT removal further reduced fecal IgA and IgA^+^ cell populations in LP in the presence of ABX (i.e., iSham-ABX mice vs. iFR-ABX mice) (Supplemental Figure 17, E–G), suggesting that these effects of iWAT are independent of the gut microbiota. In addition, treatment with iWAT-EVs significantly upregulated fecal IgA level and IgA^+^ cell populations in the iFR-ABX mice (Supplemental Figure 17, E–G), suggesting that gut microbiota might not be required for the action of iWAT-EVs in gut. The effect of iWAT-EVs is likely independent of intestinal barrier function, since the tight junction marker ZO-1 was comparable between iSham-ABX, iFR-ABX, and iFR-ABX+iWAT-EVs mice (Supplemental Figure 17H). Overall, our findings show that iWAT-EVs maintain intestinal B cell and IgA homeostasis in a microbiota-independent manner.

### Surgical removal of vWAT has no obvious impact on intestinal function and IgA metabolism.

A recent study demonstrated that visceral WAT (vWAT) produces EVs that target LP and trigger intestinal inflammation in diet-induced obese mice (6). To determine whether vWAT plays a role similar to that of iWAT in intestinal function and IgA homeostasis, we conducted removal of epididymal WAT (eWAT) in C57BL/6J mice and then assessed the intestinal functions. Given its close anatomical location to the gut, we considered investigating the impact of mesenteric WAT removal, but the procedure resulted in high mortality rates and poor postoperative recovery, precluding its inclusion in this study. Five weeks after surgery, eWAT did not regenerate (Supplemental Figure 18A), and its removal did not alter gut permeability and serum LPS concentration (Supplemental Figure 18B and Supplemental Table 2). In addition, eWAT removal did not change the RA level in whole serum and serum EVs (Supplemental Table 2) and had no effect on fecal and circulating IgA level, IgA-coated bacteria in the ileum, and fecal IgM (Supplemental Figure 18, C and D, and Supplemental Table 2). Consistently, there were no differences in different IgA^+^ cell populations and unswitched B cell populations in LP and PPs between the mice that received eWAT removal and sham operation (Supplemental Figure 18, E–G). In addition, we found that iWAT produced more EVs and secreted more RA in EVs than eWAT (Supplemental Figure 19, A and B), which might explain the divergent effects of iWAT and eWAT in intestinal functions. Notably, eWAT removal exerted no obvious effect on energy and lipid metabolism or the liver function biomarker, but modestly reduced fed glucose level and body weight (Supplemental Table 2). In sum, our findings indicate that subcutaneous WAT, but not visceral WAT, regulates intestinal IgA homeostasis and RA metabolism.

### Inhibition of RA secretion from iWAT impairs intestinal IgA and microbiota homeostasis.

**Figure 7:**
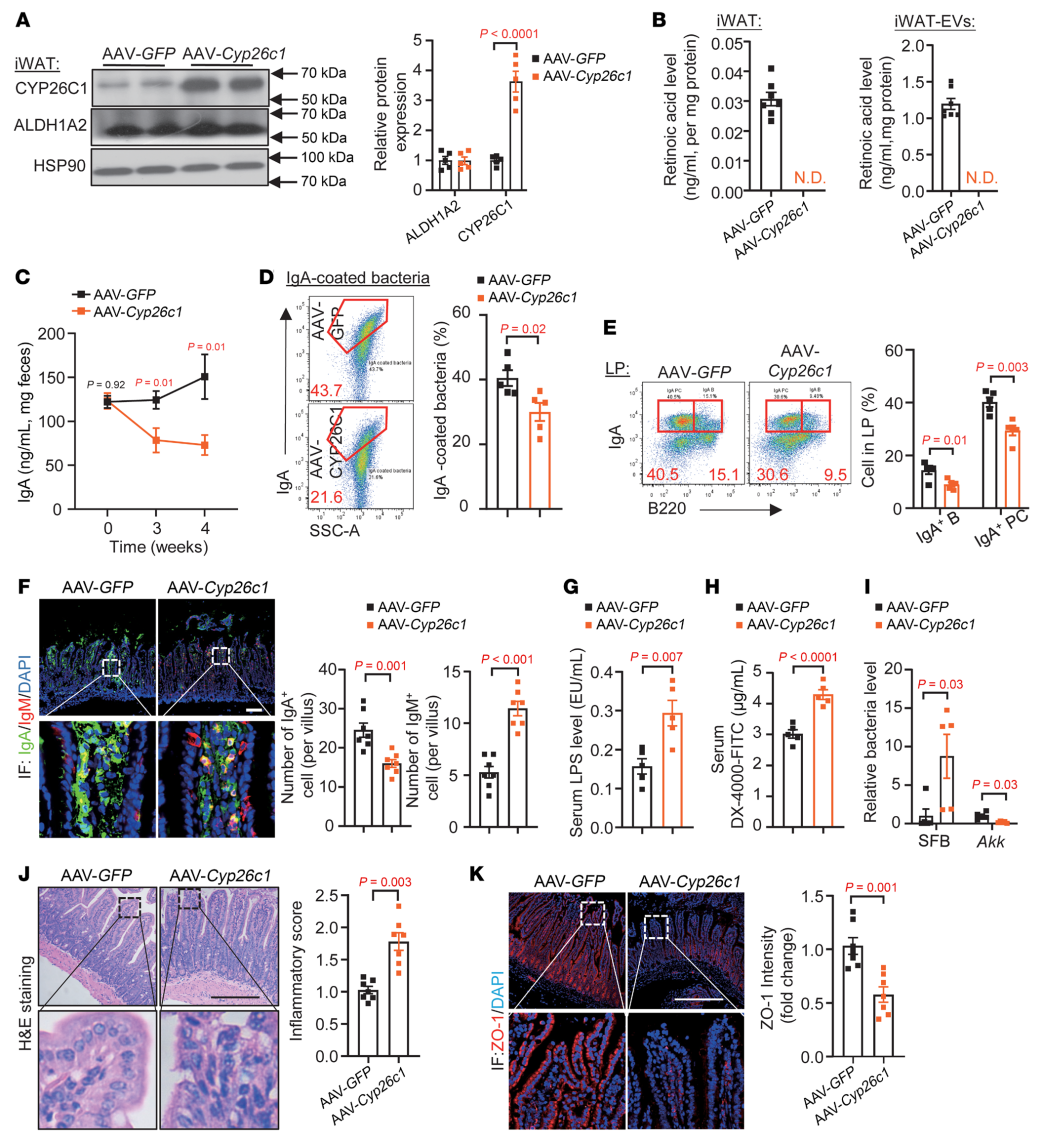
Reduction of IgA^+^ cell population in the LP of mice with AAV-*Cyp26c1* injection into iWAT. Twelve-week-old male C57BL/6J mice were injected with AAV-Rec2-adiponectin-*Cyp26c1* or AAV-Rec2-adiponectin-*GFP* into iWAT for 4 weeks. (**A**) Immunoblotting analysis of CYP26C1, ALDH1A2, and HSP90 expressions in iWAT, presented as fold change relative to that expression in the AAV-*GFP*–injected mice (*n* = 5). (**B**) RA levels in the iWAT and iWAT-EVs (*n* = 6). (**C**) Fecal IgA levels at weeks 0, 3, and 4 after injection (*n* = 5). (**D**) Percentage of IgA-coated bacteria in the ileum. (**E**) Percentages of IgA^+^ cell populations. (**F**) Immunofluorescent staining of IgA, IgM, and DAPI in the ileum. Scale bar: 100 μm. The bar charts on the right quantify IgA^+^ and IgM^+^ cells per villus (*n* = 7). (**G**) Circulating LPS levels (*n* = 5). (**H**) In vivo gut permeability test performed at 3 weeks after AAV injection (*n* = 5). (**I**) qPCR analysis of SFB and *Akkermansia* (*Akk*). Data are presented as fold change relative to that expression in AAV-*GFP* mice (*n* = 5). (**J** and **K**) H&E staining analysis (**J**) and immunofluorescent staining of ZO-1 (**K**), with the right panels showing quantitative analysis. Scale bars: 100 μm. (*n* = 7.) Representative images are shown. Data are presented as the mean ± SEM. Statistical significance was determined using a 2-tailed Mann-Whitney *U* test for **I** (left) and **K**, while other panels used a 2-tailed Student’s *t* test. N.D., non-detectable.

We next sought to directly demonstrate whether the abrogation of iWAT-derived RA impairs intestinal IgA^+^ cells’ homeostasis and functions. Intracellular RA levels are maintained by biosynthetic pathways involving at least 3 isoforms of retinal dehydrogenases (ALDH1A1, ALDH1A2, and ALDH1A3) and the degradative pathway mediated by the cytochrome P450 CYP26 family of catabolic enzymes (including CYP26A1, CYP26B1, and CYP26C1). To reduce iWAT-derived RA, we used adeno-associated virus (AAV) to overexpress CYP26C1 in iWAT of C57BL/6J mice. Four weeks after AAV injection, the expression of CYP26C1 but not ALDH1A2 was dramatically and selectively upregulated in iWAT, but not in eWAT, liver, or ileum, of mice that received AAV-*Cyp26c1* injections compared with mice that received AAV-*GFP* (Figure 7A and Supplemental Figure 20A). This upregulation was accompanied by a reduced RA level in the bulk iWAT lysate, iWAT-EVs, whole serum, and serum EVs (Figure 7B and Supplemental Table 4), without affecting RA levels in eWAT-EVs and liver-EVs (Supplemental Table 4). In addition, iWAT-specific CYP26C1 overexpression had no obvious impact on glucose and lipid profiles or liver function (Supplemental Table 4).

Four weeks after AAV injection, AAV-*Cyp26c1*–injected mice showed a substantial decrease in fecal IgA levels and IgA-coated bacteria (Figure 7, C and D). These changes were associated with a reduction of the IgA-producing cells and IgA^+^ PB cells, and impaired IgA class switching in LP (Figure 7, E and F, Supplemental Figure 20, B–E, and Supplemental Figure 21A). However, the frequencies of the IgA-producing cells in other immune organs, including PPs, spleen, and MLN, were comparable (Supplemental Figure 21, B–D). Increased intestinal permeability and inflammation and incrassated tunica mucosa layer as well as reduction of tight junction protein expression in the ileum were observed in the AAV-*Cyp26c1*–injected mice (Figure 7, G, H, J, and K, and Supplemental Figure 20F). Microbiota α-diversity was increased by iWAT-specific overexpression of AAV-*Cyp26c1* (Supplemental Figure 22A), and several aging-related gut bacterial groups such as *Akkermansia*, *Lactobacillus*, and Clostridia were altered in the mice injected with AAV-*Cyp26c1* (Supplemental Figure 22, C–F, and Supplemental Table 5). As in the iFR model, SFB was markedly upregulated in the mice injected with AAV-*Cyp26c1*, which might be due to intestinal IgA deficiency (Figure 7I and Supplemental Table 5). These findings further underscore the critical role of RA from iWAT in the regulation of intestinal IgA production and homeostasis.

### Impaired RA metabolism and defective intestinal IgA production in aging mice.

**Figure 8:**
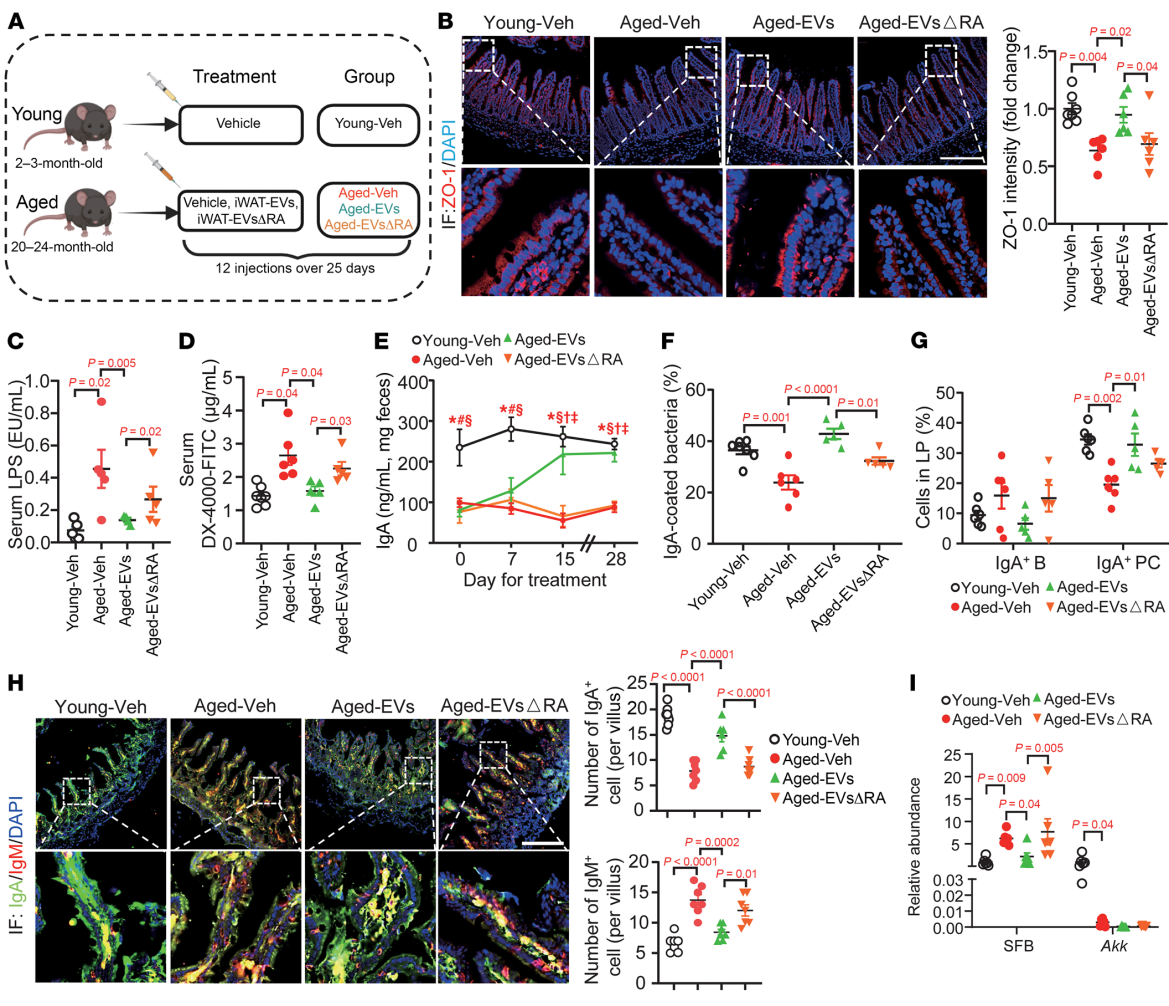
RA in iWAT-EVs rejuvenates intestinal B cells for IgA production in aged mice. (**A**) Schematic depicting the experimental paradigm. (**B**) Immunofluorescent staining of ZO-1 in the ileum, with quantitative analysis of fold change relative to the expression in the iSham-Veh mice (*n* = 7). Scale bar: 100 μm. (**C**) Circulating LPS levels (*n* = 5). (**D**) In vivo gut permeability test (*n* = 5–6). (**E**) Fecal IgA levels at indicated days (*n* = 5–7). Significant *P* < 0.05: *Young-Veh versus Aged-Veh; ^#^Young-Veh versus Aged-EVs; ^§^Young-Veh versus Aged-EVsΔRA; ^†^Aged-Veh versus Aged-EVs; ^‡^Aged-EVs versus Aged-EVsΔRA. (**F**) Percentage of IgA-coated bacteria (*n* = 5–7). (**G**) Percentages of IgA^+^ PCs and IgA^+^ B cells in LP. (**H**) Representative images of immunofluorescent staining of IgA, IgM, and DAPI in the ileum. Scale bar: 100 μm. The right bar charts quantify IgA^+^ and IgM^+^ cells per villus (*n* = 7). (**I**) qPCR analysis of SFB and *Akkermansia* (*Akk*). Data are presented as fold change relative to the expression in the Young-Veh mice (*n* = 6). Data are presented as the mean ± SEM. Statistical significance was determined using 1-way ANOVA with Tukey’s multiple comparisons.

To further explore the physiological relevance of our findings, we examined whether the RA metabolism in iWAT and intestinal IgA metabolism are disrupted in a natural aging mouse model. Male C57BL/6J mice 20–24 months old (equivalent to about 60–70 years old in humans; referred to as “aged” mice hereafter) had intestinal inflammation, increased gut permeability (Figure 8, B–D, and Supplemental Figure 26D), and reduction of fecal IgA and IgA-coated bacteria when compared with 3- to 4-month-old C57BL/6J mice (equivalent to about 20–30 years old in humans; referred to as “young” mice hereafter) (Supplemental Figure 23, A–C). These changes were accompanied by a reduction of IgA^+^ PCs and induction of unswitched B cells in the LP and an increase of germinal center B cells in the PPs from the aged mice (Supplemental Figure 23, D–F). IgA^+^ B cells, LP dendritic cells (LPDCs), and their RALDH enzymatic activity in the LP and Tfh cells in the PPs were unaltered under the aging condition (Supplemental Figure 23, D–F and H–K). In addition, the genes responsible for IgA production (such as *Tnfsf13*, *Tnfsf13b*, *Tgfb1*, and *Il10*) and RA biosynthesis (*Aldh1a1/2/3*) in the LP were similar between the aged and the young mice (Supplemental Figure 23L). Fewer AID-positive cells were observed in the LP but not the PPs of aged mice (Supplemental Figure 23, M and N). These findings suggest that aged C57BL/6J mice also have defective intestinal B cell homeostasis and IgA production.

RA levels in the whole serum or serum EVs were lower in the aged mice than in the young controls (Supplemental Figure 24, A and B). Expression of the RA biosynthetic genes (including *Aldh1a2* and *Aldh1a3*) was downregulated, while the degradative genes (including *Cyp26b1* and *Cyp26c1*) were upregulated, in the iWAT of aged mice (Supplemental Figure 24C). The changes of these RA enzymes were also confirmed by immunoblotting analysis (Supplemental Figure 24D). Consistently, RA in bulk iWAT and iWAT-EVs was virtually undetectable in aged mice (Supplemental Figure 24, E and F). On the contrary, the retinoic biosynthetic and degradative enzymes as well as RA in eWAT and the liver were comparable between the young and aged mice (Supplemental Figure 25, A–F). These findings suggest that a selective reduction of EVs-RA from iWAT might contribute to intestinal dysfunction and defective IgA production in aging.

### Supplementation of iWAT-EVs rescues defective intestinal IgA and microbiota homeostasis in an RA-dependent manner.

Finally, we examined whether replenishment with iWAT-EVs carrying RA rescues the intestinal dysfunctions in aging. Aged male C57BL/6J mice were treated with either iWAT-EVs or iWAT-EVsΔRA isolated from young C57BL/6J mice or PBS as a vehicle control every 4 days for 35 days (Figure 8A). Neither treatment with iWAT-EVs nor iWAT-EVsΔRA conferred any beneficial effects on intestinal morphological changes and inflammation in the aged mice (Supplemental Figure 26, D and E). However, treatment with iWAT-EVs but not iWAT-EVsΔRA led to improvement of gut permeability and reduced serum LPS levels in the aged mice (Figure 8, B–D). Before EV treatment, fecal IgA levels were comparable across all aged treatment groups and significantly lower than those of young mice. Notably, fecal IgA levels gradually increased and were significantly higher in aged mice treated with iWAT-EVs than in aged mice treated with vehicle, an effect not seen in those treated with iWAT-EVsΔRA (Figure 8E). This reinstatement of fecal IgA was accompanied by increased percentages of IgA-coated bacteria (Figure 8F) and IgA^+^ PCs (Figure 8G), an increase of IgA^+^ cells and decreased IgM^+^ cells in the villus (Figure 8H), a reduction of unswitched B cells (Supplemental Figure 26A), and induction of IgA class switching markers in the LP (Supplemental Figure 26, B and C).

We further examined whether treatment with iWAT-EVs has any impact on gut microbiota composition. Principal component analysis indicated a clear separation of the microbiota composition between the young and aged mice, whereas treatment with iWAT-EVs (but not iWAT-EVsΔRA) shifted the microbiota composition toward the young composition (Supplemental Figure 27A). At the phylum level, aging was associated with a significant reduction of Bacteroidetes and Verrucomicrobia, and a significant upregulation of Proteobacteria and Actinobacteria (Supplemental Figure 27, B and C). Importantly, treatment of iWAT-EVs reversed the changes in Bacteroidetes, Actinobacteria, and Proteobacteria phyla under the aging condition (Supplemental Figure 27, B and C). This rejuvenating effect of iWAT-EV treatment on microbiota was also observed at the family and genus levels, at least in part, via RA encapsulated in the iWAT-EVs (Supplemental Figure 27, D and E). In particular, *Lactobacillus* was upregulated in the aged mice, but such upregulation was reversed by iWAT-EV but not iWAT-EVsΔRA treatment (Supplemental Figure 27E). On the other hand, the reduction of *Akkermansia* in the aged mice was unaffected by iWAT-EV treatment or iWAT-EVsΔRA (Figure 8I). Consistent with iFR mice and AAV-*Cyp26c1* iWAT–injected mice, qPCR analysis confirmed that aging-induced upregulation of SFB was recovered by iWAT-EV but not iWAT-EVsΔRA treatment (Figure 8I).

## Discussion

Our findings uncover an essential role of sWAT in maintaining intestinal immunity and microbiota balance in an endocrine manner during aging and infection. The endocrine factor is conveyed by iWAT-EVs containing RA, which promotes IgA class switching in B cells and subsequent IgA production, thereby orchestrating microbiota composition and maintaining healthy mucosa. In natural aging mice, the ability of iWAT-secreting EVs with RA is diminished, resulting in defective intestinal IgA production and microbiota dysbiosis. Replenishment of iWAT-EVs containing RA is sufficient to reestablish the intestinal IgA-producing cells, IgA production, and microbiota homeostasis, while also reducing endotoxemia in the aged mice.

Aging is closely associated with intestinal dysfunction (19, 25). Microbiota dysbiosis promotes intestinal permeability and systemic inflammation in aging (25). Using 3 independent mouse models (mice with iWAT removal, mice with iWAT-specific CYP26C1 overexpression, and naturally aging mice), we demonstrate that sWAT dysfunctions, including diminished RA secretion and sWAT loss, contribute to aging-like intestinal dysfunctions, including reduced IgA production, systemic endotoxemia, microbiota dysbiosis, and pathogen infection. Importantly, these intestinal defects can be largely alleviated by treatment with iWAT-EVs isolated from the young counterparts, suggesting their potential as a potential rejuvenation method. Our studies showed that several well-established aging-related gut microbiota species (19, 25, 54) are significantly altered in the aforementioned 3 models, including *Akkermansia* (decrease in iFR, AAV-*Cyp26c1*, and aged mice), *Lactobacillus* (no change in iFR mice, increase in AAV-*Cyp26c1* mice, and increase in aged mice but counteracted by iWAT-EV treatment), and Clostridia/Clostridiaceae (no change in iFR mice, increase in AAV-*Cyp26c1* mice, and no change in aged mice). Interestingly, the binding of *Lactobacillus* and Clostridia to IgA has recently been shown to be closely associated with aging (19). Another notable bacteria species is SFB, which consistently increased among the 3 mouse models but was downregulated by iWAT-EV treatment in an RA-dependent manner. Removal of iWAT selectively impairs SFB-induced intestinal IgA response. In addition, we observed that iWAT removal increases the abundance of Th17 cells in the intestine of mice with SFB recolonization, but the underlying reasons remain unclear. Aging is known to increase the risk of infection, whereas IgA protects against intestinal pathogenic microorganism infection (44, 45). We showed that reduction of intestinal IgA responses induced by iWAT removal increases the severity of intestinal *C.* *rodentium* infection. Taken together, our findings support not only the role of sWAT in controlling IgA production and its related gut microbiota homeostasis, but also its role against intestinal infection. Nevertheless, intestinal dysbiosis is also known to contribute to functional decline and metabolic diseases in the elderly. It is worth further investigating whether iWAT-EV–mediated rejuvenation of the intestinal functions promotes healthspan in aging.

Consistent with their endocrine role, mature adipocytes exhibit the highest EV production and secretion rate compared with hepatocytes and skeletal muscle cells (55). The EVs derived from healthy adipocytes exert multiple beneficial effects, such as improvement of β cell functions, insulin sensitizing, and anti-inflammation (7, 56, 57). On the contrary, EVs from obese or dysfunctional adipocytes are detrimental to metabolic health, partly because of changes in cargo composition, including miRNA and/or adipokines (58). However, whether the cargo composition, in particular metabolites, of sWAT-EVs is altered in aging and contributes to functional decline is unclear. In this study, we showed that the retinol metabolic pathway is significantly and selectively altered at the gene, protein, and metabolite levels in iWAT of aged mice, leading to reduced iWAT-EVs-RA secretion. Consistent with our findings, the genes facilitating RA biosynthesis, including *Aldh1a2* and *RARB*, and the gene blocking RA biosynthesis (*DHRS3*, which converts retinaldehyde to retinol) are downregulated and upregulated, respectively, in mature adipocytes in sWAT of older human subjects (≥65 years old), as revealed by single-nuclei sequencing (59).

Previous studies showed that the intestinal epithelial cells synthesize RA from dietary vitamin A to generate tolerogenic dendritic cells that produce RA via the Aldh1a enzymes (37, 60). In addition, LPDCs can also produce RA (37, 38, 61). However, we did not observe any change in the population of LPDCs and/or their RALDH enzymatic activity in iFR mice and aged mice (Supplemental Figure 5B and Supplemental Figure 23, I–K). These findings argue that RA derived from the intestinal microenvironment might remain intact in the aforementioned mouse models.

RA is unstable and is rapidly cleared from the circulation. Previous studies demonstrated that RA is bound and transported by serum proteins, including albumin and apolipoprotein A-I (apoAI) (62, 63). Albumin and apoAI are abundantly expressed in the liver but are barely expressed in adipose tissues (Human Protein Atlas; https://www.proteinatlas.org/). Therefore, it is unlikely that RA is carried and transported to the small intestine via albumin. Instead, we demonstrated that more than 50% of RA secreted by iWAT is encapsulated by EVs. Blockage of exosome biogenesis dramatically reduces RA levels in iWAT-CM. A recent study demonstrated that adiponectin encapsulated by EVs exhibited a higher half-life in the circulation than the native adiponectin without EV encapsulation (53, 64). By using isotope-labeled RA, an early study showed that more than 90% of RA is cleared from the circulation within 10 minutes and can be detected in multiple tissues, including liver, adipose tissues, and brain (65). Our in vitro and in vivo experiments showed that RA carried by iWAT-EVs is more resistant to degradation. In addition, our tracing experiments indicated that iWAT-EVs and their encapsulated RA selectively target the B cells within the LP. Interestingly, EVs from obese vWAT also target the LP, where they induce M1 macrophage inflammation via microRNA-155 (6). Our study showed that EVs from vWAT contain lower abundance of RA than those from iWAT, which might explain the absence of an obvious effect of vWAT removal on intestinal functions. Taken in conjunction, these findings suggest that RA carried by EVs not only facilitates their targeting to intestinal LP but also increases their stability during transportation in circulation.

In conclusion, our study identifies a crosstalk between sWAT and the intestinal immune system via the identified adipokine “EVs-RA,” which controls IgA class switching and production in the LP, thereby maintaining gut microbiota homeostasis and permeability. Disruption of this sWAT–intestinal immune system crosstalk triggers intestinal dysfunction in aging. In addition, our results show that iWAT-EVs being taken up by intestinal immune cells (such as B cells) provide a strategy for generating EVs to be used as targeting delivery vehicles for a variety of molecules for intestinal diseases.

### Limitations of the study.

First, the EVs used for the in vivo treatment in iFR and aging models were isolated from bulk sWAT; we thus cannot deduce whether their modulating effects on intestinal B functions are owing to the endocrine factors derived from adipocytes or other adipose tissue–resident cells. However, we showed that 3T3 adipocyte–derived EVs exert a promoting effect on IgA production in the in vitro experiments. Second, it remains unclear which stimuli and physiological conditions trigger release of EVs containing RA from iWAT, although we show that its biosynthesis and secretion decline with age. Third, the regulatory role of sWAT in intestinal IgA metabolism and whether sWAT is capable of producing EVs containing RA in humans, and how sWAT’s ability declines during aging, require further investigation. Our results showed that the depletion of RA in iWAT-EVs led to an approximately 60% reduction in their ability to promote IgA class switching and its production in unswitched B cells compared with intact iWAT-EVs (Figure 4). These data suggest that other factors within EVs and copurification of EVs with other secretory proteins from adipose tissue also contribute to IgA production and class switching. Interestingly, our proteomics detected TGF-β1 and lactoferrin in iWAT-EVs (Supplemental Table 3), which have been reported to enhance IgA response in B cells (47, 66). Whether RA cooperates with these protein factors within EVs and targets the intestinal system for the induction of IgA production needs further investigation. Finally, we observed that iWAT-EVs are selectively and stably transported to the intestinal LP B cells in vivo, yet the underlying mechanism is currently unknown. Further mechanistic studies, such as characterization of surface markers on iWAT-EVs and identification of corresponding receptors on intestinal B cells that mediate the crosstalk between iWAT and intestinal B cells, are warranted.

## Methods

Further information is provided in Supplemental Methods.

### Sex as a biological variable.

Our study examined male mice because male animals exhibited less variability in phenotype. We did not conduct in vivo experiments using female mice, so we cannot claim relevance of our findings beyond male mice.

### Animal studies.

C57BL/6J mice or BALB/c mice were subjected to surgical removal of inguinal subcutaneous fat (iFR) or epididymal fat (eFR). iFR C57BL/6J mice were infected by *Citrobacter rodentium* (ATCC 51459), and these mice were maintained in a pathogen-free environment at Shenzhen Research Institute, Hong Kong Polytechnic University. The experiment was conducted following previously described protocols (67, 68). Briefly, iFR mice were weighed, fasted for 16 hours, and then orally gavaged 1 × 10^9^ CFU of *C.* *rodentium* in PBS. Over a 2-week period, mice were monitored for weight changes, stool consistency (graded on a scale from normal to diarrhea with bleeding), and fecal CFU levels.

For antibiotic treatment experiments, mice were given an antibiotic mixture in drinking water for the specified period as previously described (69). The drinking water with antibiotics was refreshed every 5 days to prevent degradation of the antibiotics due to inactivity.

For SFB colonization experiments, mice were gavaged twice at a 24-hour interval with 0.5 mL of fresh fecal homogenate from SFB-monoassociated mice (provided by Immune Regulation Research Laboratory, Yakult Central Institute, Tokyo, Japan) as previously described (42).

### Flow cytometric analysis.

The gating strategy for the flow cytometric analysis in this study is shown in Supplemental Figure 29, and the detailed protocol for sample processing and antibody staining, as well as a description of the equipment used are available in the Supplemental Methods.

### Statistics.

All data are presented as the mean ± SEM. Each data point derived from qPCR assays represents an average of at least 2 technical replicates. Normality and equality of variances were tested using the Kolmogorov-Smirnov test and Levene’s test, respectively. Statistical significance was determined using a 2-tailed Student’s *t* test for data that were normally distributed (Kolmogorov-Smirnov test, *P* > 0.05) and had equal variance (Levene’s test, *P* > 0.05). For data that were not normally distributed or had unequal variance, a nonparametric 2-tailed Mann-Whitney *U* test was performed. One-way or 2-way analysis of variance (ANOVA) with Tukey’s multiple-comparison test was used for multiple comparisons. Statistically significant differences are indicated by actual value. All statistical analyses were performed using either Prism 8.0 (GraphPad Software) or SPSS.

### Study approval.

All animal experiments were conducted following Hong Kong Polytechnic University (PolyU) guidelines and were approved by the Animal Subjects Ethics Sub-Committee (ASESC) at Hong Kong Polytechnic University or Shenzhen Research Institute, Hong Kong Polytechnic University (ASESC numbers 19-20/55-HTI-R-GRF, 21-22/44-HTI-R-OTHERS, 22-23/318-HTI-R-NSFC, and 23-24/665-HTI-R-CRF).

### Data availability.

The raw proteomics data were uploaded to the ProteomeXchange database with accession PXD066710. The raw 16S rRNA sequencing data were uploaded to the NCBI’s Sequence Read Archive (SRA) database with accession PRJNA1297630 and PRJNA1297624. The raw metagenomics data were uploaded to the SRA database with accession PRJNA1298414 and PRJNA1298794. The raw metabolomics data were uploaded to the MetaboLights database with accession MTBLS12800. The values corresponding to all data points shown in graphs and values behind any reported means are available in the Supporting Data Values Excel file.

## Author contributions

KL performed most of the experiments and drafted the manuscript. PL, WY, MH, PKL, and ZL generated some of the data. JES provided guidance on the experimental design, analyzed data, and revised the manuscript. AWTL performed the analysis of 16S and metagenomics data. GKHS and CWHW advised on gut microbiota sequencing analysis. GHYL provided guidance on data analysis. YC and CBC provided aged C57BL/6J mice and advice on study design. PKS provided advice and technical support for the measurement of retinoic acid. DDZ, CWHW, and AX advised the study and provided animals and reagents. KKYC initiated and supervised the study, provided resources, acquired funding, and wrote and finalized the manuscript.

## Funding support

- Hong Kong Research Grant Council (RGC) Collaborative Research Fund (C5044-23G, to KKYC).
- National Natural Science Foundation of China (91857119 and 92357305, to KKYC).
- Hong Kong Polytechnic University internal funding (P0036848, to KKYC).

## Supplementary Material

Supplemental data

Unedited blot and gel images

Supporting data values

## Version 1 11/17/2025

Electronic publication
