# Supplementary material for: Subcutaneous white adipose tissue–derived extracellular vesicles maintain intestinal homeostasis via IgA biosynthesis in aging mice
Source: J Clin Invest. 2025 Nov 17;135(22):e188947. doi: 10.1172/JCI188947 (PMC12618071; doi:10.1172/JCI188947)
Supplement: Supplemental data [file jci-135-188947-s069.pdf]

Methods and materials

Surgical removal of inguinal subcutaneous fat (iFR) or epididymal fat (eFR). Twelve-week-old male C57BL/6J mice or BALB/c mice underwent inguinal subcutaneous fat removal (iFR) or epididymal fat removal (eFR) under aseptic conditions. Before surgery, a combination of ketamine-xylazine was administered for safe anesthesia in mice. iFR surgery: Both sides of subcutaneous fat were gently pulled out and surgically excised along the inguinal skin. In the iSham control group, both sides of subcutaneous fat were pulled out but not excised.

eFR surgery: Both sides of eWAT were accessed via a mid-ventral abdominal incision, pulled out, and surgically removed. In the Sham control group, tissues were pulled out but left intact and repositioned inside the peritoneal cavity. The above mice were allowed to recover for 1 week prior to any phenotypic characterization. Mice were excluded from the study if blood vessel injury or excessive bleeding was observed during surgery.

Citrobacter rodentium infection in the iFR mice. *Citrobacter rodentium* (*C. rodentium,* ATCC51459) infection involving iFR mice was maintained in a pathogen-free environment at Shenzhen Research Institute, Hong Kong Polytechnic University. The experiment was conducted following previously described protocols (1, 2). Briefly, iFR mice were weighed, fasted for 16 hours, and then were orally administered $1x10^9$ CFU of *C. rodentium* in PBS. Over a 2-week period, mice were monitored for weight changes,

and fecal *C. rodentium* CFU levels. After 2 weeks of infection, an equal amount of feces was mixed with PBS buffer. The homogenized samples were then plated onto MacConkey Agar (Millipore, M7408) and incubated at 37°C for 12-16 hours. The concentration of *C. rodentium* in the feces was calculated based on the CFU count.

Antibiotics (ABX) treatment. Mice were administrated with an antibiotic mixture (0.5 g/L ampicillin [Cat#14417, Cayman Chemical Company], 0.5 g/L neomycin [Cat#21810031, Thermo Fisher Scientific], 0.5 g/L metronidazole [Cat#9002409, Cayman Chemical Company] and 0.5 g/L vancomycin [Cat#15327, Cayman Chemical Company]) in drinking water for the 3 or 4 weeks as previously described (3). The drinking water with antibiotics was refreshed every 5 days.

SFB *colonization in iFR mice.* Twelve-week-old male C57BL/6J with iWAT removal or sham operation were treated with an ABX, as described above, for 3 weeks to deplete intestinal SFB or PBS as a vehicle control. iSham-ABX mice and iFR-ABX mice were then gavaged twice at a 24 hour-interval with 0.5 ml of fresh fecal homogenate from SFB monoassociated mice (kindly provided by Immune Regulation Research Laboratory, Yakult Central Institute, Tokyo, Japan) as previously described (4). Mice fecal DNA was isolated for the determination of SFB colonization by QPCR analysis.

Treatment with iWAT-EVs in animal models. (1) Young male C57BL/6J mice (2-3-month-old) and aged male C57BL/6J mice (20-24-month-old) or (2) iFR and their iSham mice or (3) iFR and their iSham mice with ABX treatment (iFR-ABX and iSham-ABX) were intraperitoneally injected with iWAT-EVs (100 μg total protein per injection) or iWAT-EVsΔRA (100 μg total protein per injection) every four days (iWAT removal mouse model) or every other day (aging mouse model), respectively, described in the figure legend. Isolation and purification of iWAT-EVs and iWAT-EVsΔRA are described in the subsequent sections.

Generation and administration of AAV-GFP and AAV-Cyp26c1. AAV-Rec2-*adiponectin-Cyp26c1* or AAV-Rec2-*adiponectin-GFP* were generated by Shandong Wei Zhen Bioscience Inc. The mouse *Cyp26c1* coding region (Gen ID: NM_001105201.1, size: 1557bp) and GFP were cloned into the AAV vector with the mouse *adiponectin* promoter. HEK293 cells were co-transfected with the AAV-*adiponectin-Cyp26c1* or AAV-*adiponectin-GFP* vector together with AAV serotype Rec2 plasmid and a helper-free system, followed by viral particles purification using iodixanol gradient method. The titer of the AAV particles was measured by qPCR analysis.

Twelve-week-old male C57BL/6J mice were locally injected with AAV-Rec2-*adiponectin-Cyp26c1* or AAV-Rec2-*adiponectin*-GFP at a dose of 1×10^{11} viral genomes per iWAT using a 50 μl microliter syringes. Four weeks post AAV injection, mice were sacrificed for tissue collection and biochemical studies.

Isolation of cells from gut lamina propria, Peyer's Patches, mesenteric lymph nodes (MLNs), and spleen. Tissue pieces from the ileum were initially incubated with an extraction buffer consisting of 5 ml RPMI 1640, 15 µl 5% [w/v] dithiothreitol (DTT), 10 µl 0.5 M EDTA, and 100 µl fetal bovine serum (FBS) at 37°C for 20 minutes to remove epithelial cells. Subsequently, the tissues were minced into small pieces and subjected to enzymatic digestion in a buffer containing 5 ml RPMI 1640, 2.5 mg dispase, 7.5 mg collagenase II, and 60 µl FBS. This mixture was incubated at 37°C for 30 minutes with shaking. The resultant cell suspension was passed through a 40-µm cell strainer (B.D. Biosciences) to obtain lamina propria lymphocytes (LPL). The cells from PPs and MLNs were isolated using the same digestion protocol as described above, except the initial epithelial cell removal step was omitted. Splenic cells were isolated by mechanically disrupting the tissue into a single-cell suspension using frosted glass slides. All the isolated cells were centrifuged at 500 g for 5 minutes and subsequently resuspended in Flow Cytometry Staining Buffer (5 mM HEPES, 2 mM EDTA, 1% BSA in PBS) for flow cytometric analysis or in RPMI 1640 culture medium for in vitro experiments.

Flow cytometric analysis. The single cell suspension was blocked with 5% rat serum or Fc receptor block (Anti-CD16/CD32, Cat#553141) for 20 minutes at 4°C, followed by staining with a cocktail of fluorophore-conjugated antibodies for 30 minutes at 4°C

in the dark. The surface antibodies used included: anti-CD45 (Pacific Blue, Cat# B240806/APC-CyTM7, Cat#557659), anti-B220 (PE-Cy7, Cat# 552772/BV421, Cat#562922), anti-CD3 (FITC, Cat#561798), anti-CD4 (APC-H7, Cat# 560181/560246); anti-PD-1 (BB700, Cat# 566515), anti-CD21 (PE-CF594, Cat# 563959), anti-CD27 (Percp-Cy5.5, Cat#563603), anti-CXCR5 (PE, Cat# 551959); anti-CD14 (APC, Cat# 560634), anti-CD5 (BV421, Cat#562739), anti-CD103 (BV510, Cat# 748258), anti-CD11b (BV605, Cat# 563015), anti-CD11c (PerCP-Cy5.5, Cat#7187525), anti-GL-7 (PE, Cat#561530) and anti-IgD (PerCP-Cy5.5, Cat# 564273).

After the cell surface staining, the cells were washed twice with FACS buffer. For intracellular staining, the Transcription Factor Staining Buffer Set (eBioscience, Cat# 562574) was used according to the manufacturer's instructions. Intracellular antibodies included: anti-IL-17A (Alexa Fluor 647, Cat# 560224), anti-RoRγt (PerCP-Cy5.5, Cat# 562683), anti-Foxp3 (PE, Cat# 560414), anti-IFNγ (FITC, Cat# 562019); anti-IgA (FITC/BV605, Cat# 559354/743295), anti-IgM (APC/PE, Cat# 406509/553409), anti-IgG1 (APC-Cy7/R718, Cat# 406619/752272) and TGF-b1 (PE, Cat#141403). Flow cytometric analysis was conducted using a BD FACS Aria III Cell Sorter (B.D. Biosciences), and data were analyzed using FlowJo software.

Quantification of IgA-coated bacteria in the ileum. Fecal contents from the ileum were flushed with sterile PBS, followed by vortexing and centrifugation at 300 g for 5 min to remove large particles. The supernatant was collected, and bacteria were harvested by centrifugation at 8,000 g for 5 min at 4°C. Bacterial pellets were fixed in 2%

paraformaldehyde for 15 min, and then blocked with 5% normal rat serum for 10 min. Bacteria were stained with SYTO® BC green fluorescent dye (5 mM solution in DMSO, ThermoFisher Scientific, Cat# S34855) for 20 min. After washing twice with PBS, bacteria were stained with an anti-IgA antibody on ice for 30 min.

Quantification of immunoglobulin-secreting cells (Ig-SC). SFB lysates were prepared from the feces of SFB-monoassociated mice by centrifugation through a Nycodenz gradient (Sigma, Cat#D2158) and Ig-SC from LPL were isolated (described above) and assessed in the enzyme-linked immunospot (ELISpot) assay using a multiscreen HTS 96-well plates (Millipore, Cat# MSHVN4B10) as described (4). Briefly, multiscreen HTS 96-well plates were coated with 100 μl/well of goat anti-mouse Ig (H+L) (Invitrogen; Cat#88-50450-22) or bacterial sonicate (40 μg/ml) overnight at 4℃ to determine total and bacterium-specific Ig-SCs, respectively. Nonspecific sites were blocked with 5% FBS for 3 hours at 37 ℃, and 10^6 cells of LPL were added and incubated overnight at 37℃ and 5% CO_2. Ig-SC were revealed at room temperature by a 2 hours incubation with HRP-conjugated anti-mouse IgA (Invitrogen; Cat#88-50450-22), and 15-30 min incubation with 100 μl /well AEC Substrate Kit (BD, Cat#551015). Ig-SC were analyzed with a stereomicroscope (AID EliSpot Reader System) and AID EliSpot 5.0 software (Autoimmun Diagnostika GMBH).

Differentiation of 3T3-L1 cells into mature adipocytes. 3T3-L1 cells were cultured with Dulbecco's Modified Eagle Medium (DMEM, ThermoFisher, Cat# 12800082) supplemented with 10% fetal bovine serum (FBS, ThermoFisher, Cat# 10270) and 1% penicillin-streptomycin (ThermoFisher, Cat# 15140122). Upon reaching 100% confluence, the cells were maintained for an additional 48 hours to ensure growth arrest. The growth medium was then replaced with a differentiation medium containing DMEM supplemented with 10 μg/ml insulin, 0.25 μM dexamethasone, 0.5 mM 3-isobutyl-1-methylxanthine (IBMX), and 100 nM rosiglitazone. The differentiation medium was replenished every two days. On day 4, the medium was switched to a maintenance medium consisting of DMEM supplemented with 1 μg/ml insulin. This maintenance medium was also changed every two days for an additional four days. The extent of adipocyte differentiation was assessed using phase contrast microscopy, with or without Oil Red O staining to visualize lipid accumulation.

Collection of conditioned medium (CM). Collection of conditioned media (CM) from different sources: (1) From iWAT: iWAT (100 mg) from 10-14-week-old C57BL/6J mice were minced and cultured in 1 ml DMEM containing 10% FBS EVs-free at 37°C for 24 hours. (2) From adipocytes and stromal vascular fraction (SVF) of iWAT: iWAT from 10-14-week-old C57BL/6J mice were minced and digested in a digestion buffer (HBSS, 1 mg/ml collagenase type II, 10 mM HEPES, and 1% FBS, pH 7.0) at 37°C for 40 minutes. The digested cell mixture was filtered through a 100-μm cell strainer and centrifuged at 500 g for 5 min to separate mature adipocytes from the SVF. Both

fractions were cultured in RPMI 1640 medium (for B cell culture) or DMEM (for EV collection) with 10% FBS EVs-free at 37°C for 24 hours. (3) From 3T3-L1 adipocytes: Differentiated 3T3-L1 adipocytes were cultured with RPMI1640 medium with 10% FBS EVs-free at 37°C for 24 hours.

Cell debris was removed from the CM by centrifugation at 500g for 10 min and then filtered through a 0.22-µm filter.

Generation of CM-EVs, CM-EVs without RA, CM without EVs, and serum EVs. EVs were depleted in the CM by ultracentrifugation at 100,000 g for 6 hours at 4°C. The resulting supernatant was collected and labeled as CM with EVs depleted. The pellet containing EVs was washed with PBS twice and then resuspended in RPMI1640 for cell culture experiments or in PBS for animal studies. To inhibit EV biogenesis, iWAT was treated with 10 µM GW4869 (R&D Systems, cat#6741) for 48 hours. The CM was then collected, filtered through a 100-µm cell strainer, and centrifuged at 500 g for 5 min to remove cell debris.

To obtain adipocyte-EVs or iWAT-EVs without RA, 3T3-L1 adipocytes and the minced iWAT were cultured in RPMI1640 medium or DMEM containing *10% FBS EVs-free* in the presence of WIN18446 (10 µM; R&D Systems, Cat# 4736) for 48 hours. The CM was then collected, followed by the removal of cell debris through ultracentrifugation and filtration as described above.

Serum EVs were isolated using the miRCURY Exosome Serum Isolation Kit (Qiagen, Cat# 76603) according to the manufacturer's instructions. Briefly, 0.5 ml

serum was incubated with Precipitation Buffer A overnight, followed by centrifugation at 1500 g for 30 min at 20°C to collect the serum EVs.

Characterization of iWAT-EVs. The morphology of iWAT-EVs was visualized through transmission electron microscopy (JEOL Model JEM-2010, PolyU). Briefly, 5 µl of the iWAT-EVs (2 µg/µl, protein level) was fixed in 4% paraformaldehyde overnight. The fixed iWAT-EVs solution was then placed on Formvar-carbon coated (200 Mesh, Cu) electron microscopy grids for 15 min incubation, then washed with distilled water and stained with 2% uranyl acetate before visualization.

The different sizes of iWAT-EVs were isolated from iWAT-CM by ultracentrifugation through g-forces of 2k (apoptotic body), 15k (MV), 33k (EV-33k), and 100k (EV-100k) as described (5). The size distribution of iWAT-EVs were then measured by NanoSight (Malvern, U.K., NS300HSBF). Briefly, the samples were diluted in PBS to a final volume of 1 ml. In the setup, the camera level was adjusted until all particles were visible, ensuring that the particle signal saturation remained below 20%. The optimal detection threshold was established to encompass as many particles as possible with the requirement that 10–100 red crosses were counted while ensuring that less than 10% were not associated with distinct particles. The Zeta potentials of iWAT-EVs were determined using a ZetaView® PMX 420 QUATT laser instrument.

EV tracking analysis. In vitro uptake of EVs by unswitched B cells: iWAT-EVs were pre-stained with PKH26 using a PKH26 Red Fluorescent Cell Linker Kit (Sigma, Cat# MINI26-1KT) according to the manufacturer's instructions. Splenic unswitched B cells were cultured in RPMI1640 medium containing PKH26-labeled iWAT-EVs (20 μg/ml) for 24 hours. The uptake of EVs was visualized using a fluorescence microscope (NIKON Eclipse Ni-U).

In vivo tracking of iWAT-EVs labeled with DiR: iWAT-EVs were labeled with DiR (Invitrogen, Cat# D12731) at a ratio of 500:1 (v/v) for 30 minutes. Subsequently, 100 μg of DiR-labeled iWAT-EVs were intraperitoneally or tail vein injected into C57BL/6J mice. After 24 hours, the distribution of DiR-labeled iWAT-EVs within the animals was detected using an IVIS Lumina II in vivo imaging system (PerkinElmer, ThermoFisher, US) or through tissue sectioning for visualization.

In vivo tracking of DC271-encapsulated iWAT-EVs (iWAT-EVs (DC271)): iWAT-EVs were incubated with 1 mg/ml DC271 (R&D Systems, Cat#6873) at a 1:1 (v/v) ratio for 24 hours. Subsequently, the quantity of DC271-encapsulated iWAT-EVs was assessed using a Fluorescent Microplate Reader (Thermo Fisher) with excitation at 350nm and emission at 540nm. Next, 100 μg of DC271-labeled iWAT-EVs were intravenously vein injected into C57BL/6J mice. After 24 hours, the fluorescent signal in the intestine was analyzed using flow cytometry or visualized through tissue section under a fluorescent microscope (Nikon Eclipse Ni-U Fluorescent Microscope).

Isolation and treatment of unswitched B cells. To isolate unswitched B cells, single-cell suspensions were prepared from the spleens of 10-12-week-old C57BL/6J mice. B cells were isolated by negative selection using the EasySep™ Mouse B Cell Isolation Kit (STEMCELL Technologies, Cat# 19854) according to the manufacturer's instructions. Non-B cells were removed by incubation with biotinylated antibodies recognizing non-B cells. The enriched B cell suspension was stained with antibodies against B220, IgM, IgD, and IgA, followed by sorting of $B220^{+}IgM^{+}IgD^{+}IgA^{-}$ unswitched B cells using a BD FACS Aria III Cell Sorter. To examine the effects of the CM or EVs on IgA class-switching and B cell differentiation, $1x10^5$ unswitched B cells were incubated with the CM or RPMI1640 containing EVs (10 μg total protein/ ml) for six days. Before measurement of IgA in the culture medium on day 6, the treated cells were washed with PBS, and fresh medium was added on day 5. On day 6, the B cells were subjected to flow cytometric analysis of IgA^{+} cell populations as described above. To assess if pharmacological inhibition of the RA receptor (RAR) α blocks the effectiveness of iWAT-CM or iWAT-EVs on IgA class-switching and B cell differentiation, unswitched B cells were incubated with 10 μg/ml BMS195614 (RAR α inhibitor; R&D Systems, Cat# 3660).

Measurement of enzymatic activity of ALDH in dendritic cells. Aldehyde dehydrogenases (ALDH) activity in $CD45^{+}CD11c^{+}$ gated $CD11b^{+}CD103^{+}$ LP dendritic cells (LPDC) was evaluated using the ALDEFLUOR assay (STEMCELL™ Technologies, Cat#01700) according to the manufacturer’s protocol with some

modifications. Briefly, immune cells isolated from the LP were stained with anti-CD45, anti-CD11c, anti-CD11b, and anti-CD103 antibodies. The stained cells were resuspended in 0.5 ml assay buffer containing 5 μl of ALDEFLUOR reagent or 10 μl of the ALDH inhibitor (DEAB) for 45 min in the dark at 37°C. $ALDH^+$ LPDC were identified as cells with brighter fluorescent intensity than the DEAB-inhibited sample that was processed in parallel.

Real-time quantitative PCR (QPCR) and semi-quantitative PCR. Total RNA from tissues or cells was extracted using TRIzol Reagent (Invitrogen, Cat# 15596026; Takara, Cat# 9108). One μg RNA was reverse transcribed to cDNA using Superscript III reverse transcriptase and random hexamers (Promega, Cat# A5001). The cDNA or bacterial DNA were diluted and mixed with SYBR Green PCR master mix (QIAGEN, Cat# 208052) and specific primers (see Key Resources Table), followed by real-time qPCR analysis using a ViiA7 Real-Time PCR System (Applied Biosystems). Gene expression levels were normalized with housekeeping genes and expressed as fold change over the control as indicated in the Figure legends. For semi-quantitative PCR, cDNA was prepared from B cells or tissues as described above. Amplification of αCT transcripts (product sizes: 317 bp and 171 bp) was performed using the primers Ia *253-F*, Cμ *94-R*, Iα *254-F*, Cμ *93-R*. Amplification of αGT transcripts (product sizes: 497 bp, 357 bp, and 95 bp) was performed using the primers IαF and CαR. Amplification of Iμ-Cα transcripts (product size: 267 bp) was performed using the primers ImF and CαR. Hprt was used as a housekeeping control (product size: 131 bp). The transcripts

were amplified for 30 cycles, followed by DNA gel electrophoresis. Primer sequences can be found in the Key Resources Table.

Western blot analysis. The tissues and cells were solubilized in a RIPA buffer (NaCl [150 mmol/L], deoxycholate [1%], EDTA [1 mmol/L], Triton X-100 [1%], sodium dodecyl sulfate [0.1%] in Tris-HCl [50 mmol/L], pH 7.4) with protease and phosphatase inhibitors cocktail (Bimake, Cat# B15001/B15002). An equal amount of total lysate was loaded and separated by an SDS-PAGE, followed by wet transfer to a PVDF membrane and blocking with 5% non-fat milk. The membranes were incubated with the primary antibodies overnight at 4 °C as indicated in the Figure legends. After washing with TBS-T (20 mM Tris base+150 mM NaCl +0.1% Tween 20), the membranes were incubated with the secondary antibodies conjugated with HRP, followed by washing with TBS-T four times and incubation with the chemiluminescence reagents (Thermofisher, Cat# 32109). The signals of protein abundance were captured by the X-ray films and quantified by ImageJ software. The list of antibodies used in this study can be found in the Key Resources Table.

Gut permeability assay. Four hours before the assay, mice were fasted and then orally gavaged with fluorescein isothiocyanate–dextran 4000 (DX-4000-FITC, Sigma, Cat# 46944) at a dosage of 500 mg/kg body weight. Serum samples were collected at baseline (0 hour) and at 1- and 3-hour post-gavage. The serum was diluted with PBS at a ratio of 1:10 (v/v), and FITC signals were detected using a ThermoFisher Scientific

Varioskan LUX multimode microplate reader with an excitation wavelength of 485 nm and an emission wavelength of 535 nm. Standard curves were constructed by serial dilution of DX-4000-FITC.

Measurement of biochemical parameters. Commercial assay kits were used to determine the levels of serum IgA (Invitrogen; Cat#88-50450-22), IgM (Invitrogen, Cat#88-50470-22), IgG (Invitrogen, Cat#88-50400-88), IL17A (Invitrogen, BMS6001), ABAFF (R&D System, Cat# MBLYS0), TGF-b1 (R&D System, Cat# DB100B), Adiponectin (Antibody and Immunoassay Services, HKU, Cat#32010), Leptin (BioVender, Cat#291001200R), Triglycerides (TG) (STABIO Laboratory, Cat#2100-430), alanine transaminase (ALT) (STABIO Laboratory, Cat#204111), aspartate aminotransferase (AST) (STABIO Laboratory, Cat#198719), and lipopolysaccharides (LPS) (Hycult Biotech, Cat#HIT302), following the manufacturer's instructions. For fecal IgA and IgM measurements, 50 mg of fresh feces were homogenized and diluted at 1:3000 (v/v) with 1x Assay Buffer from the kits.

Untargeted metabolomics analysis. 100 µl serum sample was spiked with 4-chloro-phenylalanine and ethyl gallate as the internal standards at a final concentration of 100 ng/ml. The mixture was then extracted with ice-cold 100% methanol (1 ml), centrifuged at 17,000 g at 4 °C for 15 min, and dried using a SpeedVac concentrator. The dried sample was reconstituted in 80% methanol (50 µl) followed by centrifugation at 17,000 g for 10 minutes. The collected supernatant was then analyzed with the

Thermo Scientific UltiMate 3000 UHPLC system coupled with an Orbitrap IQ-X Tribrid Mass Spectrometer (Thermo Scientific, San Jose, CA). Chromatographic separation was achieved with a Waters ACQUITY HSS T3 column (2.1 mm × 100 mm, 1.7 μm) (Milford, MA) maintained at 25 °C. The binary mobile phase consisted of (A) 0.1% formic acid in water and (B) 0.1% formic acid in acetonitrile, and the flow rate was set to 0.3 ml/min. The gradient elution (B%) started at 2% from 0 to 1 min; 35% at 5 min; 99% at 10 min and was then held until 12 min. The column was equilibrated for 3 min before the next injection. The injection volume was 5 μl. The mass scan range was 70−1200 m/z with data-dependent acquisition by the AcquireX workflow under both positive and negative electrospray ionization (ESI) modes. Data processing and metabolite identification were performed using Thermo Scientific Compound Discoverer 3.3 with mzCloud, Human Metabolome Database (HMDB), and ChemSpider databases.

Measurement of RA. To extract all-trans RA (at-RA), the tissues or serum samples were mixed with 300 μl of ammonium acetate (5 mM) in PBS and 20 μl of the internal standard at-RA-d5 (20 ng/ml) (Santa Cruz, SC-217605) were mixed. The samples were then vortexed and incubated with a 4 ml extraction solvent containing ethyl acetate: n-hexane: isopropyl alcohol = 30:65:5 (v/v/v). Phase separation was performed by centrifugation at 3000 rpm for 5 minutes. The organic layer (upper layer) was transferred to a new glass tube and evaporated under nitrogen flow. The samples were then reconstituted in 200 μl of 5 mM ammonium acetate buffer: methanol: acetonitrile

= 30:30:40 (v/v/v) before LC-MS/MS analysis as previously described (6, 7). Quantification of RA was performed using a SCIEX ExionLC UHPLC-SCIEX 6500+ QTRAP LC/MS System equipped with an ion-mobility (I.M.) device (SelexION Technology). Chromatographic separation was carried out with a Waters ACQUITY UPLC BEH C18 1.7 μm column (2.1x100 mm) with solvent A as 5 mM of ammonium acetate in H_2O: methanol = 60:40 (v/v) and solvent B as 5mM of ammonium acetate in H_2O: ACN = 10:90 (v/v). The solvent gradient was 0-2 min: 40% B; 2-10 min: 40%-95% B, 10-11 min: 95% B, 11-11.5 min: 95%-40% B, 11.5-14.5 min: 40% B. The solvent flow rate was 0.4 ml/min and the injection volume was 20 μL. The mass spectrometer was operated in multiple-reaction monitoring (MRM) mode with MRM transitions of m/z 299.1 ($[M\text{-}H]^-$) → 255.3 for at-RA and m/z 304.0 ($[M\text{-}H]^-$) → 260.2 for at-RA-d5. The instrumental parameter settings for both MRM transitions were: Ionization mode: negative ESI, Declustering potential (DP): -90 V, Entrance potential (EP): -10 V, Collision energy (CE): -22 V, Collision cell exit potential (CXP): -20 V, IM Separation voltage (SV): 4000 V, IM Compensation voltage (COV): -24 V. The ion source parameters were: Ionspray voltage: -3,800 V, Source temperature: 600 ºC, Curtain gas: 20 a.u, Gas 1: 60 a.u, and Gas 2: 60 a.u. The modifier for I.M. separation was 2-propanol.

Proteomics analysis. Sample preparation: 50 μg proteins from iWAT-EVs samples were digested with Trypsin/Lys-C Protease Mix from EasyPep™ Mini MS Sample Prep Kit (Thermo, #A40006). Peptide samples were dried using a vacuum centrifuge

(Refrigerated CentriVap Centrifugal Concentrator and CentriVap Cold Traps) at 4°C. The peptide segments powder was dissolved in 100 µl 0.1% formic acid water solution for LC-MS/MS loading. LC-MS/MS analysis: The tryptic digests (0.5 µg/µL) were injected into an UltiMate 3000 RSLCnano system (Thermo Scientific, USA) using a trap-and-elute method. For trapping, a PepMap C18 column (5 mm × 300 µm, Thermo Scientific, USA) was used, followed by elution through an Aurora C18 analytical column (25 cm × 75 µm, IonOpticks, Australia) equipped with a CSI emitter. Trapping was performed at a flow rate of 50 µL/min for 2 min, and the elution was carried out using a gradient of solvent A (0.1% formic acid in water) and solvent B (0.1% formic acid in acetonitrile). The gradient was as follows: 2% B for 0–2 min, 5–35% B from 2–47 min, 35–90% B from 47–51 min, 90% B from 51–56 min, and re-equilibration at 2% B from 56–60 min. The flow rate was maintained at 300 nL/min, with all trapping and elution steps performed at 50°C. Eluted peptides were introduced into a timsTOF Pro 2 mass spectrometer (Bruker Daltonics, USA) using the following source settings: capillary voltage of 1400 V, dry gas flow rate of 3.0 L/min, and dry temperature of 180°C. The trapped ion mobility spectrometry (TIMS) was set to a range of 1/K0 values from 0.6 to 1.6 V·s/cm^2, with a ramp time of 100 ms. Data acquisition was performed in DIA-PASEF mode with a resolution of 60,000 at m/z 1,200 and a scan rate of over 120 Hz. The Direct-DIA spectra from iWAT-EVs samples were analyzed using Spectronaut 19 software. Peptide and protein identifications were searched against the UniProt mouse protein database. The results were filtered based on a Q-value cutoff of 0.01, corresponding to a false discovery rate (FDR) of less than 1%. Proteins with fold

change greater than 1.2 or less than 0.8, with a p-value<0.05, were considered differentially expressed.

Bacterial DNA extraction, 16s rRNA sequencing, metagenomics sequencing and analysis. Bacterial DNA from mouse cecum was extracted using the PSP Spin Stool DNA Plus Kit (STRATEC MOLECULAR, Cat# 1038100200) according to the manufacturer's instructions. Briefly, 100 mg feces from the mouse cecum were vigorously vortexed and mixed with the stabilizer liquid in the collection tubes. The extracted DNA was eluted in 100 μl nuclease-free water and quantified using a Nanodrop 2000 Spectrophotometer (Thermo Scientific).

The 16S rRNA sequencing and data analysis in the iWAT removal C57BL/6J mouse strain (Figure 1) were performed by Groken Bioscience Ltd. The V3-V4 hypervariable regions of 16S rRNA genes were amplified using a universal forward sequencing primer and a uniquely barcoded reverse sequencing primer to allow multiplexing. Amplicon sequencing was conducted on the Illumina HiSeq platform, generating 250 bp paired-end raw reads. Raw data were first merged and filtered to obtain clean data. Chimeric sequences were detected and removed to obtain effective data. Effective data were grouped by 97% DNA sequence similarity into operational taxonomic units (OTUs), with low abundance OTUs (<0.005%) removed. OTU clustering and species annotation were performed, and the relative species evenness and abundance distribution were analyzed. OTU picking, taxonomic assignment, and construction of phylogenetic trees were followed by downstream statistical analysis to

explain differences in community composition between samples or groups via Principal Component Analysis (PCA).

The 16S rRNA sequencing and data analysis in the iWAT removal BALB/c mouse strain (Supplementary Figure 7 and 8) and metagenomics analysis in the AAV-*Cyp26c1* mouse model (Supplementary Figure 22 and Supplementary Table 5) were performed by Lian Chuan Bio Company using NovaSeq 6000. Raw data were processed using fastp (parameters: -l 100 -g -W 6 -5 -q 20 -u 30) to obtain high-quality clean data. Preprocessing steps included: (1) removal of adapter sequences, (2) quality scanning with a sliding window approach (6 bp window size, trimming if average quality < 20), (3) removal of sequences < 100 bp post-trimming, and (4) removal of host sequence contamination. Valid data were assembled using MEGAHIT, employing a multi-kmer size strategy to construct succinct de Bruijn graphs iteratively, resulting in contigs. Contigs > 500 bp were retained for clustering analysis. Metaprodigal was used to predict coding regions (CDS) from contigs, filtering out sequences < 100 nt. MMseqs2 software was used for redundancy reduction, yielding a non-redundant set of unigenes. Bowtie2 aligned clean data to unigene sequences, calculating read counts for each unigene. Unigenes with read counts ≤2 across all samples were filtered out, leaving the final set for subsequent analysis. Low abundance OTUs (<0.001%) were removed. The relative abundance at phylum, family, genus, and species levels was further analyzed.

Metagenomic sequencing in the aging mouse model with the treatments (Supplementary Figure 27) was performed by the Beijing Genomics Institute (BGI).

Briefly, purified PCR products of fecal bacterial DNA were fragmented, end-repaired, adaptor-ligated, and sequenced on the DNBseq platform using paired-end reads of 150 bp. The PhiX control was added to the sequencing library. Raw reads were first trimmed and quality-checked using TrimGalore v0.5.0. Reads were classified by Kraken2/Bracken to determine bacterial taxonomic compositions. Low abundance OTUs (<0.001%) were removed. The relative abundance at phylum, family, genus, and species levels was analyzed. OTU picking, taxonomic assignment, and construction of phylogenetic trees were followed by downstream statistical analysis to explain differences in community composition between samples or groups via Principal Component Analysis (PCA).

Histological and immunobiological analysis. Tissues were fixed in 10% formalin (ThermoFisher, Cat# 6764240) for two days, followed by dehydration, clearing, and wax infiltration using a tissue processor (ThermoFisher). The tissues were then embedded in paraffin and sectioned at 4 μm. The sections were deparaffinized, rehydrated, and then subjected to H&E staining, alcian blue staining, immunohistochemistry, or immunofluorescent staining. Assessment of intestinal inflammatory score was conducted using established scoring rubrics in mouse models to evaluate intestinal inflammation(8). Antigen retrieval was performed by boiling in either Tris-EDTA (pH 9.0) or citric buffer (pH 6.0) for 20 minutes. Sections were blocked with PBS containing 3% FBS and incubated with various primary antibodies in PBS-T (PBS, 0.1% Triton-X 100) and corresponding secondary antibodies or

fluorophore-conjugated antibodies (see Key Resources Table). For immunohistochemistry, sections were further incubated with DAB peroxide substrate (Sigma, Cat# D4418), and for immunofluorescent staining, sections were mounted with DAPI (Invitrogen, Cat# MAN0017262).

Intestinal tissue specimens were carefully positioned to showcase longitudinally sectioned crypts, enabling a meticulous evaluation of changes in the overall architecture of the intestinal tissue. In the case of small intestine samples, a minimum of 4-5 successive well-extended villi, from base to tip, were evaluated. Unless specified otherwise, the Figures include representative images depicting longitudinal sections. The assessment of inflammatory score is detailed in the Supplementary Figure 28.

The intestinal tissues were embedded in OCT for IgA and IgM immunofluorescent staining. Briefly, intestinal tissues were positioned to longitudinally sectioned crypts and placed into OCT gel, stored at -20 degrees, followed by sectioned at 10 μm. The cryostat sections were fixed by 4% PFA and washed in PBS three times, and then blocked in 5% BSA in PBST (0.5% TritonX-100) for 30 min. The intestine sections were incubated with anti-IgA and IgM antibodies overnight at 4°C. In co-immunofluorescent staining, sections were mounted with DAPI and visualized by Nikon Eclipse Ni-U Fluorescent Microscope.

3T3-L1 mature adipocytes were fixed with 10% formalin for 30 min, followed by staining with Oil Red O for 10 min. After washing with distilled water, visible lipid droplets were captured using light microscopy.

Data analysis. All data are presented as mean ± standard error of the mean (s.e.m.). Each data point derived from qPCR assays represents an average of at least two technical replicates. Normality and equality of variances were tested using the Kolmogorov-Smirnov test and Levene's Test, respectively. Statistical significance was determined using a two-tailed Student's t-test for data that were normally distributed (Kolmogorov-Smirnov test, p-value > 0.05) and had equal variance (Levene's Test, p-value > 0.05). For data that were not normally distributed or unequal variance, a nonparametric two-tailed Mann-Whitney U test was performed. One-way or Two-way analysis of variance (ANOVA) with Tukey's multiple comparisons test was used for multiple comparisons. Statistically significant differences are indicated by actual value. All statistical analyses were performed either using Prism 8.0 (GraphPad Software) or SPSS.

Materials

Key Resources Table

Reagent and Resource	Source	Identifier
**Antibodies**		
Anti-B220 (PE-Cy7)	B.D. Bioscience (PharMingen)	Cat#552772
Anti-CD45 (Pacific Blue/APC-Cy7)	BioLegend	Cat#B240806
Anti-CD27 (Per-cy5.5)	B.D. Bioscience (PharMingen)	Cat#563603
Anti-CD138 (APC/PE)	B.D. Bioscience (PharMingen)	Cat#5586261/561070
Anti-CD16/CD32	B.D. Bioscience (PharMingen)	Cat#553141
Anti-CD103 (BV510)	B.D. Bioscience (PharMingen)	Cat#748258
Anti-GL-7 (P.E.)	B.D. Bioscience (PharMingen)	Cat#561530
Anti-CD4 (APC-H7)	B.D. Bioscience (PharMingen)	Cat#560181/560246
Anti-TGF-b1 (PE)	BioLegend	Cat#141403
Anti-CD21(PE-CF594)	B.D. Bioscience (PharMingen)	Cat#563959
Anti-CD11b (BV605)	B.D. Bioscience (PharMingen)	*Cat#563015*
Anti-CD11C (Percp-Cy5.5)	B.D. Bioscience (PharMingen)	Cat#7187525
Anti-CXCR5 (P.E.)	B.D. Bioscience (PharMingen)	Cat#551959
Anti-PD-1(BB700)	B.D. Bioscience (PharMingen)	Cat#566515
Anti-CD14 (APC)	B.D. Bioscience (PharMingen)	Cat#560634
Anti-IgD (Percp-Cy5.5)	B.D. Bioscience (PharMingen)	Cat#564273
Anti-IL-17A (APC)	B.D. Bioscience (PharMingen)	Cat#560224
Anti-RoRrt (Percp-Cy5.5)	BD Bioscience (PharMingen)	Cat#562683
Anti-Foxp3 (P.E.)	B.D. Bioscience (PharMingen)	Cat#560414
Anti-IFNγ (FITC)	B.D. Bioscience (PharMingen)	Cat#562019
Anti-IgA (FITC/BV605)	B.D. Bioscience (PharMingen)	Cat#559354/743295
Anti-IgM (APC/ PE)	BioLegend/ B.D. Bioscience (PharMingen)	Cat#406509/550676
Anti-IgM (PE)	B.D. Bioscience (PharMingen)	Cat#553409
Anti-IgG1 (APC-Cy7)/(BV421)	BD Bioscience (PharMingen)/ Biolengend	Cat#406619/562580
Anti-CD5 (BV421)	B.D. Bioscience (PharMingen)	Cat#562739
Anti-GAPDH	Cell Signaling Technology	Cat#5174
Anti-Adiponectin	Immuno Diagnostics Limited	Cat#12010
Anti-AID	Invitrogen	Cat#14-5959-82
Anti-ZO-1	Invitrogen	Cat#61-7300
Anti-CD63	Abcam company	Cat#ab217345
Anti-Cleaved Caspase 3	Cell Signaling Technology	Cat#9661S
Anti-HSP70	Abcam company	Cat#ab2787
Anti-RALDH2	Abcam company	Cat#ab75674
Anti-CYP26C1	Invitrogen	Cat#PA5-106558

Anti-HSP90	Cell Signaling Technology	Cat#4877
HRP-Conjugated Anti-Rat Antibody	Cell Signaling Technology	Cat#7077S
HRP-Conjugated Anti-Mouse Antibody	Cell Signaling Technology	Cat#7076S
HRP-Conjugated Anti-Rabbit Antibody	Cell Signaling Technology	Cat#7074S
**Chemicals and Recombinant Proteins**		
RPMI1640	Gibco™	Cat#31800105
DMEM	Gibco™	Cat#12100061
Fetal Bovine Serum (FBS)	ThermoFisher Scientific	Cat#10270106
Penicillin- Streptomycin (P.S.)	ThermoFisher Scientific	Cat#15140122
Beta-Mercaptoethanol	Bio-Rad Laboratories, Inc.	Cat#1610710
Trypsin	Gibco™	Cat#25200056
Bovine Serum Albumin (BSA)	Sigma-Aldrich	Cat#A7906
MacConkey Agar	Millipore	Cat#M7408
EDTA	Sigma-Aldrich	Cat#EDS-100G
Dispase	Gibco™	Cat#17105041
Collagenase II	Gibco™	Cat#17101015
GW4869	R&D Systems	Cat#6741
WIN18446	R&D Systems	Cat# 4736
BMS195614	R&D Systems	Cat# 3660
HEPS	Sigma-Aldrich	Cat#H3375
DC271	R&D Systems	Cat#6873
Dir	Invitrogen	Cat# D12731
SYBR Green PCR master mix	QIAGEN	Cat#208052
Triton X-100	Sigma-Aldrich	Cat#T8787
Nycodenz	Sigma-Aldrich	Cat#D2158
Tris	Bio-Rad	Cat#161-0719
DX-4000-FITC	Sigma-Aldrich	Cat#46944
Glucose	FLUKA	Cat#49138
Human Insulin	Sigma-Aldrich	Cat#9077-M
Formalin	ThermoFisher Scientific	Cat#6764240
Hematoxylin	Sigma-Aldrich	Cat#HHS32
Eosin	Sigma-Aldrich	Cat#318906
Histological Paraffin	ThermoFisher Scientific	Cat#8331
3,3’ Diaminobenzidine (DAB) Tablets	Sigma-Aldrich	Cat#D4418
AEC Substrate Kit	B.D. Bioscience	Cat#551015

Tween-20	Sigma-Aldrich	Cat#P2287
Absolute Ethanol	Sigma-Aldrich	Cat#459844
Xylene	Sigma-Aldrich	Cat#185566
DPX Mounting Medium	Sigma-Aldrich	Cat#06522
Prolong Class antifade mountant medium	Invitrogen™	Cat# MAN0017262
Acetic Acid	Sigma-Aldrich	Cat#320099
Methanol Solution	DUKSAN	Cat#62
Trizol RNA Isolation Reagent	Invitrogen™	Cat#15596026
Chloroform	Sigma-Aldrich	Cat#650498
Ketamine, 10%	Alfasan International B.V.	Cat#HK-37715
Xylazine, 2%	Alfasan International B.V.	Cat#HK-56179
Pageruler™ Prestained Protein Ladder	ThermoFisher Scientific	Cat#26617
Ampicillin	Cayman Chemical Company	Cat#14417
Neomycin	Thermo Fisher Scientific	Cat#21810031
Metronidazole	Cayman Chemical Company	Cat#9002409
Vancomycin	Cayman Chemical Company	Cat#15327
Acryl/Bis 30% Solution	Sangon Biotech Co., Ltd.	Cat#B546018-0500
Ammonium Persulfate	Sigma-Aldrich	Cat#A3678
TEMED	ThermoFisher Scientific	Cat#17919
Prolong™ Gold Antifade Mountant With (DAPI)	ThermoFisher Scientific	Cat#P36941
All-trans-RA-d5	Santa Cruz	SC-217605
Ammonium acetate	Sigma-Aldrich	Cat#238074
All-trans-RA	Cayman Company	Cat#11017
Ethyl acetate	Anaqua Company Limited	Cat#AL-1551-4000
n-hexane	Anaqua Company Limited	Cat#HX-1251-2500
Methanol	Anaqua Company Limited	Cat#MA-1296-4000
Acetonitrile	Anaqua Company Limited	Cat#AC-1026-4000
Isopropyl Alcohol	Anaqua Company Limited	Cat#IA-1281-4000
Critical Commercial Assays		
Easysep™ Mouse B Cell Isolation Kit	STEMCELL™ Technologies	Cat#19854

HTS 96-well plates	Millipore	Cat# MSHVN4B10
SYTO BC Kit	ThermoFisher Scientific	Cat#S34855
PSP Spin Stool DNA Plus Kit	Stractec Molecular GmbH	Cat#1038100x00
Mouse ELISA IgA Kit	Invitrogen™	Cat# 88-50450-22
Mouse ELISA IgM Kit	Invitrogen™	Cat# 88-50470-22
Mouse ELISA IgG Kit	Invitrogen™	Cat# 88-50400-22
Mouse ELISA IL17A Kit	Invitrogen™	Cat#BMS6001
Mouse BAFF ELISA kit	R&D System	Cat# MBLYS0
Mouse TGF-b1 ELISA kit	R&D System	Cat# DB100B
Mouse APRIL ELISA Kit	LSBIO	Cat#LS-F12794
BCA protein assay Kit	ThermoFisher Scientific	Cat#23225
PKH26 Red Fluorescent Cell Linker Kit	Sigma-Aldrich	Cat#PKH26GL
Mouse Triglycerides Detection Kit	STABIO Laboratory	Cat#2100-430
Mouse Leptin ELISA Kit	BioVender RD	Cat#291001200R
Mouse Adiponectin ELISA Kit	*Antibody and Immunoassay Services, HKU*	*Cat#32010*
Enzymes Aspartate Transaminase (AST)	STABIO Laboratory	Cat#2920-430
Alanine Transaminase (ALT)	STABIO Laboratory	Cat#2930-430
LPS ELISA Kit	Hycult Biotech	Cat#HIT302
Picro-Sirius Red Stain Kit	Abcam	Cat#ab246832
ECL Western Blot Detection Kit	Bio-Rad	Cat#1705060
Reverse Transcription Kit	Promega	Cat#A3500
Transcription Factor Buffer Set	B.D. Bioscience	Cat#562574
**Experimental Models: Organisms/ Strains**		
Mouse: C57BL/6J	Mouse*:* BALB/c	
**Cell Lines and Bacteria**		
3T3-L1 Cell (ATCC-CL173™)	*C. Rodentium* (ATCC51459)	
Oligonucleotides		
Gene	Sequence	
Gapdh-F	CTCATGACCACAGTCCATGC	N/A
Gapdh-R	CACATTGGGGGTAGGAACAC	N/A
Bactin-F	CTAAGGCCAACCGTGAAAAG	N/A
Bactin-R	ACCAGAGGCATACAGGGACA	N/A
18s-F	AGTCCCTGCCCTTTGTACACA	N/A
18s-R	CGATCCGAGGGCCTCACTA	N/A

36b4-F	ATGGGTACAAGCGCGTCCTG	N/A
36b4-R	GCCTTGACCTTTTCAGTAAG	N/A
Alldh1a1-F	CTCCTCTCACGGCTCTTCA	N/A
Alldh1a1-R	AATGTTTACCACGCCAGGAG	N/A
Alldh1a2-F	TTGCAGATGCTGACTTGGAC	N/A
Alldh1a2-F	TCT GAGGACCCTGCTCAGTT	N/A
Alldh1a3-F	GATAAAGTTGGG CTGAGCAA	N/A
Alldh1a3-R	CCAAAATTCAGTGTCCGAAG	N/A
Tnfsf13-F	TCACAATGGGTCAGGTGGTATC	N/A
Tnfsf13-R	TGTAAATGAAAGACACCTGCACTGT	N/A
Tnfsf13b-F	TGCTATGGGTCATGTCATCCA	N/A
Tnfsf13b-R	GGCAGTGTTTTGGGCATATTC	N/A
Tgfb-1-F	GCAACATGTGGAACTCTACCAGA	N/A
Tgfb1-R	GACGTCAAAAGACAGCCACTCA	N/A
Il10-F	GCTCTTACTGACTGGCATGAG	N/A
Il10-R	CGCAGCTCTAGGAGCATGTG	N/A
Cyp26a1-F	AAGGCGCGGAACCTTATACA	N/A
Cyp26a1-R	TCTTGGCGCGAATGTTCTC	N/A
Cyp26b1-F	AATGAGATTCTGCCGGAGACA	N/A
Cyp26b1-R	GAGGCTACACCGTAGCACTCAA	N/A
Cyp26c1-F	GGGACCAGTTGTATGAGCAC	N/A
Cyp26c1-R	AGCCAACTCCTTCAGCTCTT	N/A
Iα 253-F	CCAGGCATGGTTGAGATAGAGATAG	N/A
Cμ 94-R	AATGGTGCT GGGCAGGAAGT	N/A
Iα 254-F	CAGGCATGGTTGAGATAGAGATAGA	N/A
Cμ 93-R	ATGGTGCTGGGCAGGAAGTC	N/A
Aicda-F	GGCTGAGGTTAGGGTTCCATCTCAG	N/A
Aicda-R	GAGGGAGTCAAGAAAGTCACGCTGGA	N/A
Im-*F*	CTCTGGCCCTGCTTATTGTTG	N/A
Ia-*F*	CCTGGCTGTTCCCCTATGAA	N/A
Cm-*R*	GAAGACATTTGGGAAGGACTGACT	N/A
Hprt-F	GTTAAGCAGTACAGCCCCAAA	N/A
Hprt-R	AGGGCATATCCAACAACAAACTT	N/A
All bacteria-F	ACTCCTACGGGAGGCAGCAG	N/A
All bacteria-R	ATTACCGCGGCTGCTGG	N/A
SFB-*F*	GACGCTGAGGCATGAGAGCAT	N/A
SFB-*R*	GACGGCACGGATTGTTATTCA	N/A
Akk-F	CAGCACGTGAAGGTGGGGAC	N/A
Akk-R	CCTTGCGGTTGGCTTCAGAT	N/A

C. Rodentium-F	ATGCCGCAGATGAGACAGTTG	N/A
C. Rodentium-R	CGTCAGCAGCCTTTTCAGCTA	N/A
Zo-1-F	GGGCCATCTCAACTCCTGTA	N/A
Zo-1-R	AGAAGGGCTGACGGGTAAAT	N/A
Pigr-F	GCTCCAAAGTGCTGTTCTCC	N/A
Pigr-R	TTGCTGTGTGTCTGGAGAGG	N/A
Software and Equipment		
Flow Jo Version 8.0	Flow Jo, LCC	
Prism 8.0	GraphPad Softwar	
Image J Software 1.49	NIH	
96 Microplate Reader	Bio-Rad Laboratories	
Varioskan Lux Multimode Microplate Reader	ThermoFisher Scientific	
Nanodrop 2000 Spectrophotometer	ThermoFisher Scientific	
Accu-Check Performa Glucose Meter	Roche (USA)	
Nikon Eclipse Ni-U Fluorescent Microscope	Nikon Instruments Inc.	
Real-Time PCR System	Applied Biosystems	
ViiA7 Real-time PCR	Applied Biosystems	
Rm2235 Manual Microtome	Leica Microsystems	
Excelsior As Tissue Processor	ThermoFisher Scientific	
Paraffin Embedding Station	Thermolyne™	
BD Facsaria III Cell Sorter	B.D. Bioscience	
Dionex Ultimate 3000	Thermo Scientific	
Orbitrap Fusion Lumos Mass Spectrophotometer	Thermo Scientific	
TimsTOF Pro 2 mass spectrometer	Bruker Daltonics, USA	
Other		
Mouse normal Diet	Research Diet	Cat#5053
40-µm Cell Strainer	Falco	Cat#352340
70-µm Cell Strainer	Falco	Cat#352350
100-µm Cell Strainer	Falco	Cat#352360
Fluorotrans PVDF Transfer Membrane	Pall Life Sciences	Cat#BSP0161
Medical X-Ray Film	Fuji Film	Cat#RX1318
Superfrost™ Ultra Plus Adhesion Slides	ThermoFisher Scientific	Cat#10149870

Super Pap Pen	ThermoFisher Scientific	Cat#008899
Petri Dish, 100x15mm	SPL Life Sciences	Cat#10100

Reference

1. Wang W, Li Y, and Guo X. A mouse model of Citrobacter rodentium oral infection and evaluation of innate and adaptive immune responses. *STAR protocols.* 2020;1(3):100218.
2. Bouladoux N, Harrison OJ, and Belkaid Y. The mouse model of infection with Citrobacter rodentium. *Current protocols in immunology.* 2017;119(1):19.5. 1-.5. 25.
3. Mu Q, Tavella VJ, Kirby JL, Cecere TE, Chung M, Lee J, et al. Antibiotics ameliorate lupus-like symptoms in mice. *Scientific reports.* 2017;7(1):13675.
4. Lécuyer E, Rakotobe S, Lengliné-Garnier H, Lebreton C, Picard M, Juste C, et al. Segmented filamentous bacterium uses secondary and tertiary lymphoid tissues to induce gut IgA and specific T helper 17 cell responses. *Immunity.* 2014;40(4):608-20.
5. Jeppesen DK, Hvam ML, Primdahl-Bengtson B, Boysen AT, Whitehead B, Dyrskjøt L, et al. Comparative analysis of discrete exosome fractions obtained by differential centrifugation. *Journal of extracellular vesicles.* 2014;3(1):25011.
6. Wang CJ, Pao LH, Hsiong CH, Wu CY, Whang-Peng JJ, and Hu OY. Novel inhibition of cis/trans retinoic acid interconversion in biological fluids--an accurate method for determination of trans and 13-cis retinoic acid in biological fluids. *J Chromatogr B Analyt Technol Biomed Life Sci.* 2003;796(2):283-91.
7. Morgenstern J, Fleming T, Kliemank E, Brune M, Nawroth P, and Fischer A. Quantification of All-Trans Retinoic Acid by Liquid Chromatography-Tandem Mass Spectrometry and Association with Lipid Profile in Patients with Type 2 Diabetes. *Metabolites.* 2021;11(1).
8. Erben U, Loddenkemper C, Doerfel K, Spieckermann S, Haller D, Heimesaat MM, et al. A guide to histomorphological evaluation of intestinal inflammation in mouse models. *International journal of clinical and experimental pathology.* 2014;7(8):4557.

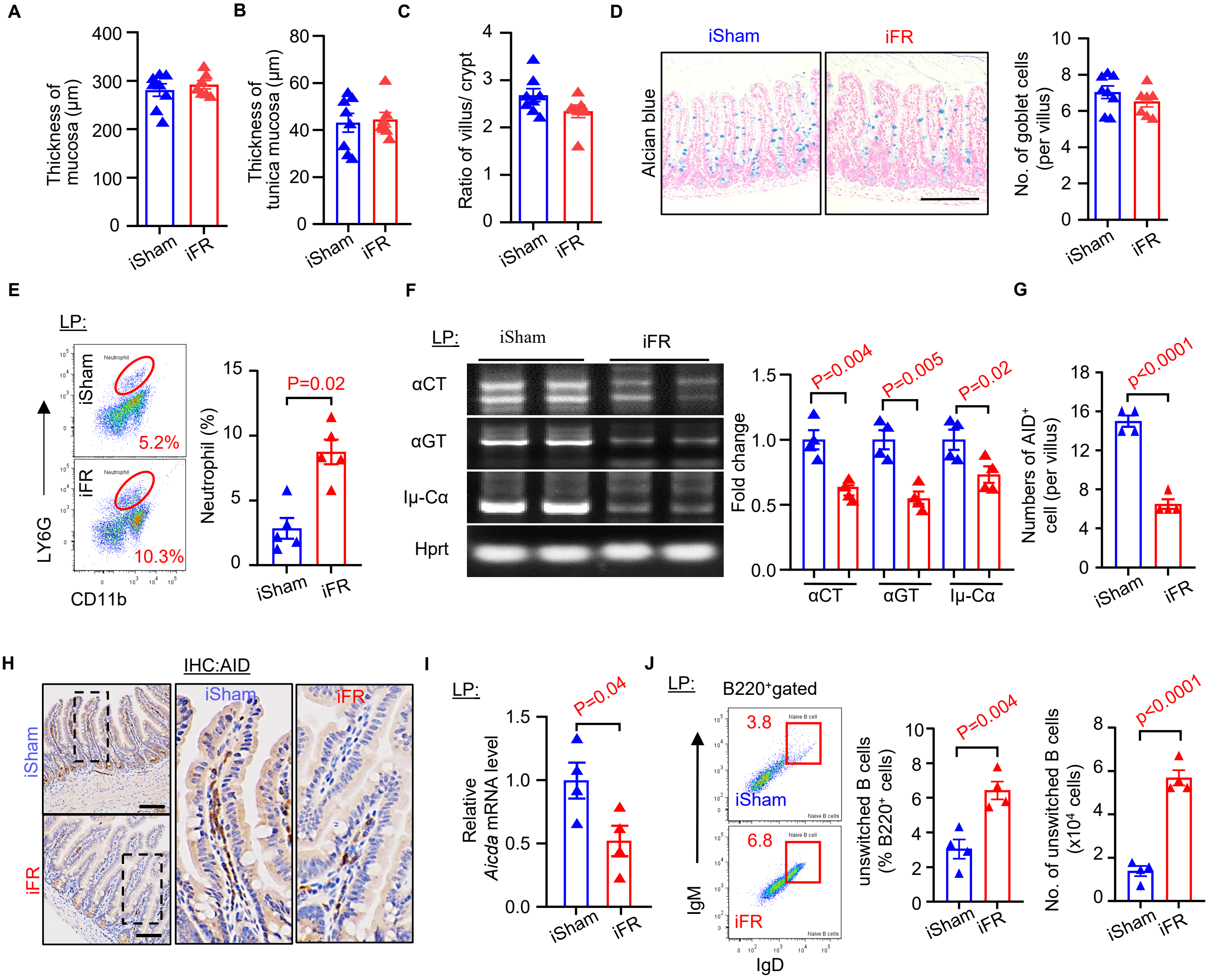

Supplementary Figure 1. Impairment of IgA class-switching in LP by surgical removal of iWAT. (**A-C**) Histological analysis of the ileum was performed based on H&E images in **Fig. 1G** (*n*=8). (**A**) Thickness of mucosa, (**B**) thickness of tunica mucosa, and (**C**) ratio of villus-to-crypt. (**D**) Alcian Blue staining. The right panel shows quantitative analysis of the number of goblet cells (*n*=8). Scale bar: 100 μm (**E**) Flow cytometry analysis of the percentage of neutrophils (CD11b$^+$LY6G$^+$ cells) in the LP (*n*=5). (**F**) Semi-quantitative PCR analysis of αGT, αCT, and Iμ-Cα transcripts. The quantitative analysis is shown in the right panel, the data normalized with *Hprt* and expressed as fold change relative to the expression in the iSham controls (*n*=4). (**G**) The number of AID-positive cells within the villus, expressed as fold change relative to the numbers in iSham controls (**G**), representative images were shown in (**H**). Scale bar: 100 μm. (*n*=4). (**I**) QPCR analysis of *Aicda* mRNA level, the targeted genes are normalized with *Hprt* (*n*=4). (**J**) The frequency and number of unswitched B cells in the LP. The frequency of cells was gated from CD45$^+$cells (*n*=4). Data are displayed as mean ± SEM. Statistical significance was determined using a two-tailed Student's t-test.

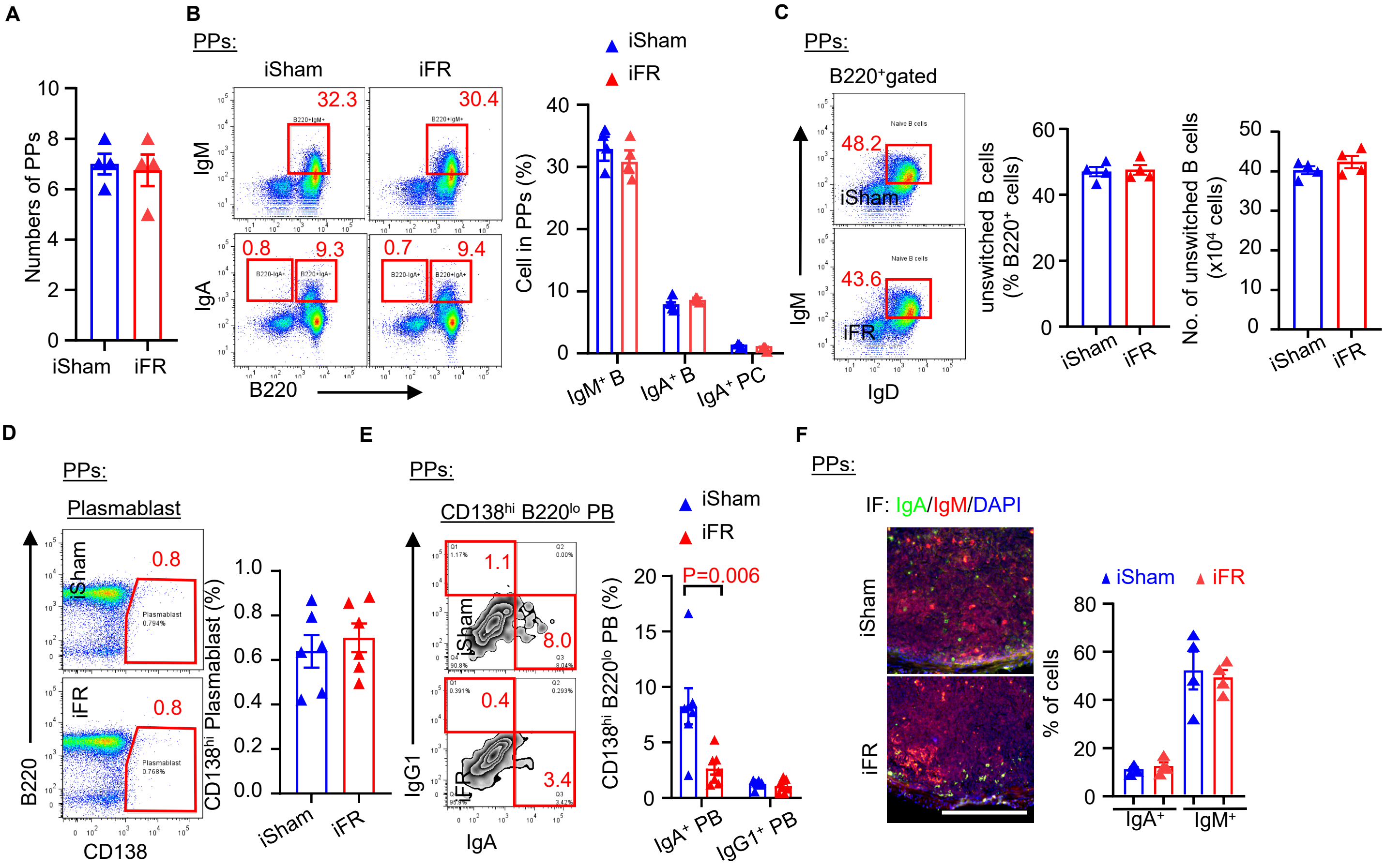

Supplementary Figure 2. Surgical iWAT removal has no obvious effect on the IgA^{+} cell population in the PPs of the small intestines. (**A**) Number of PPs on the small intestine. (**B-E**) Flow cytometry analysis for IgM^{+} B and IgA^{+} B, IgA^{+} PC, unswitched B cells, and CD138^{hi}B220^{lo} plasmablasts (PB) and PB gated IgA^{+} and IgG1^{+} cells. (**F**) Immunofluorescent staining of IgA, IgM, and DAPI in the PPs. The bar chart shows the percentage of IgA^{+} and IgM^{+} cells within PPs. *n*=5-6. Scale bar: 100 µm. Data are displayed as mean ± SEM. Statistical significance was determined using a two-tailed Student's t-test.

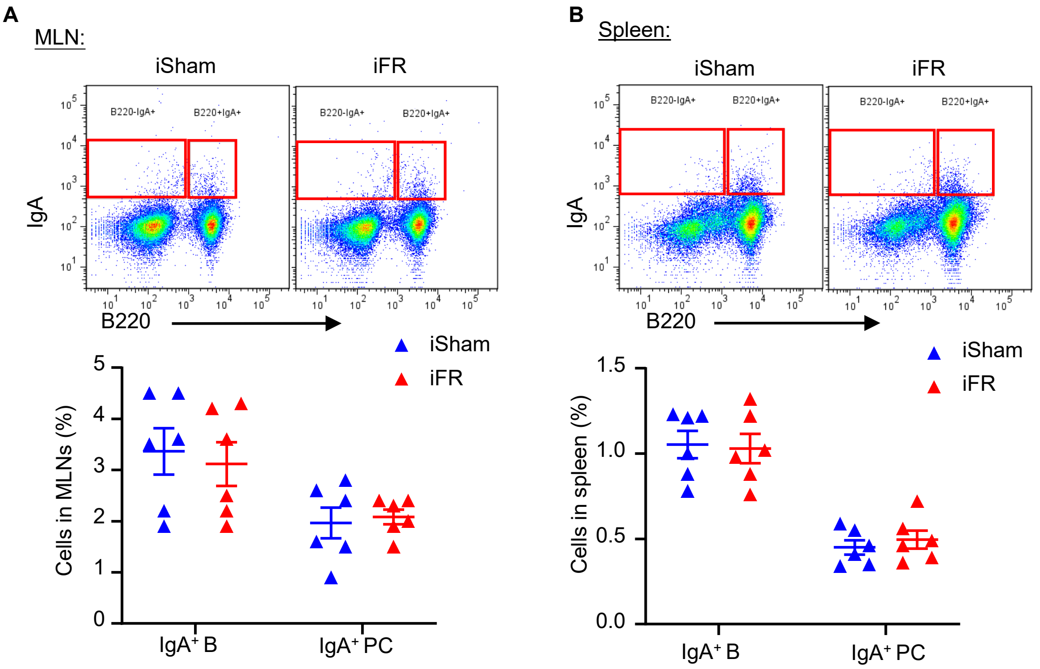

Supplementary Figure 3. iWAT removal has no obvious impact on IgA$^+$ cell population in the spleen and mesenteric lymph node. The frequencies of IgA$^+$ PC and IgA$^+$ B cells in MLNs (**A**) and spleen (**B**). All frequencies of cells were gated from CD45$^+$ cells. *n*=6. Data are displayed as mean ± SEM. Statistical significance determined using a two-tailed Student's t-test.

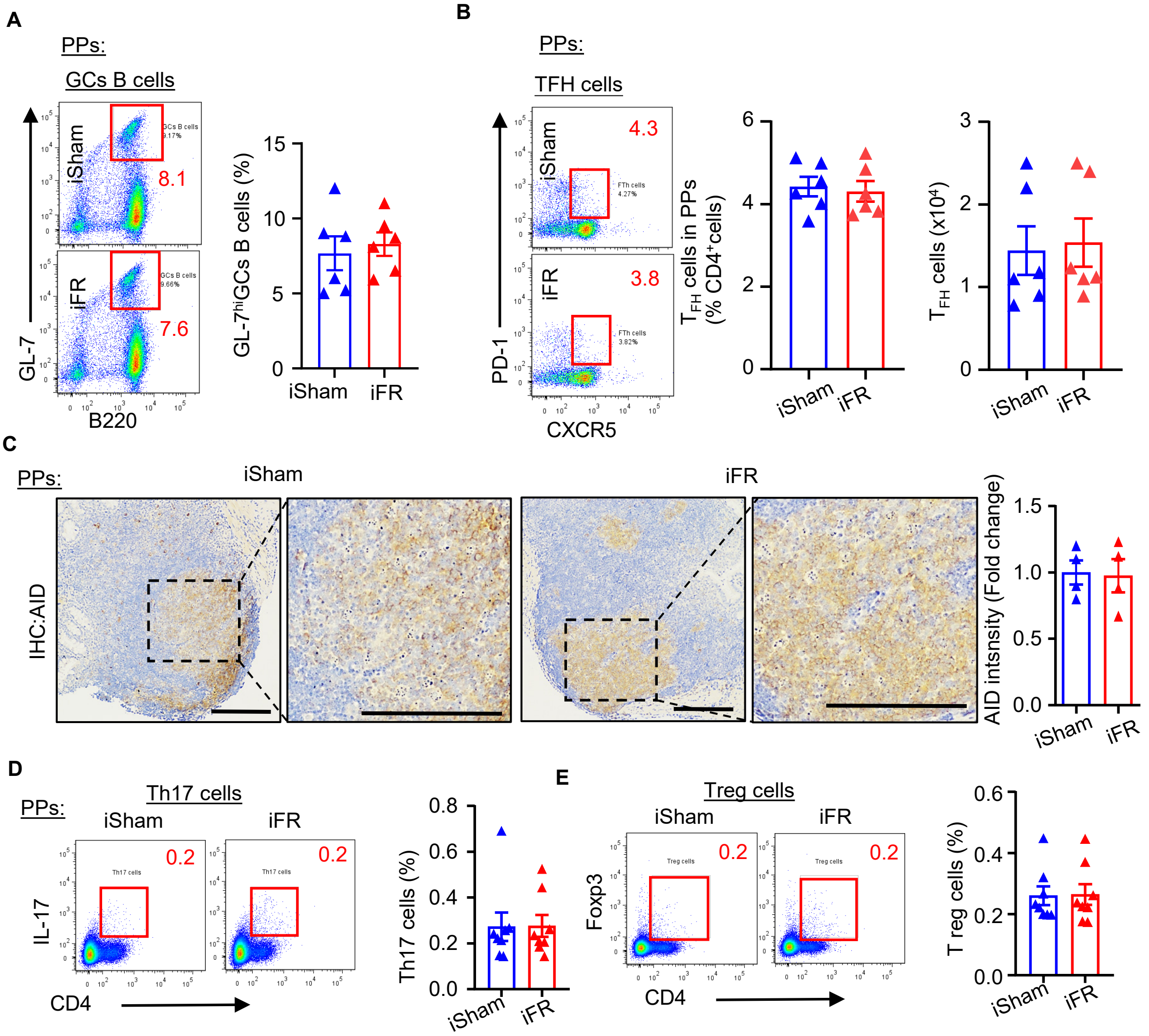

Supplementary Figure 4. iWAT removal does not affect IgA class switching and T cell populations in the PPs. (**A**) Frequencies of $B220^{+}GL\text{-}7^{+}$ GCs B cells and (**B**) $CD4^{+}$ cells gated $CXCR5^{+}PD\text{-}1^{+}$ T_{FH} cells (n=6). (**C**) Immunohistochemical staining of AID in the PPs. Scale bar: 100 μm (n=4). (**D**) Frequencies of $CD4^{+}$ $IL\text{-}17A^{+}$ Th17 cells and (**E**) $CD4^{+}$ $Foxp3^{+}$ T regulatory (Treg) cells (n=8). All frequencies of cells were gated from $CD45^{+}$ cells. Representative images are shown. Data are displayed as mean ± SEM. Statistical analysis was performed using a two-tailed Mann-Whitney U test on panel **A** and **D**, and others were analyzed using a two-tailed Student’s t-test.

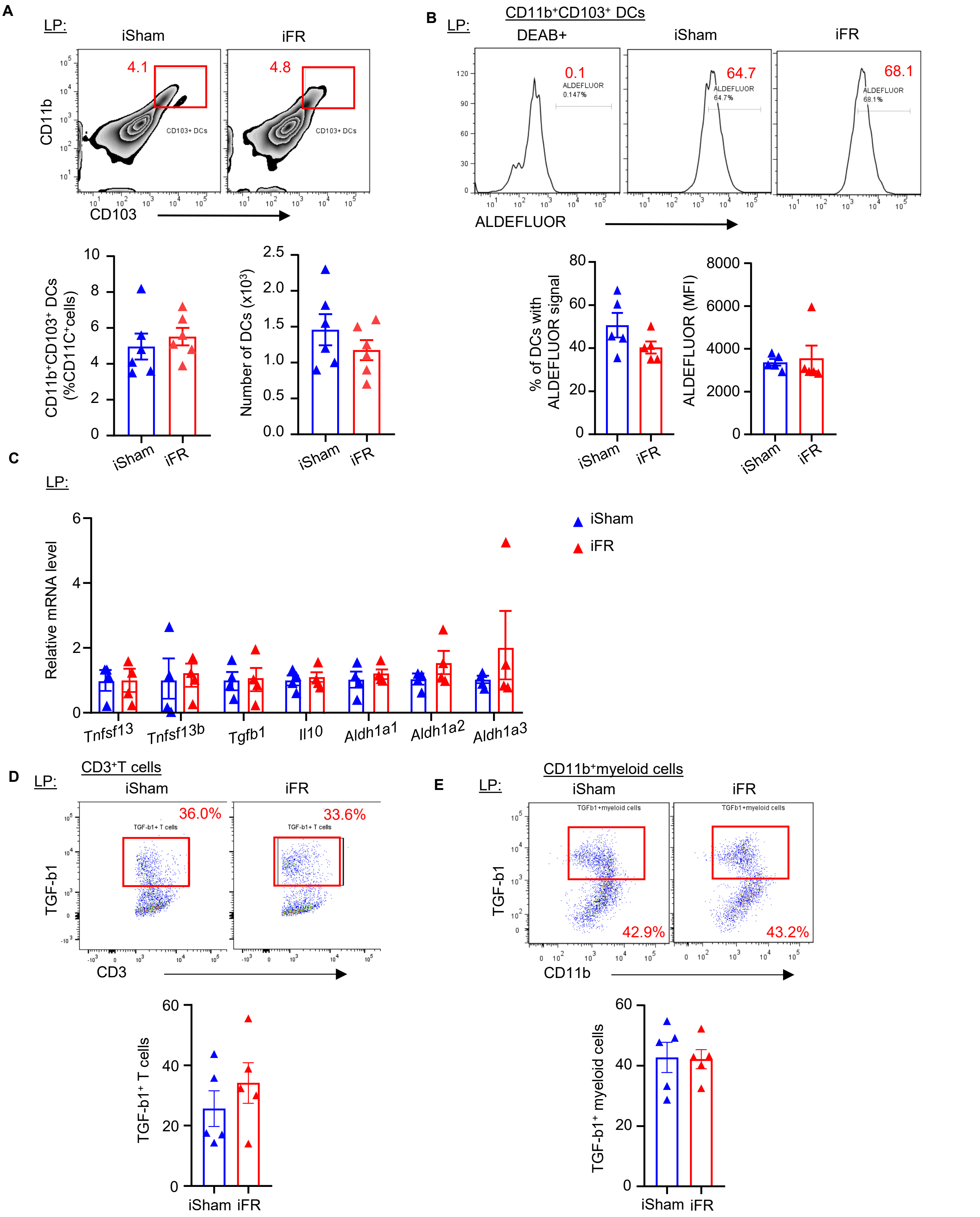

Supplementary Figure 5. Removal of iWAT does not alter the populations of IgA production-related T cells and DCs in the LP. (**A**) Frequencies and numbers of CD11c$^+$ gated CD11b$^+$CD103$^+$ dendritic cells (DCs), the frequencies of cells were gated from CD45$^+$ cells (*n*=6). (**B**) Aldehyde dehydrogenase activity in DCs using ALDEFLUOR™ assays (*n*=5). (**C**) mRNA levels related to T-independent pathway IgA production, the data are normalized with *gapdh* and expressed as fold change relative to the expression in the iSham controls. (**D-E**) Frequencies of CD45$^+$CD3$^+$ gated TGF-b1$^+$ T cells and CD45$^+$CD11c$^+$ gated TGF-b1$^+$ myeloid cells (*n*=5). Data are displayed as mean ± SEM. Statistical significance was determined using a two-tailed Mann-Whitney U test on **panel B, D,** and **C**-*Aldh1a3* expression, and others performed a two-tailed Student's t-test.

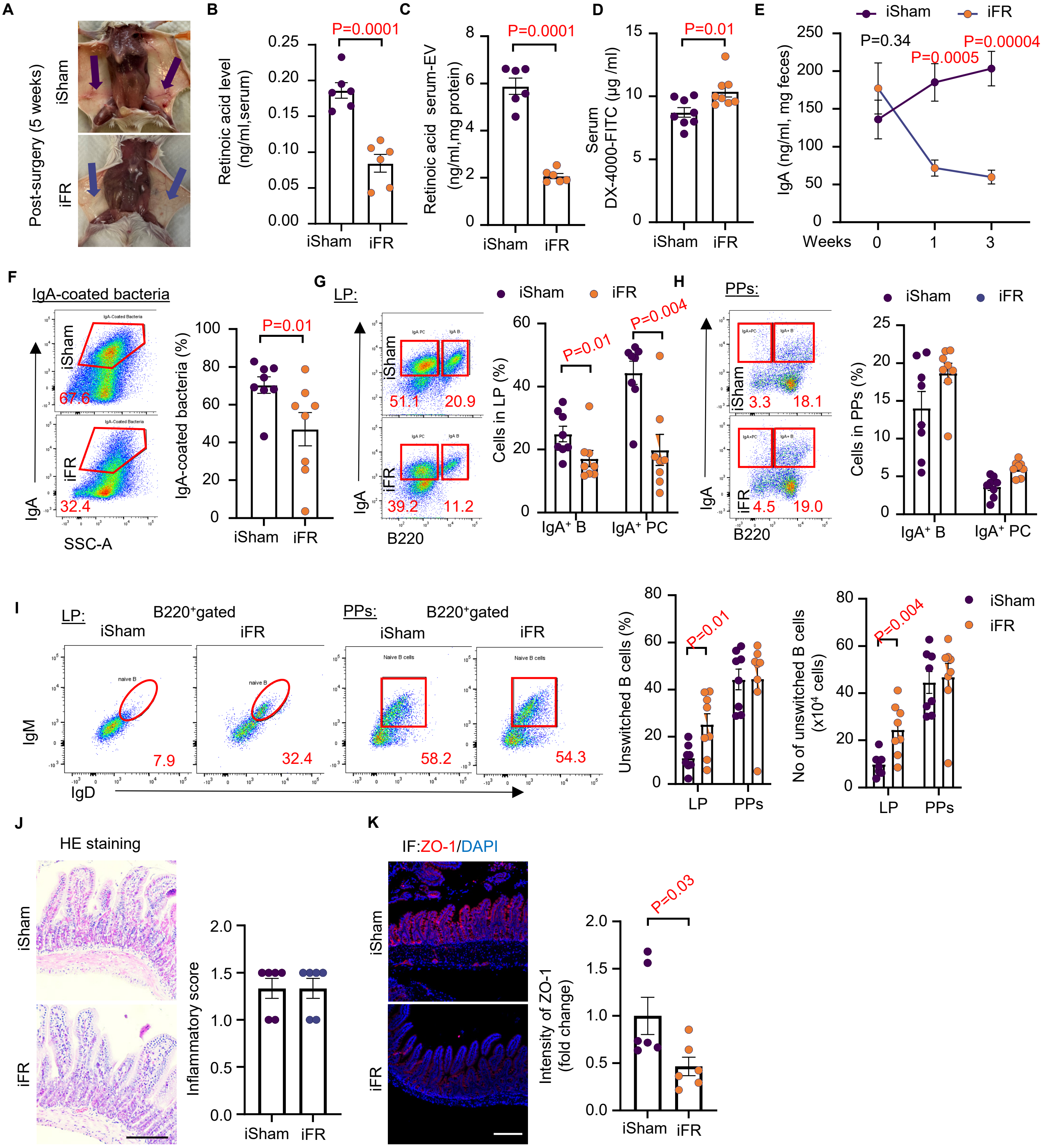

Supplementary Figure 6. iWAT removal reduces circulating RA levels and intestinal IgA production in BALB/c mice. (**A**) Representative images of the subcutaneous fat region in sham mice (purple arrows) and iFR mice (blue arrows). (**B-C**) RA levels in serum and serum-EV. (**D**) *In vivo* gut permeability test at week 4. (**E**) IgA level in the feces from week 0 (before the surgery) to week 3. (**F**) Percentage of IgA-coated bacteria. (**G-I**) Percentages of IgA$^+$B, IgA$^+$PC, and unswitched B cells in the LP and PPs. All frequencies of cells were gated from CD45$^+$cells. (**J**) H&E staining of the ileum. The right panel shows the inflammatory score. Scale bar: 100 μm. (**K**) Immunofluorescent staining of ZO-1 in the ileum. Scale bar: 100 μm. Representative images are shown. Data are displayed as mean ± SEM. *n*=6 for **panel B, C, J, and K**, *n*=8 for the remaining panels. Statistical analysis was performed using a two-tailed Mann-Whitney U test on **panel F, G, H**, **I, J,** and **K** and a two-tailed Student's t-test for the remaining panels.

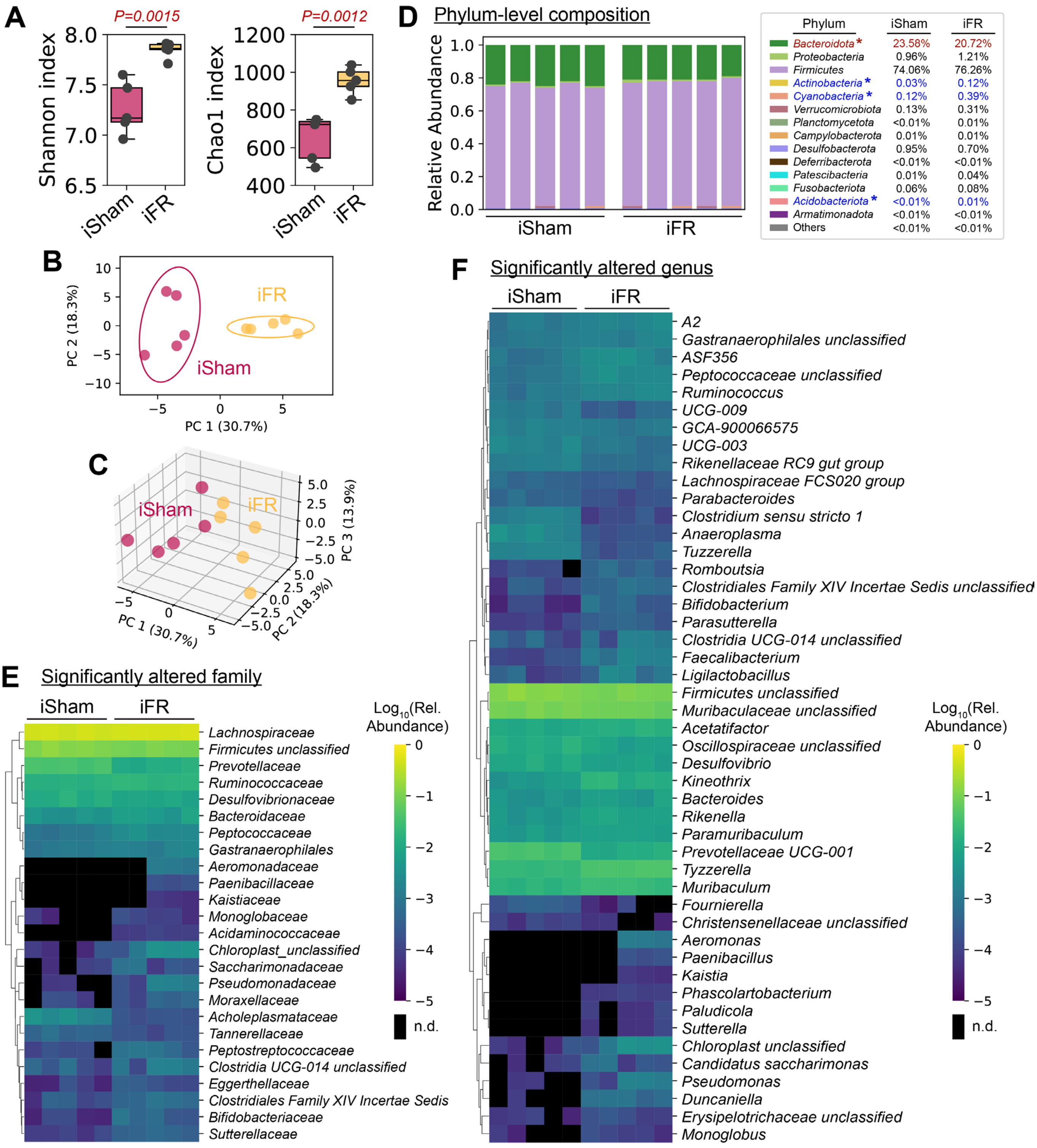

Supplementary Figure 7. iWAT removal alters microbiota composition in BALB/c mice. Microbiota in the cecum at week 5 from iSham and iFR BALB/c mice was subjected to 16S rRNA sequencing (*n*=6). (**A**) Alpha diversity analysis of the microbiota, presented as the Shannon index and Chao1 index. (**B-C**) PCA analysis. (**D**) Microbial at the phylum level. (**E**) Significantly changed (P<0.05) bacterial communities at the family level. (**F**) Significantly changed (P<0.05) bacterial communities at the genus level, actual p value is presented in **Supplementary Table 6** . n.d. indicates not detected. Statistical significance was determined using a two-tailed Student's t-test.

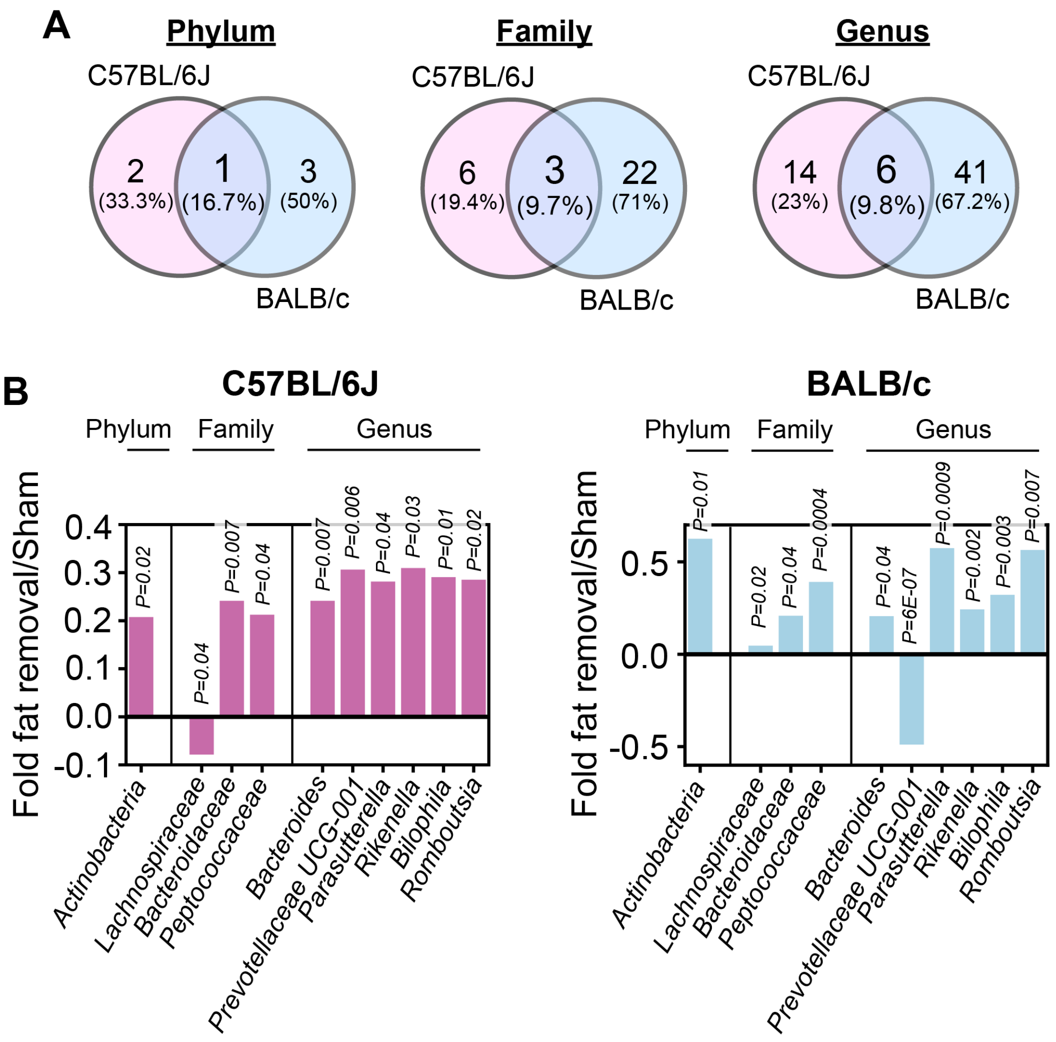

Supplementary Figure 8. Comparison of the effect of iWAT removal on gut microbiota composition between C57BL/6J mice and BALB/c mice. (**A-B**) Overlapped OTUs in altered genera, families, and phyla between the two mouse strains. Red color represents C57BL/6J mouse strain (*n*=6), while blue color represents BALB/c mouse strain (*n*=6). Statistical significance was determined using a two-tailed Student's t-test.

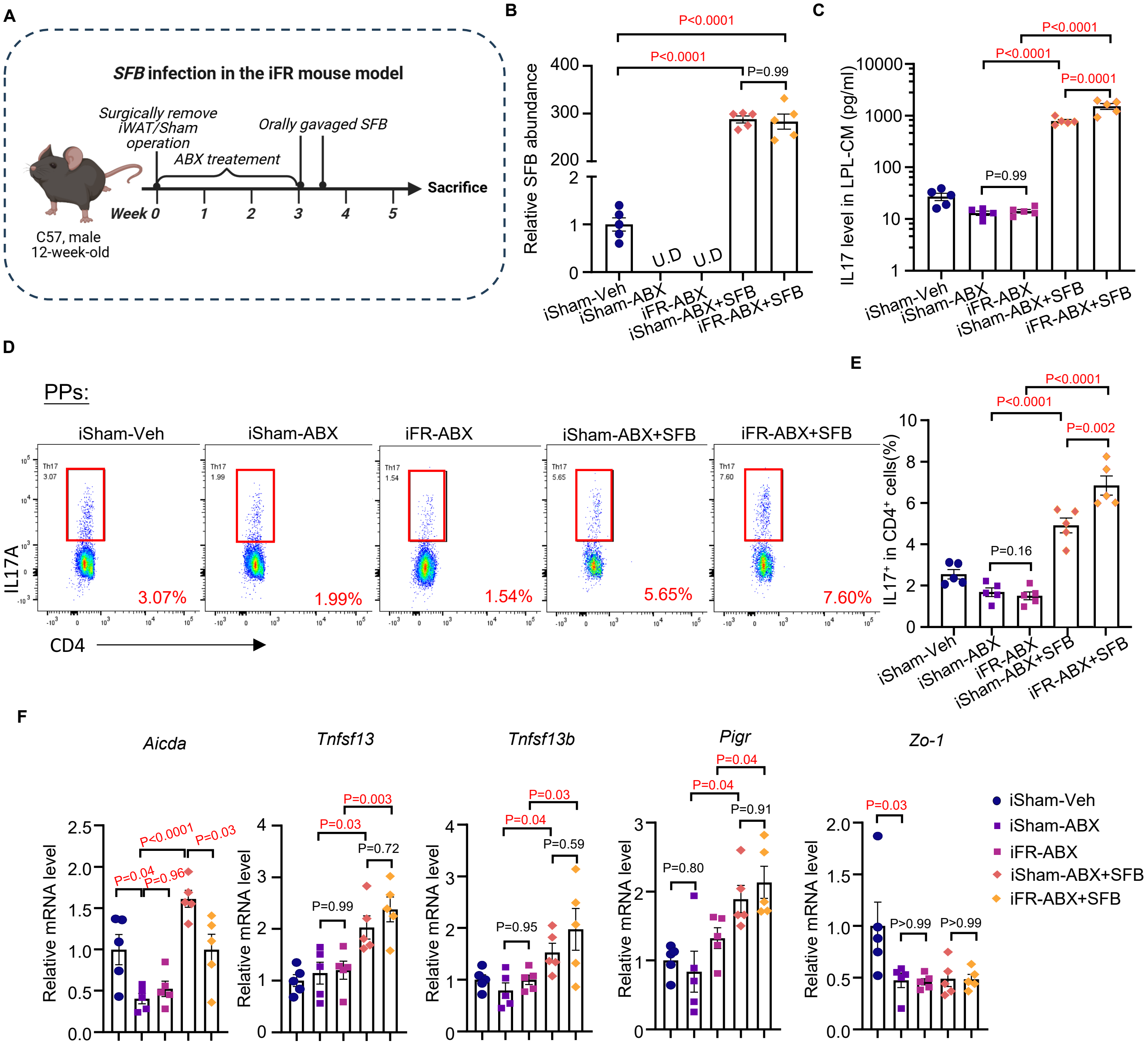

Supplementary Figure 9. iWAT removal enhances SFB-specific Th17 cells response in the pseudo-germ-free mice. (**A**) Schematic depicting the experimental paradigm. Post SFB colonization for 2 weeks, mice were sacrificed for the following assessments. (**B**) QPCR analysis of SFB in the mice feces. (**C**) Quantification of IL-17A secretion in 72 hours-culture supernatants of LP lymphocytes isolated from the small intestine of mice and stimulated by SFB lysate. (**D**) Flow cytometry analysis of IL17^{+} cells in the PPs. The cells gated from CD4^{+}CD45^{+}B220^{-} cells. (**E**) mRNA level of *Aicda*, *Tnfsf13*, *Tnfsf13b*, *Pigr*, and *Zo-1* in the ileum of mice. *n*=5. Data are displayed as mean ± SEM. Statistical significance was determined using One-way ANOVA with Tukey's multiple comparisons test.

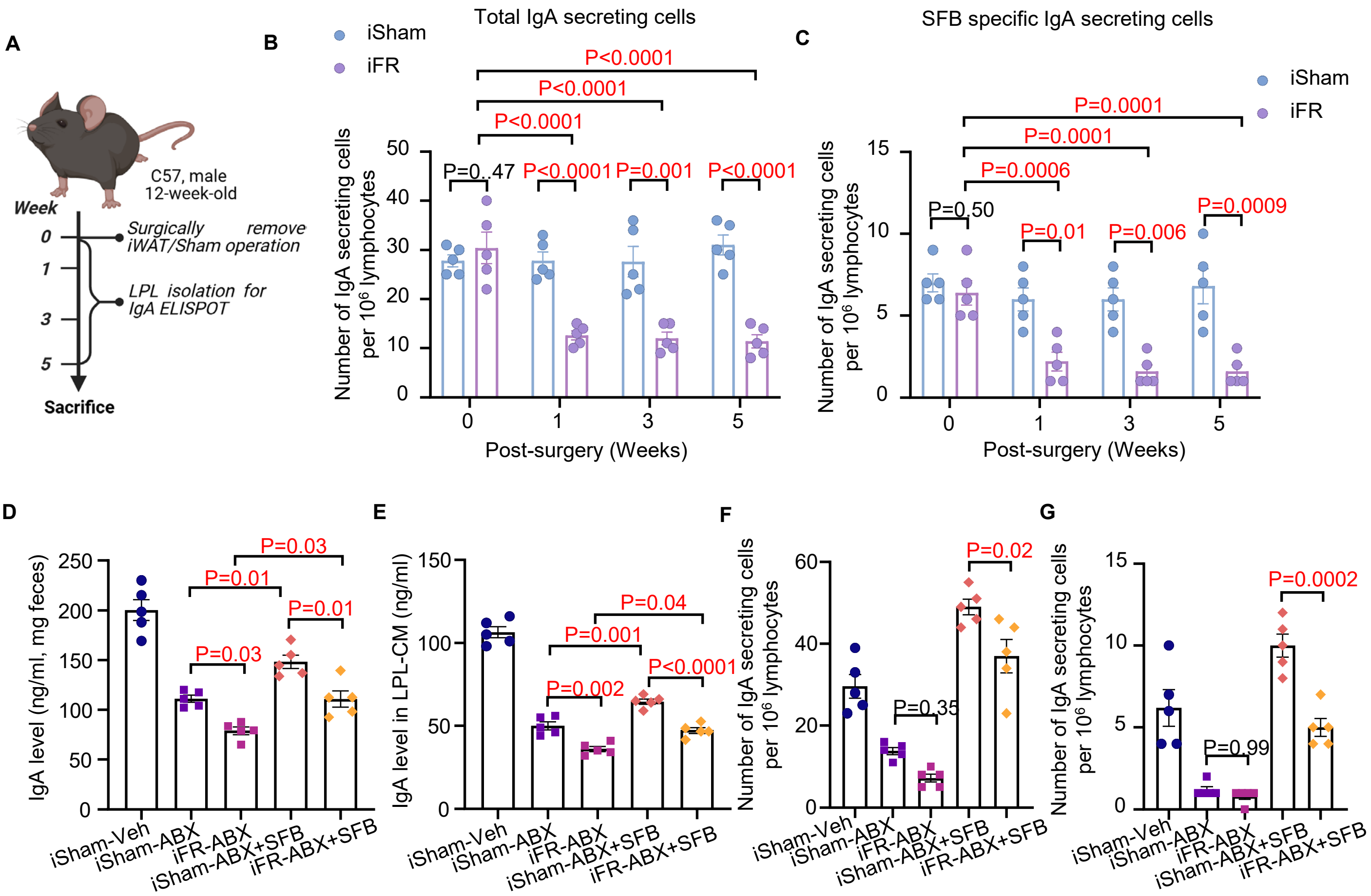

Supplementary Figure 10. iWAT removal reduced SFB-specific intestinal IgA production, its production is not maintained in LP. (**A**) Schematic depicting the experimental paradigm. (**B**) Number of total IgA-secreting cells and (**C**) SFB-specific IgA-secreting cells assessed by ELISpot. (**D-G**) Pseudo-germ-free iFR mice post SFB colonization for 2 weeks, and mice were sacrificed for the following assessments. (**D**) Fecal IgA level. (**E**) IgA level in the conditioned medium of LPL. (**F**) Number of total IgA-secreting cells. (**G**) Reactivity of IgA-SC from LP against SFB lysate assessed by ELISpot. *n*=5. Data are displayed as mean ± SEM. Statistical significance was determined using One-way ANOVA with Tukey's multiple comparisons test for panels **B-C**, and Two-way ANOVA with Tukey's multiple comparisons test for panels **D-G**.

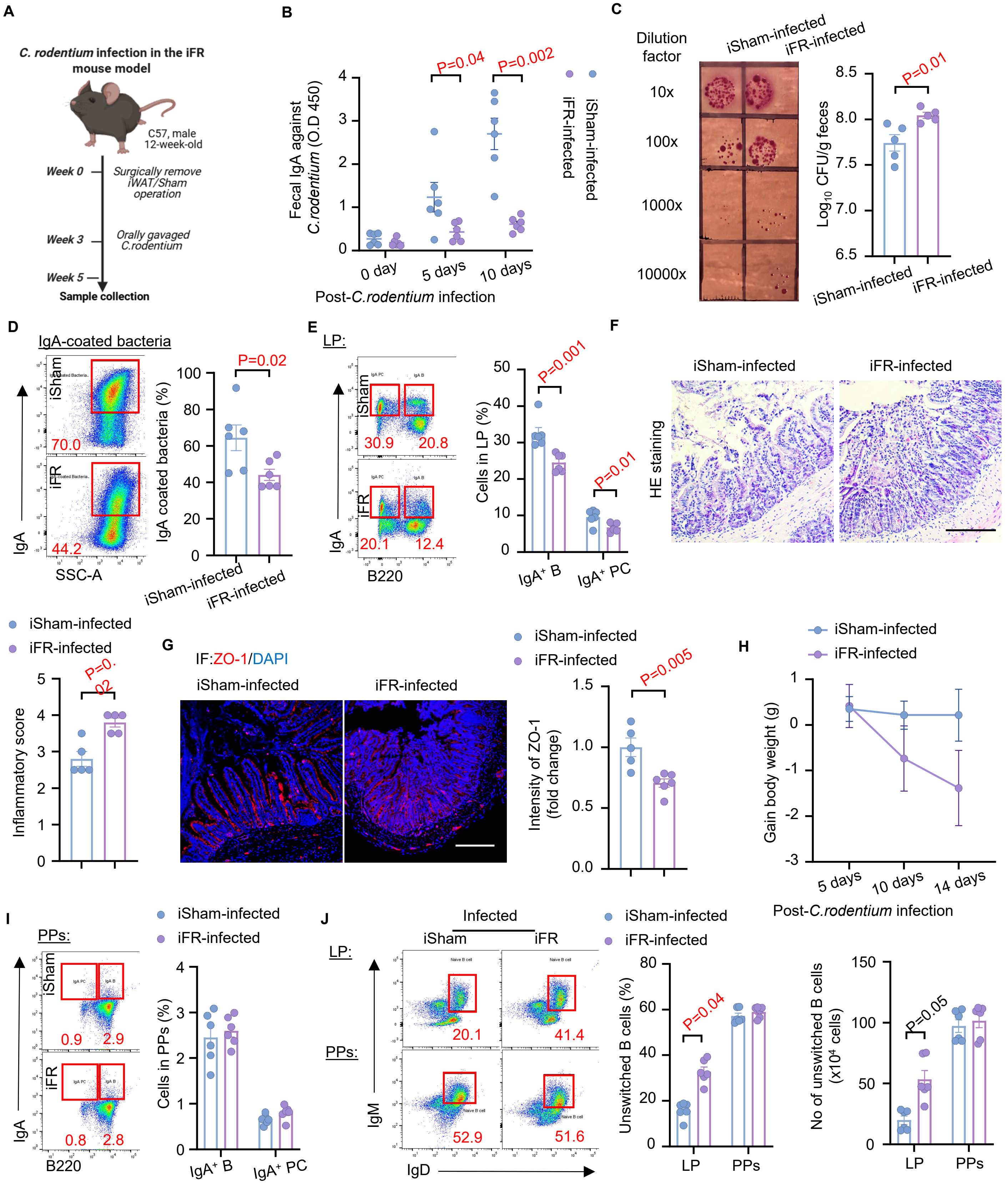

Supplementary Figure 11. iWAT protects intestinal function after *C. rodentium* infection in mice. (**A**) Schematic depicting the experimental paradigm. (**B**) Fecal IgA against *C. rodentium* level post-infection. (**C**) CFU of *C. rodentium* dot on the MacConkey Agar plate in a 10-fold dilution gradient in feces post-infection for 2 weeks. (**D**) Percentage of IgA-coated bacteria. (**E**) Percentages of IgA^+ B cells, IgA^+PC, and unswitched B cells in the LP. (**F**) H&E staining and (**G**) Immunofluorescent staining of ZO-1 in the ileum. Scale bar: 100 μm. (**H**) The gain body weight post -*C. rodentium* infection. (**I-J**)Percentages of IgA^+ B cells, IgA^+PC, and unswitched B cells in the LP and PPs. All frequencies of cells were gated from $CD45^+$ cells. Representative images are shown. Data are displayed as mean ± SEM (*n*=5-6). Statistical significance was determined using a two-tailed Mann-Whitney U test on panel **B**, and a two-tailed Student's t-test for the other panels.

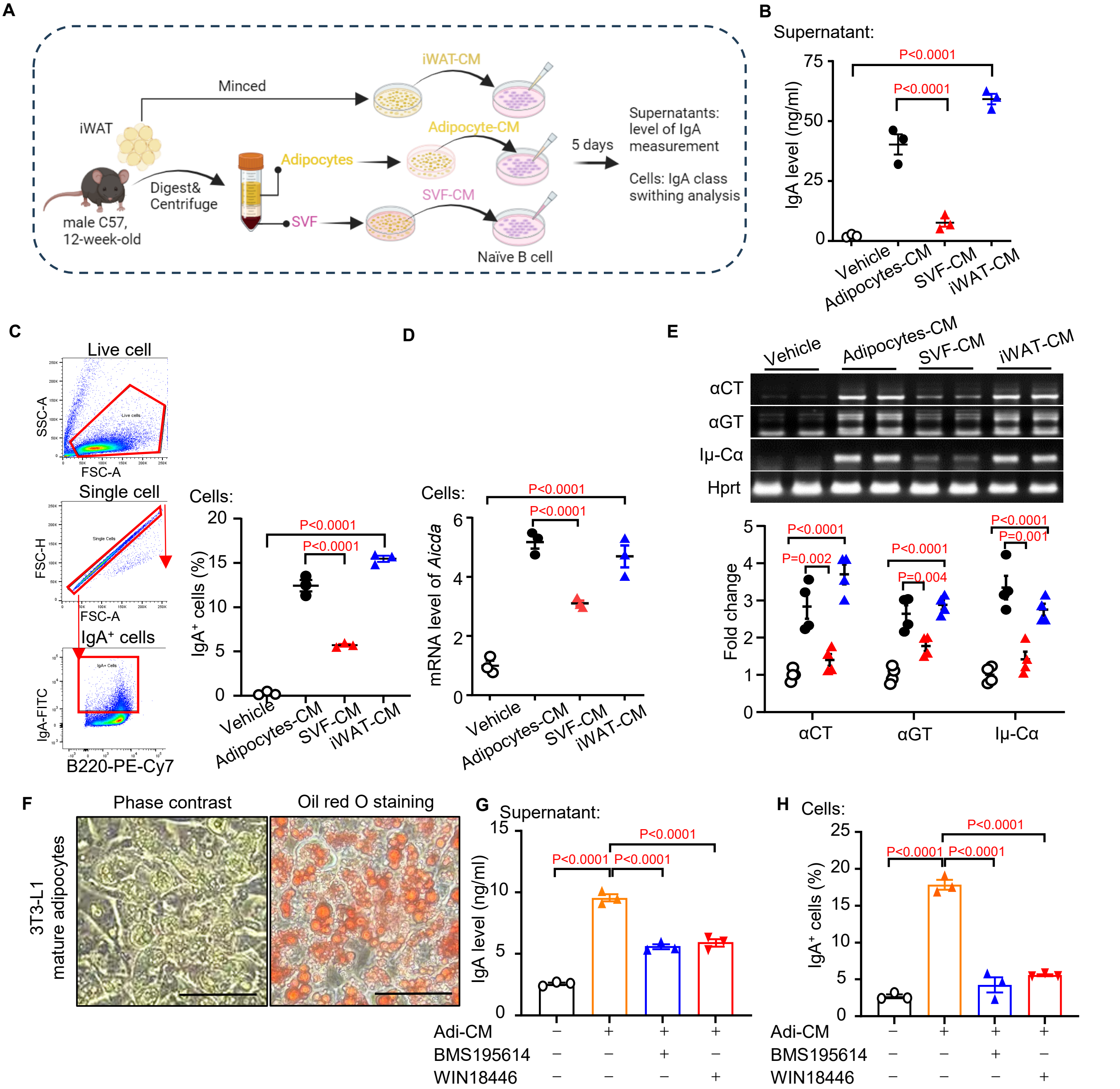

Supplementary Figure 12. Adipocyte-derived RA promotes IgA production and B-cell differentiation. (**A-E**) In vitro culture of unswitched B cells with CM collection from iWAT, adipocytes, and stromal vascular fractions (SVF) (*n*=3). (**A**) Schematic depicting the experimental paradigm. (**B**) IgA level in the supernatant at day 5. (**C**) Frequency of IgA$^+$ cells. (**D**) QPCR analysis of *Aicda* mRNA level. (**E**) Semi-QPCR analysis of αGT, αCT, and Iμ-Cα transcripts. Representative images are shown. (**F-H**) CM from 3T3-L1 differentiated mature adipocyte (Adi), Adi-CM pre-treated with WIN18446 or BMS195614 were used for in vitro culture of unswitched B cells for 5 days. RPMI1640 medium was used as a vehicle control (*n*=3). (**F**) Visualization of adipocyte using phase contrast imaging and Oil Red O staining. (**G**) IgA level in the supernatant at day 5. (**H**) Frequency of IgA$^+$ cells. Data are displayed as mean ± SEM. Statistical significance was determined using One-way ANOVA with Tukey's multiple comparisons test.

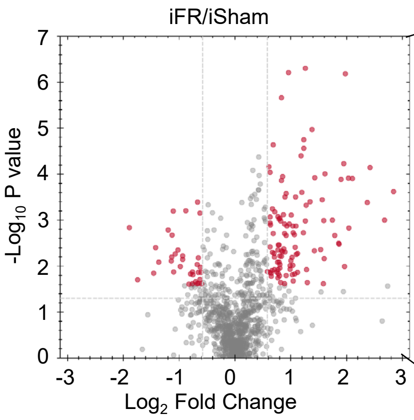

Downregulated proteins (adjusted P<0.05)

Down regulated protein

Protein	Log2 FC
POLE2	-1.67
GM5938	-1.26
SELE	-1.07
NID1	-0.99
PRDX4	-0.82
TRY4	-0.75
GLYCAM1	-0.72
PROCR	-0.66
CES2G	-0.65
IGF1	-0.6
ITM2B	-0.57
PCBP1	-0.56
HBB-BH0	-0.51
FCGBPL1	-0.47
ORM1	-0.42
ARF1	-0.41
CFD	-0.4
CTSL	-0.39
PGAM1	-0.34

Upregulated proteins (adjusted P<0.05)

Up regulated protein

Protein	Log2 FC
RELN	0.27
CLEC3B	0.29
PDGFRA	0.31
GOT1	0.32
MMRN1	0.33
SERPINA6	0.33
H6PD	0.34
ALCAM	0.36
FAM20C	0.36
COL1A1	0.37
PGD	0.38
APOH	0.39
COL1A2	0.42
TREML1	0.44
B4GALNT1	0.47
CD84	0.48
PTPRM	0.48
TFPI	0.48
IGFBP7	0.5
PTPRG	0.51
OSTF1	0.51
C1S2	0.53
PTPRZ1	0.55
LEPR	0.55
SPP2	0.57
CTLA2A	0.63
MIB1	0.64
CAPZB	0.64
DYRK4	0.66
PARK7	0.66
S100A9	0.73
B4GAT1	0.73
REG1	0.74
MB	0.75
MATR3	0.85
OBP1B	0.88
WWC1	0.91
QRSL1	0.94
SLPI	1.21

Supplementary Figure 13. iWAT removal changes of the circulating proteome profile in mice. Proteomics analysis was performed to detect the differential expression of proteins in serum samples (iSham, *n*=4; iFR, *n*=5). Volcano plots display $\log_2$ fold changes of protein expression on the x-axis and the $-\log_{10}$ P value on the y-axis. Differentially expressed proteins are highlighted in red and presented on the right.

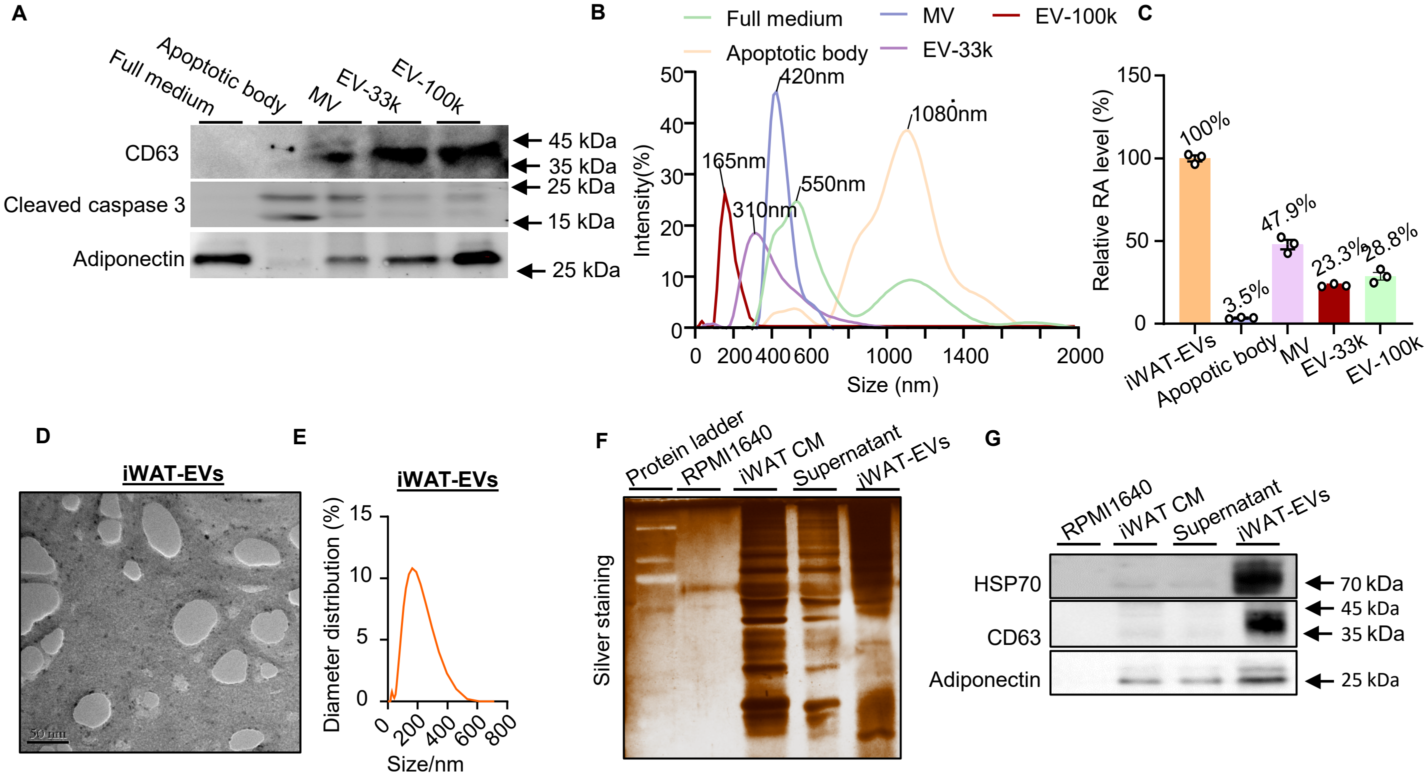

Supplementary Figure 14. Characteristics of iWAT-EVs. (**A**) Immunoblotting analysis of CD63, Cleaved caspase 3, and adiponectin in the iWAT-CM, iWAT-CM isolated apoptotic body, microvesicle (MV), and EVs through g-forces of 33000 (EV-33k) and EV-100k. (**B**) Size distribution of iWAT-conditioned full medium, apoptotic body, MV, EV-33k, and EV-100k. (**C**) Relative RA level in the indicated fractions. (**D**) Representative electron microscopic images of iWAT-EVs. Scale bar: 50 nm. (**E**) Size distribution of iWAT-EVs. (**F**) Representative image of silver staining showing the protein loading of RPMI1640 medium, iWAT-CM, supernatant, and iWAT-EVs. (**G**) Immunoblotting analysis of HSP70, CD63, and adiponectin in the RPMI1640 medium, iWAT-CM, supernatant, and iWAT-EVs. Results are shown from one experiment, representing at least two independent experiments.

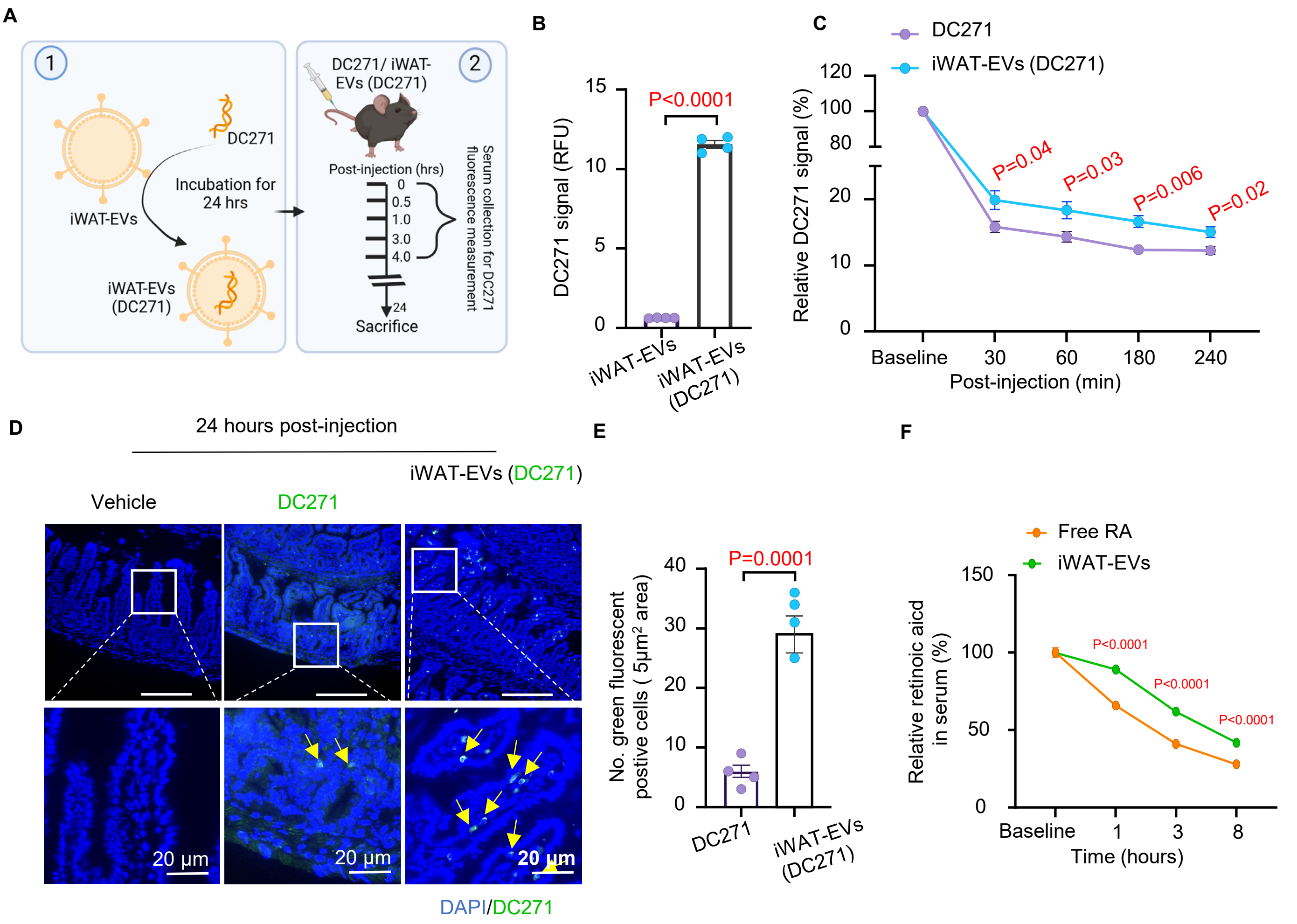

Supplementary Figure 15. DC271 encapsulated within iWAT-EVs exhibits enhanced stability and B cell targeting compared to free DC271. (A) An equivalent amount of DC271 and DC271 encapsulated in iWAT-EVs (referred to as 'iWAT-EVs (DC271)') were intraperitoneally injected into 8-week-old C57BL/6J male mice (*n*=3-4). (**B**) The signal of DC271 in both iWAT-EVs and iWAT-EVs (DC271), assessed at an excitation wavelength of 350 nm and emission wavelength of 540 nm, expressed as Relative Fluorescence Unit (RFU). (**C**) Relative serum DC271 concentrations at specified time points post-injection, normalized to baseline DC271 level. (**D**) Intestinal images at 24 hours post-injection。Scale bar, 100 µm. (**E**) Number of green fluorescent cell in the intestine 24 hours post-injection. (**F**) Free RA and iWAT-EVs were incubated in RA-free mouse serum for 8 hours. The relative content of RA is presented as the percentage of RA over the content of RA at baseline (0 hour) (*n*=4). Data presented as mean ± SEM. Statistical significance was determined using a two-tailed Student's t-test.

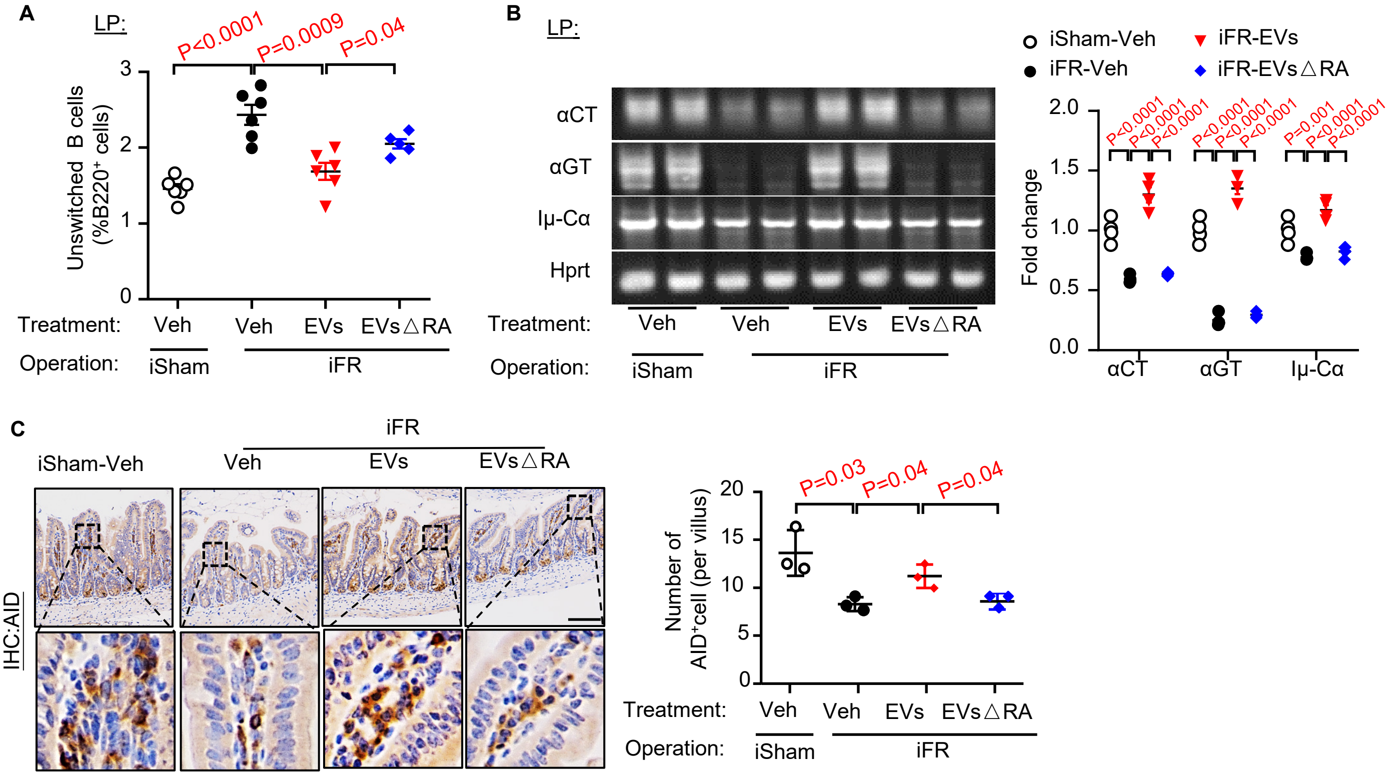

Supplementary Figure 16. iWAT-EVs treatment reverses the defective IgA class-switching in iFR mice. Ileac LP from the small intestine was isolated and used. (**A**) Percentage of unswitched B cells. The frequencies of cells were gated from $CD45^{+}$cells (*n*=5-6). (**B**) Semi-QPCR analysis of the class-switching markers. The data are normalized with Hprt and are presented as fold change relative to the iSham-Veh. Representative images are shown. (**C**) Immunohistochemical staining of AID in the small intestine (*n*=3). Scale bar: 100 μm. Representative images are shown. Data are displayed as mean ± SEM. Statistical significance was determined using One-way ANOVA with Tukey's multiple comparisons test.

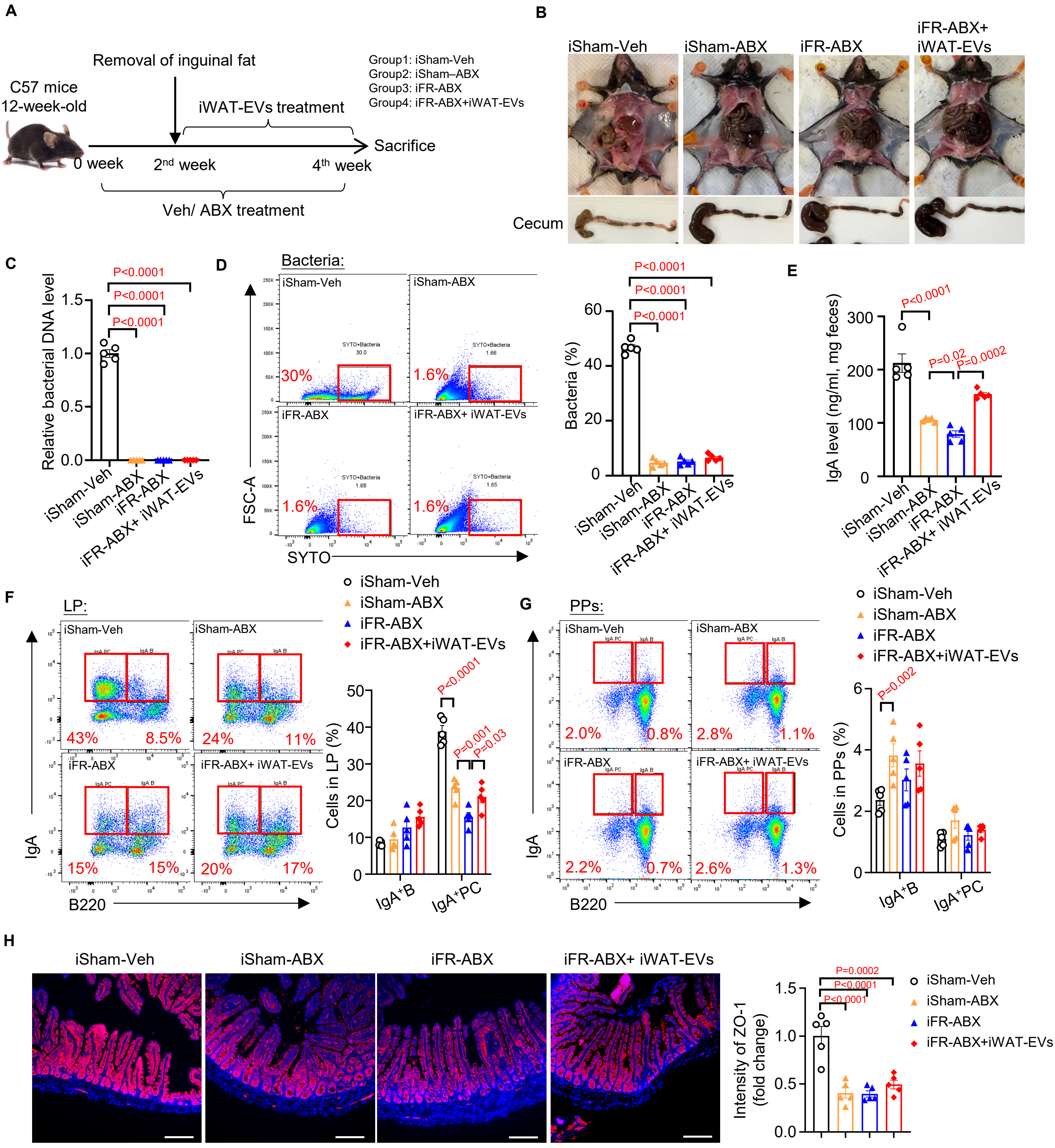

Supplementary Figure 17. The effect of iWAT-EVs on IgA production is gut microbiota independent. (**A**) Schematic depicting the experimental paradigm. (**B**) Representative image of enlarged size of cecum after ABX treatment in the mice. (**C**) Bacteria abundance in the cecum. (**D**) Percentage of bacteria from isolated ileum content. (**E**) Fecal IgA level at the end time point. (**F-G**) The frequencies of IgA^{+}B cells and IgA^{+} PCs from LP and PPs. (**H**) Immunofluorescent staining of ZO-1 in the ileum. Scale bar: 100 μm. *n*=5. Data are displayed as mean ± SEM. Statistical significance was determined using One-way ANOVA with Tukey's multiple comparisons test.

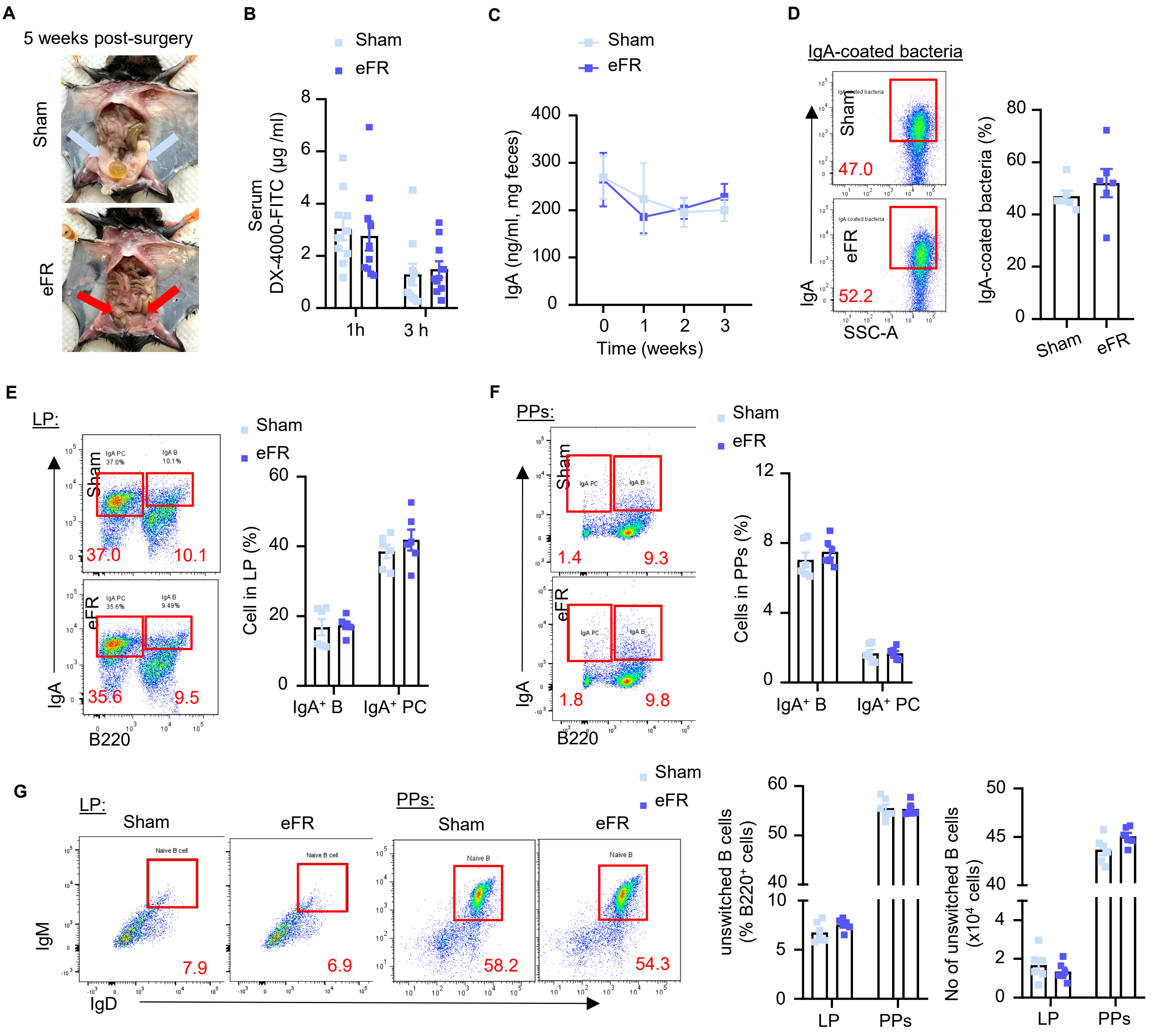

Supplementary Figure 18. Surgical removal of visceral epididymal white adipose tissue has no impact on the intestinal function. 12-week-old male C57BL/6J mice were subjected to surgical epididymal fat removal (eFR) or a sham operation (Sham). (**A**) Representative images of the epididymal fat region in Sham mice (blue arrows) and eFR mice (red arrows). (**B**) *In vivo* gut permeability test. (**C**) Fecal IgA level at 0-, 1-, 2-, and 3-week post-surgery. (**D**) Percentage of IgA-coated bacteria in the ileum. (**E-G**) Percentages of IgA^+ B cells, IgA^+PC, and unswitched B cells in the LP and PPs. All frequencies of cells were gated from $CD45^+$ cells. Representative images are shown. Data are displayed as mean ± SEM. *n*=10 for **panel B** and *n*=6 for the remaining panels. Statistical analysis was performed using a two-tailed Mann-Whitney U test on **panel B** whereas others were performed using a two-tailed Student's t-test.

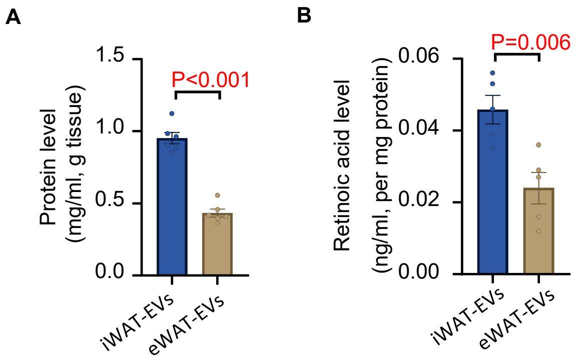

Supplementary Figure 19. Production of EVs and their RA capacity in iWAT and eWAT. (**A**) Protein level in EVs from an equal amount of iWAT and eWAT. (**B**) RA level in the iWAT-EVs and eWAT-EVs. *n*=5. Data are displayed as mean ± SEM. Statistical significance was determined using a two-tailed Student's t-test.

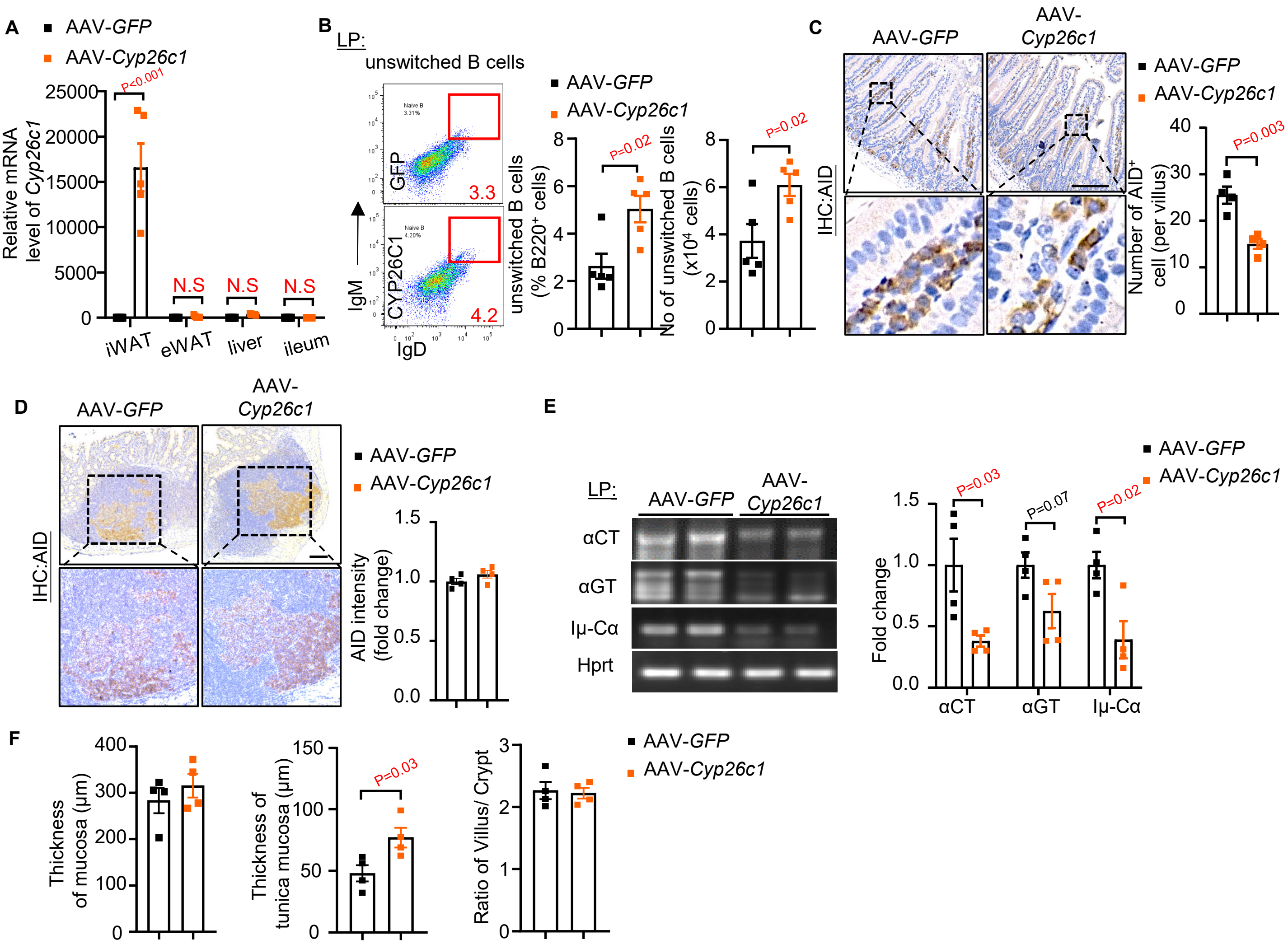

Supplementary Figure 20. AAV-*Cyp26c1* injection into iWAT impairs IgA class switching in the LP. (**A**) QPCR analysis of *Cyp26c1* expression in iWAT, eWAT, liver, and ileum. The data are normalized with *gapdh* (*n*=5). (**B**) Flow cytometric analysis of unswitched B cells. The cells were gated from CD45^{+} B220^{+}cells (*n*=5). (**C-D**) Immunohistochemical staining of AID in the small intestine (**C**) and PPs (**D**) with the quantitative analysis. Scale bar: 100 µm (*n*=4). (**E**) Representative images of semi-QPCR analysis of the class-switching markers in the LP and their quantitative analysis (*n*=4). (**F**) The quantitative analysis of mucosa thickness, tunica mucosa thickness, and villus-to-crypt ratio in the ileum. The representative images were shown in **Figure 7J** (*n*=4). Data are displayed as mean ± SEM. Statistical analysis was performed using a two-tailed Mann-Whitney U test on **panel A and E**, whereas the others were performed using a two-tailed Student's t-test.

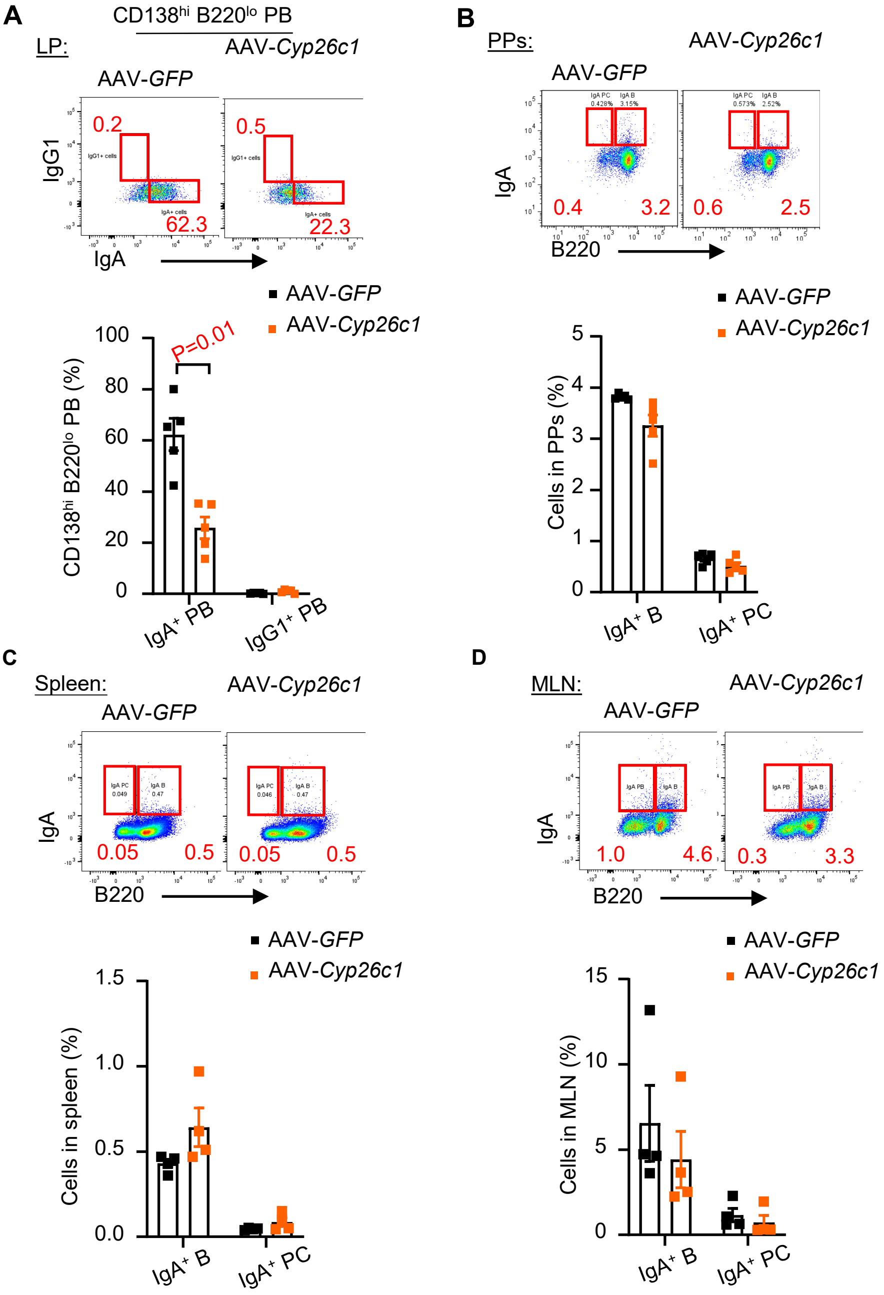

Supplementary Figure 21. Injection of AAV-*CYP26C1* into iWAT does not alter IgA populations in other immune organs. (**A**) Flow cytometry analysis of frequencies of IgA^+ and $IgG1^+$ cells gated by $CD138^{hi}B220^{lo}$ plasmablasts (PB) in the LP. (**B-D**) Flow cytometry analysis of frequencies of IgA^+ B cells and IgA^+PC in the PPs (**B**), spleen (**C**), and mesenteric lymph node (MLN) (**D**). All frequencies of cells were gated from $CD45^+$ cells. Representative images are shown. *n*=4. Data are displayed as mean ± SEM. Statistical analysis was performed using a two-tailed Mann-Whitney U test on **panel C and D**, whereas the others were performed using a two-tailed Student's t-test.

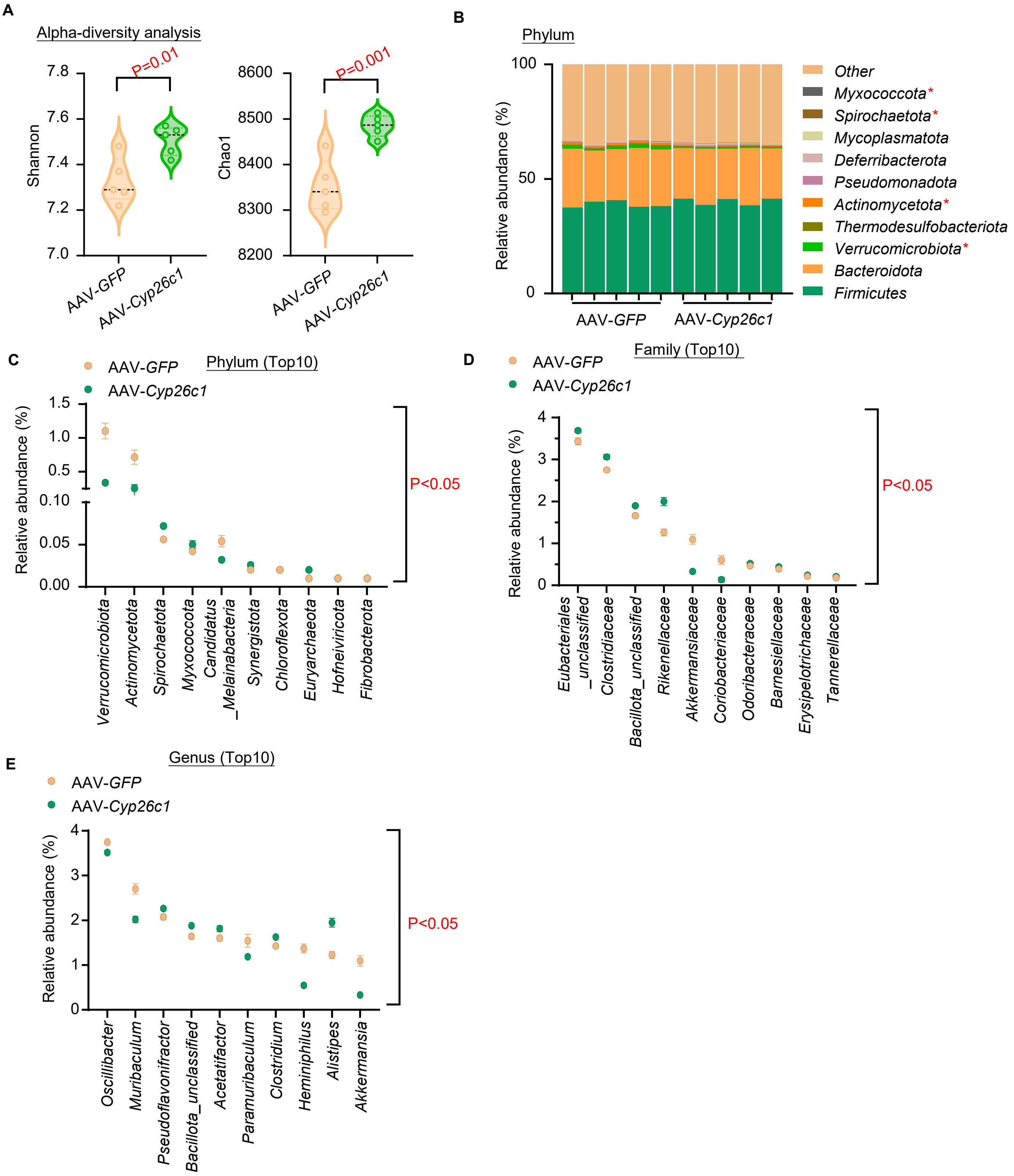

Supplementary Figure 22. Injection of AAV-*Cyp26c1* into iWAT alters microbiota composition.
Bacterial DNA was isolated from the cecum of mice at week 5 post AAV-*Cyp26c1*/*GFP* injection, followed by metagenomic sequencing (n=5). (**A**) Alpha diversity analysis of the microbiota, presented as the Shannon index and Chao1 index. (**B**) The top 10 abundant microbial at the phylum level. (**C-E**) The top 10 significantly changed bacterial communities at the phylum level (**C**), family level (**D**), and genus level (**E**). Actual p value is presented in **Supplementary Table 6**. Data are displayed as mean ± SEM. Statistical analysis was performed using two-tailed Student's t-test.

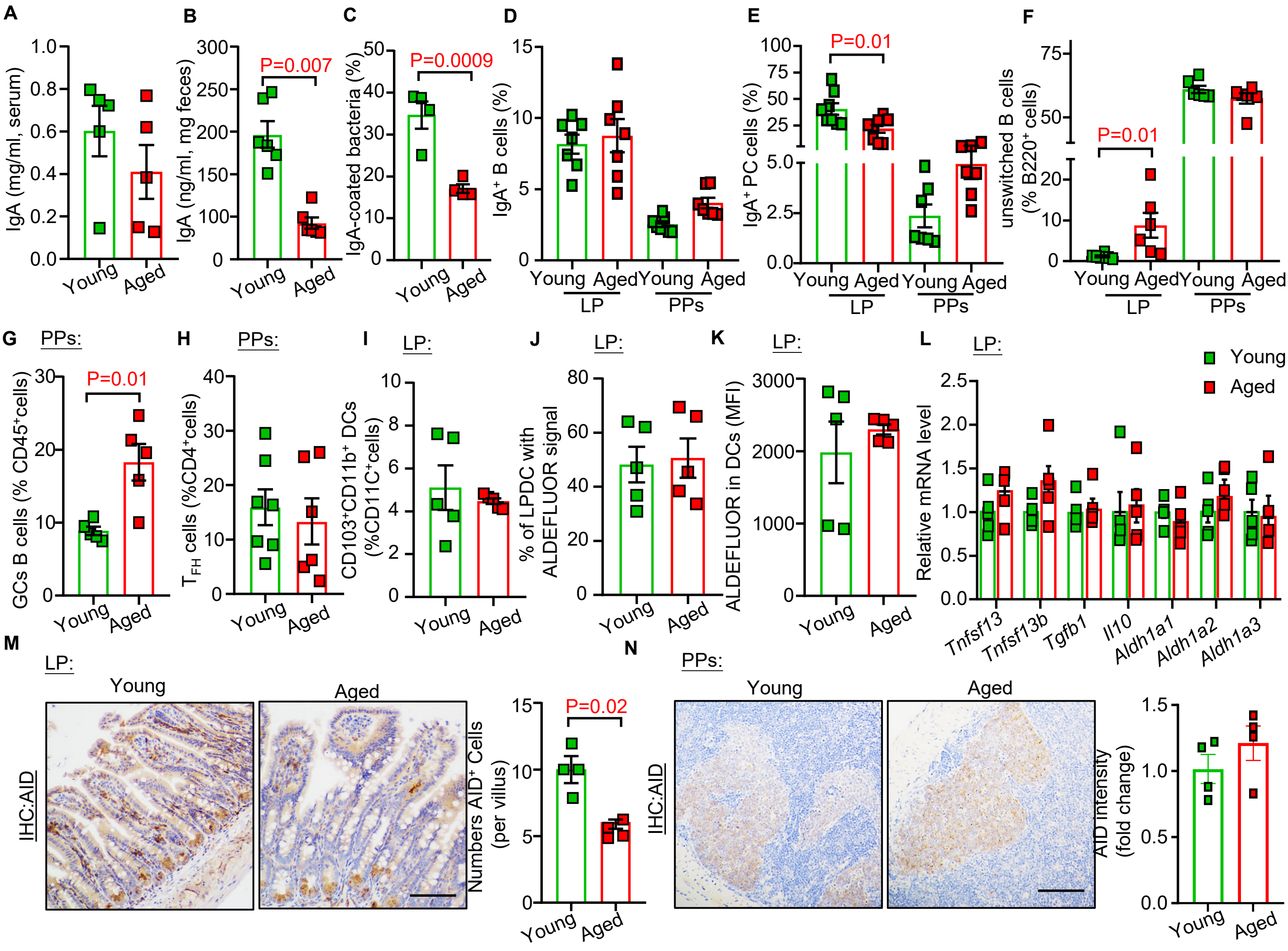

Supplementary Figure 23. Reduction of intestinal IgA production and IgA-producing B cell populations in aged mice. LP and PPs from 20-24-month-old (Aged) and 2-3-month-old (Young) C57BL/6J male mice were used. (**A**) IgA levels in the serum (*n*=5) and (**B**) feces (*n*=7). (**C**) Percentage of IgA-coated bacteria in the ileum (*n*=4). (**D-K**) Flow cytometry analysis for the frequencies of IgA$^+$ B cells (**D**), IgA$^+$ PC (**E**), unswitched B cells (**F**), GC B cells (**G**), TFH cells (**H**), LPDC (**I**), the frequency of ALDEFLUOR positive LPDC (**J**) and mean fluorescence intensity (MFI) of ALDEFLUOR in LPDC (**K**) (*n*=5-8). (**L**) mRNA levels related to the T-independent pathway IgA production, normalized with *gapdh* and expressed as fold change relative to the young controls (*n*=5). (**M-N**) Immunohistochemical staining of AID in the small intestine (**M**) and PPs (**N**). Scale bar: 100 µm (*n*=4). Representative images are shown. Data are displayed as mean ± SEM. Statistical analysis was performed using a two-tailed Mann-Whitney U test on **panel A, K, and N**, whereas the others were performed using a two-tailed Student's t-test.

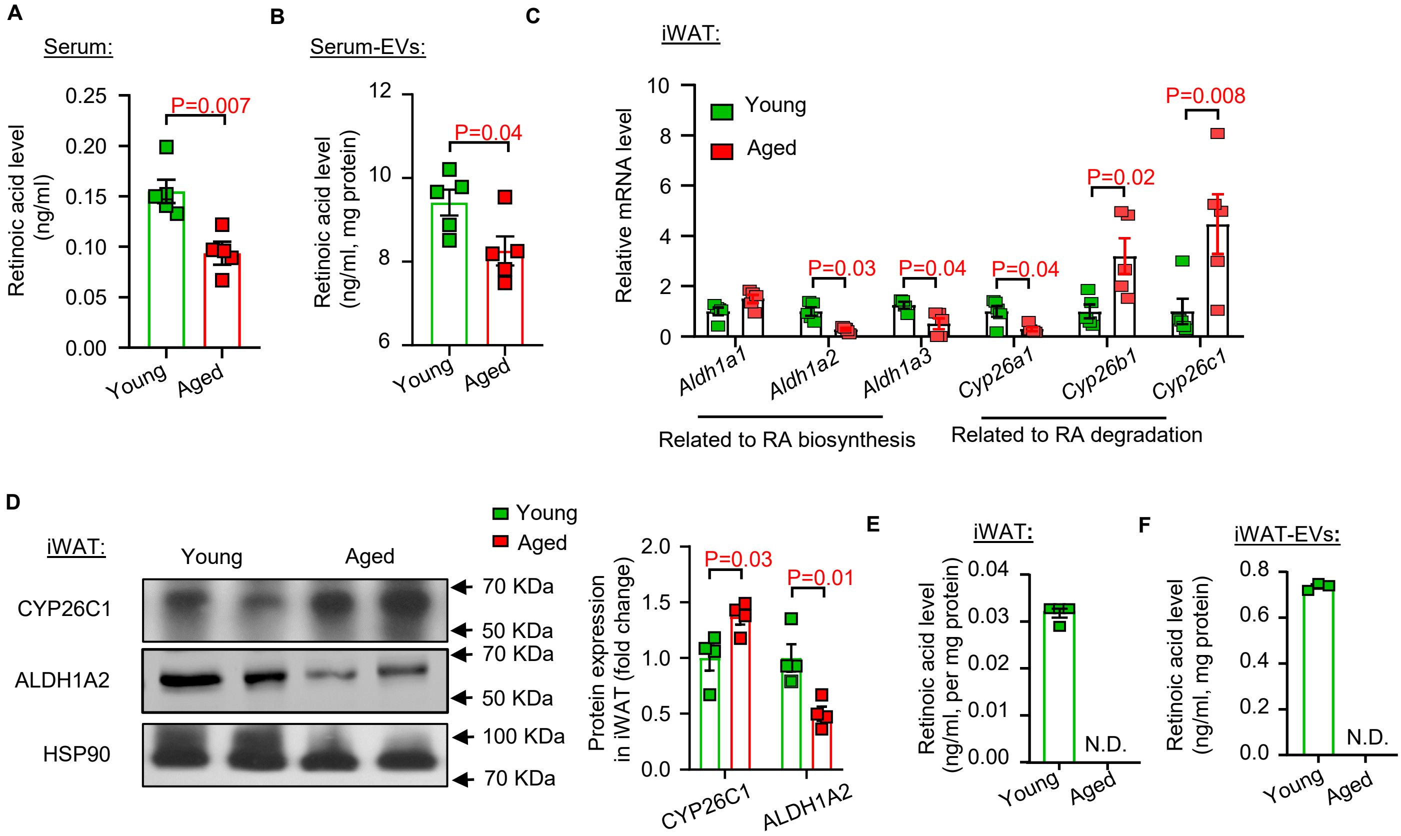

Supplementary Figure 24. Aged mice show defective RA biosynthesis in iWAT. (**A-B**) RA levels in serum, serum-extracted EVs (*n*=5). (**C**) QPCR analysis of genes related to RA biosynthesis and degradative gene level, expressed as fold change relative to the young controls (*n*=5). (**D**) Immunoblotting analysis of CYP26C1, ALDH1A2, and HSP90 in the iWAT. Representative images are shown. These expressions are normalized with HSP90 and presented as fold change relative to the young mice (*n*=4). (**E-F**) RA levels in the iWAT, and iWAT-EVs (*n*=4). Data are displayed as mean ± SEM. Statistical significance was determined using a two-tailed Student's t-test. N.D., Non-detectable.

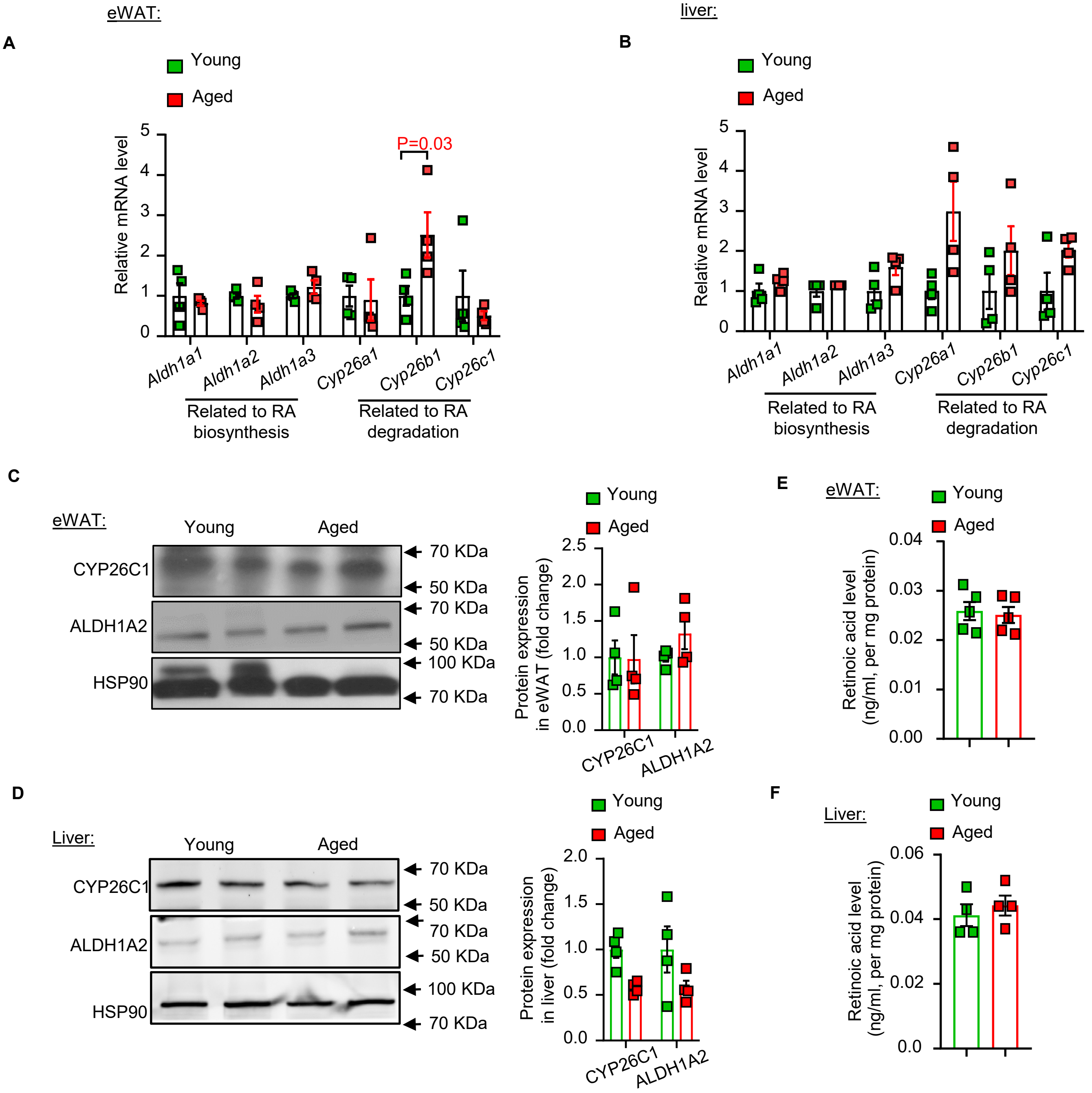

Supplementary Figure 25. Aging has no effect in the RA metabolism in eWAT and liver. (**A**) QPCR analysis of genes related to RA biosynthesis and degradative genes in the eWAT and (**B**) liver, expressed as fold change relative to the young controls (*n*=4). (**C**) Immunoblotting analysis of CYP26C1, ALDH1A2, and HSP90 in the eWAT and (**D**) liver. Representative images are shown. These expressions are normalized with HSP90 and presented as fold change relative to the young mice (*n*=4). (**E**) RA levels in eWAT and (**F**) liver (*n*=5). Data are displayed as mean ± SEM. Statistical analysis was performed using a two-tailed Mann-Whitney U test on **panel-A**, *Cyp26a1,* and *Cyp26c1* expression and **panel–B**, *Cyp26a1, Cyp26b1,* and *Cyp26c1* expression and **panels C-D**, whereas the others were performed using a two-tailed Student's t-test.

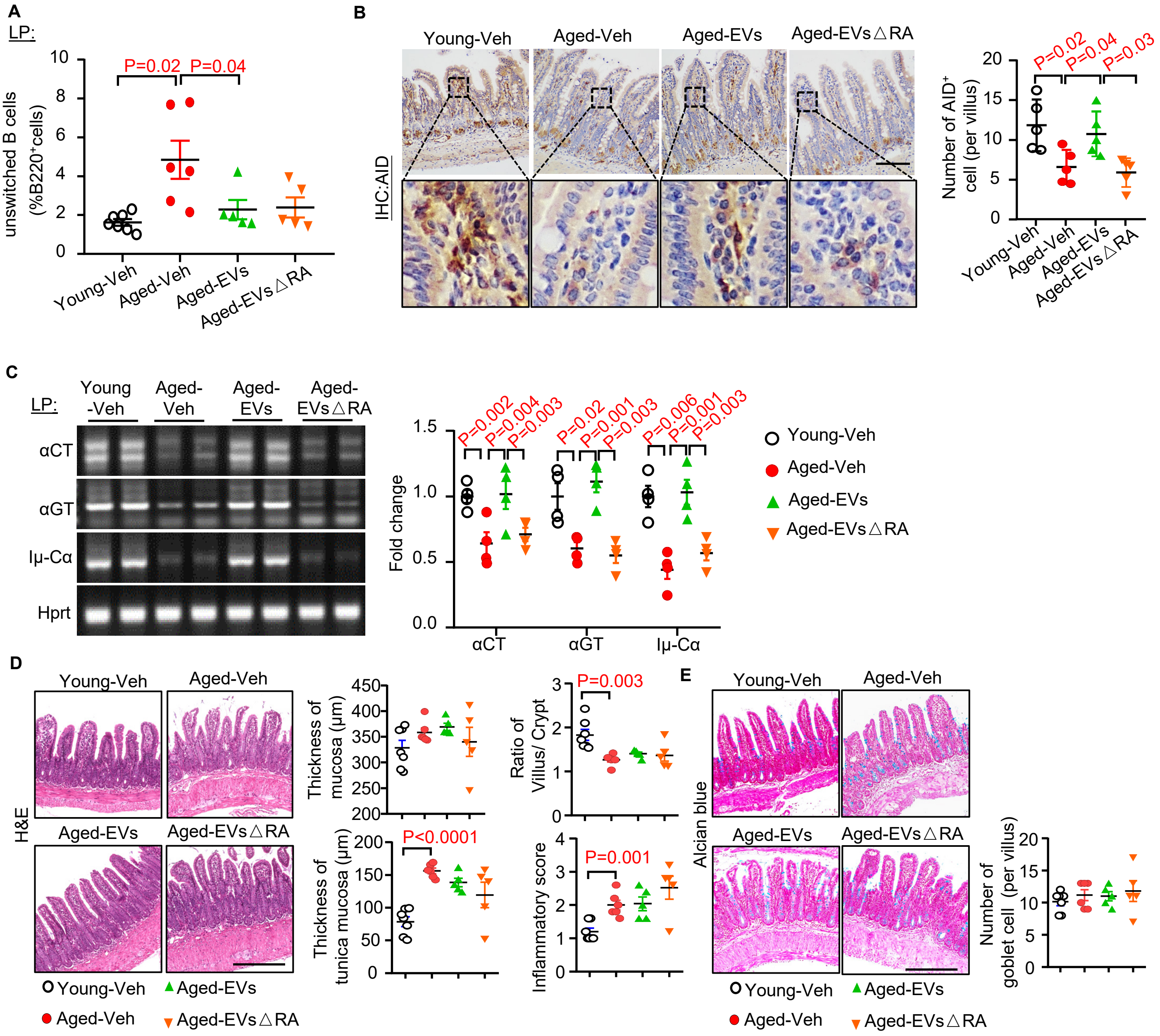

Supplementary Figure 26. RA in iWAT-EVs rejuvenates impaired IgA class switching in the LP of aged C57BL/6J mice. (**A**) Flow cytometric analysis of unswitched B cells in LP. All frequencies of cells were gated from CD45^{+}cells (n=5-7). (B) Immunohistochemical staining of AID in the small intestine, the right panel is the quantitative analysis (n=5). Scale bar:100μm. Representative images are shown. (**C**) Representative images of semi-quantitative PCR analysis of the class-switching markers in the LP and their quantitative analysis were shown in the right panel (n=4). (**D**) Representative images of the intestine by H&E staining and (**E**) Alcian blue staining in the intestine. The right panels show quantitative analysis of mucosa thickness, tunica mucosa thickness, villus-to-crypt ratio, and inflammatory score and number of goblet cells within the villus (n=5-7). Data are displayed as mean ± SEM. Statistical significance was determined using One-way ANOVA with Tukey's multiple comparisons test.

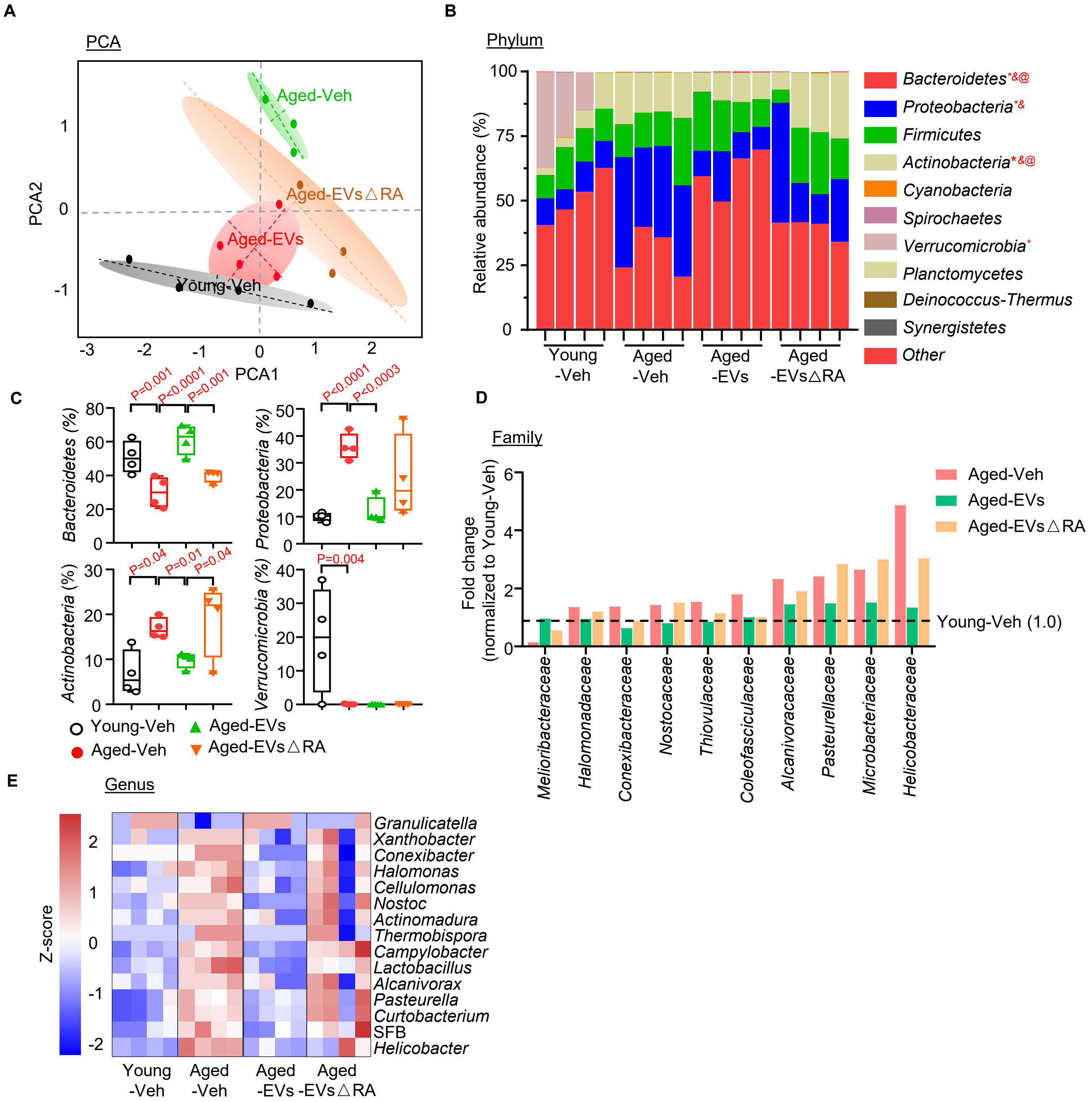

Supplementary Figure 27. iWAT-EVs improve microbiota dysbiosis in a RA-dependent manner during the aging process. Bacterial DNA was isolated from the mice cecum, followed by metagenomic sequencing (*n*=4). (**A**) PCA analysis of the gut microbiota. (**B**) Microbiota composition at the phylum level. (**C**) Significantly altered phyla across sample groups. (**D-E**) Microbiota composition at the family level and genus level that are significantly altered during aging (i.e. Young-Veh vs. Aged-Veh p-value<0.05), and the alteration could be reversed by iWAT-EVs treatment (i.e. Aged-Veh vs. Aged-EVs p-value<0.05) but not by EVs∆RA treatment (Aged-Veh vs. Aged-EVs∆RA p-value>0.05, actual p-value is presented in **Supplementary Table 6**). Data are displayed as mean ± SEM. Statistical significance was determined using One-way ANOVA with Tukey's multiple comparisons test.

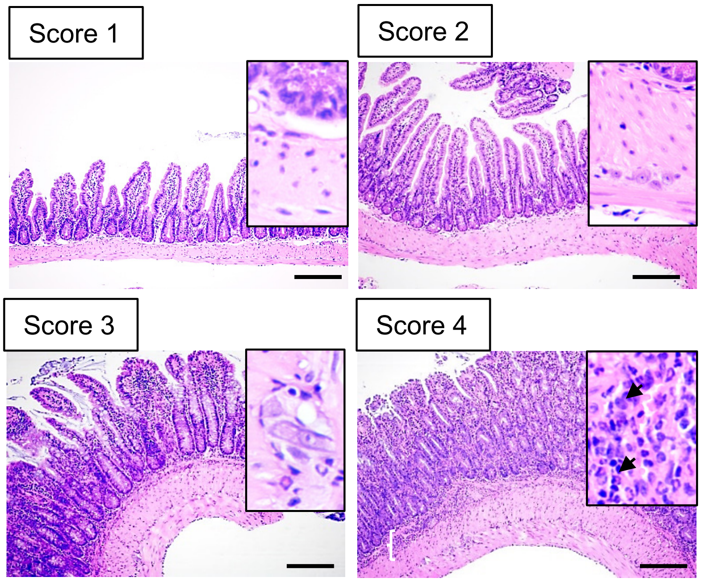

Inflammatory Score	Definition
1	Minimal: <10% leukocytes infiltrate in the lamina propria area; expansion of leukocytes in mucosal; no loss of surface epithelium
2	Mild:10-25% leukocytes infiltrate in the lamina propria area with scattered neutrophils; expansion of leukocytes in mucosal and submucosal; No loss of surface epithelium
3	Modest: 26-50% leukocytes infiltrate in the lamina propria area; expansion of leukocytes in mucosal and submucosal and transmural; loss lese of surface epithelium
4	Severe: >51% leukocytes infiltrate in the lamina propria area; expansion of leukocytes in mucosal and submucosal and transmural; damage of surface epithelium

Supplementary Figure 28. Assessment of intestinal inflammatory score. Definition of categories and general criteria in histomorphological scores for intestinal inflammation (score 1-4) in mouse models.

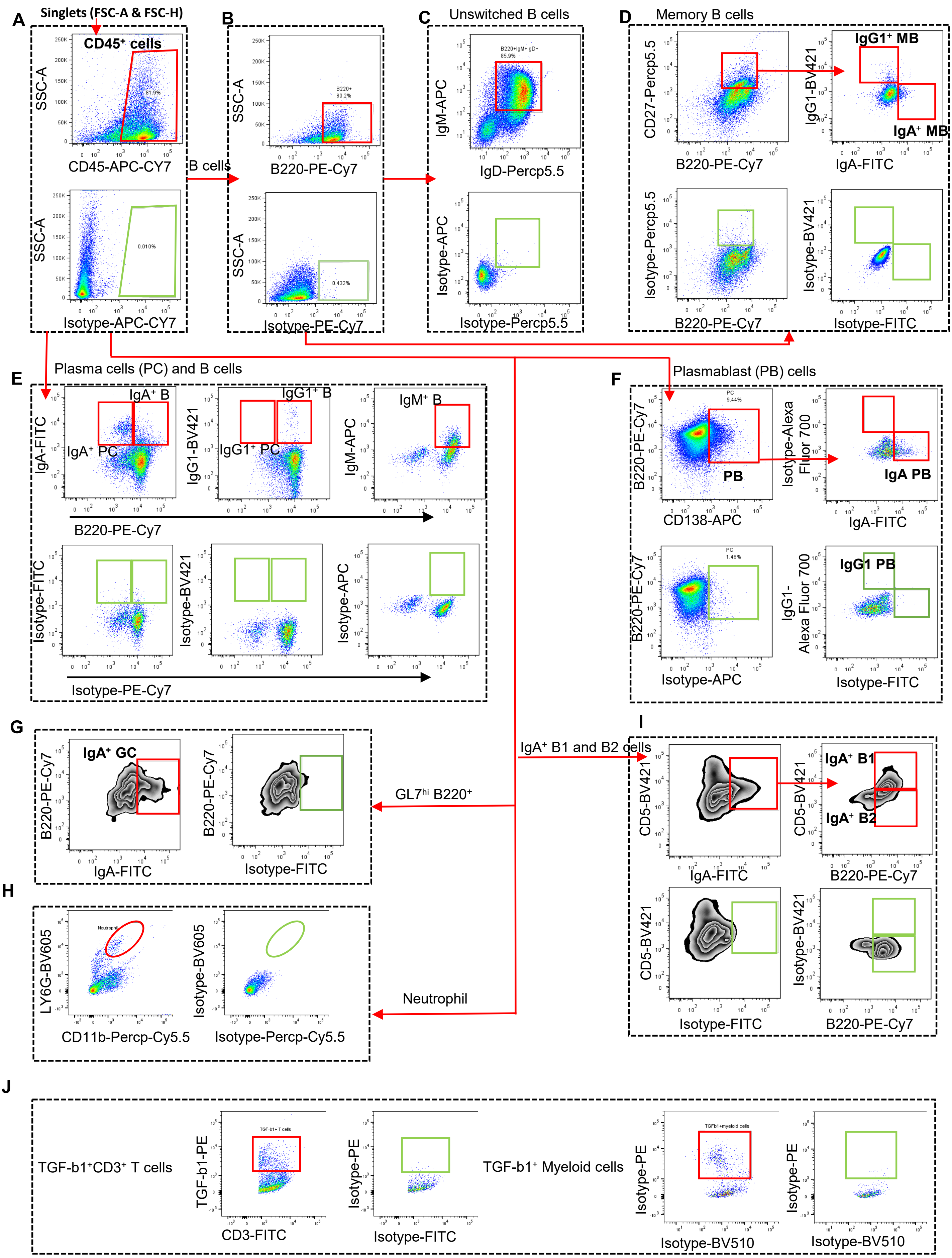
A
Singlets (FSC-A & FSC-H)
CD45⁺ cells
SSC-A
81.9%
CD45-APC-CY7
0.010%
Isotype-APC-CY7
B cells
B
B220+
80.2%
B220-PE-Cy7
0.432%
Isotype-PE-Cy7
C
Unswitched B cells
B220+IgM+IgD+
85.9%
IgM-APC
IgD-Percp5.5
Isotype-APC
Isotype-Percp5.5
D
Memory B cells
CD27-Percp5.5
B220-PE-Cy7
IgG1⁺ MB
IgA⁺ MB
IgG1-BV421
IgA-FITC
Isotype-Percp5.5
Isotype-BV421
Isotype-FITC
E
Plasma cells (PC) and B cells
IgA⁺ B
IgA⁺ PC
IgA-FITC
IgG1⁺ B
IgG1⁺ PC
IgG1-BV421
IgM⁺ B
IgM-APC
B220-PE-Cy7
Isotype-FITC
Isotype-BV421
Isotype-APC
Isotype-PE-Cy7
F
Plasmablast (PB) cells
PC
9.44%
B220-PE-Cy7
PB
CD138-APC
Isotype-Alexa Fluor 700
IgA PB
IgA-FITC
PC
1.46%
Isotype-APC
IgG1 PB
IgG1-Alexa Fluor 700
Isotype-FITC
G
IgA⁺ GC
B220-PE-Cy7
IgA-FITC
Isotype-FITC
GL7hi B220⁺
H
Neutrophil
LY6G-BV605
CD11b-Percp-Cy5.5
Isotype-BV605
Isotype-Percp-Cy5.5
Neutrophil
I
IgA⁺ B1 and B2 cells
CD5-BV421
IgA-FITC
IgA⁺ B1
IgA⁺ B2
B220-PE-Cy7
Isotype-FITC
Isotype-BV421
J
TGF-b1⁺CD3⁺ T cells
TGF-b1+ T cells
TGF-b1-PE
CD3-FITC
Isotype-PE
Isotype-FITC
TGF-b1⁺ Myeloid cells
TGFb1+myeloid cells
Isotype-BV510

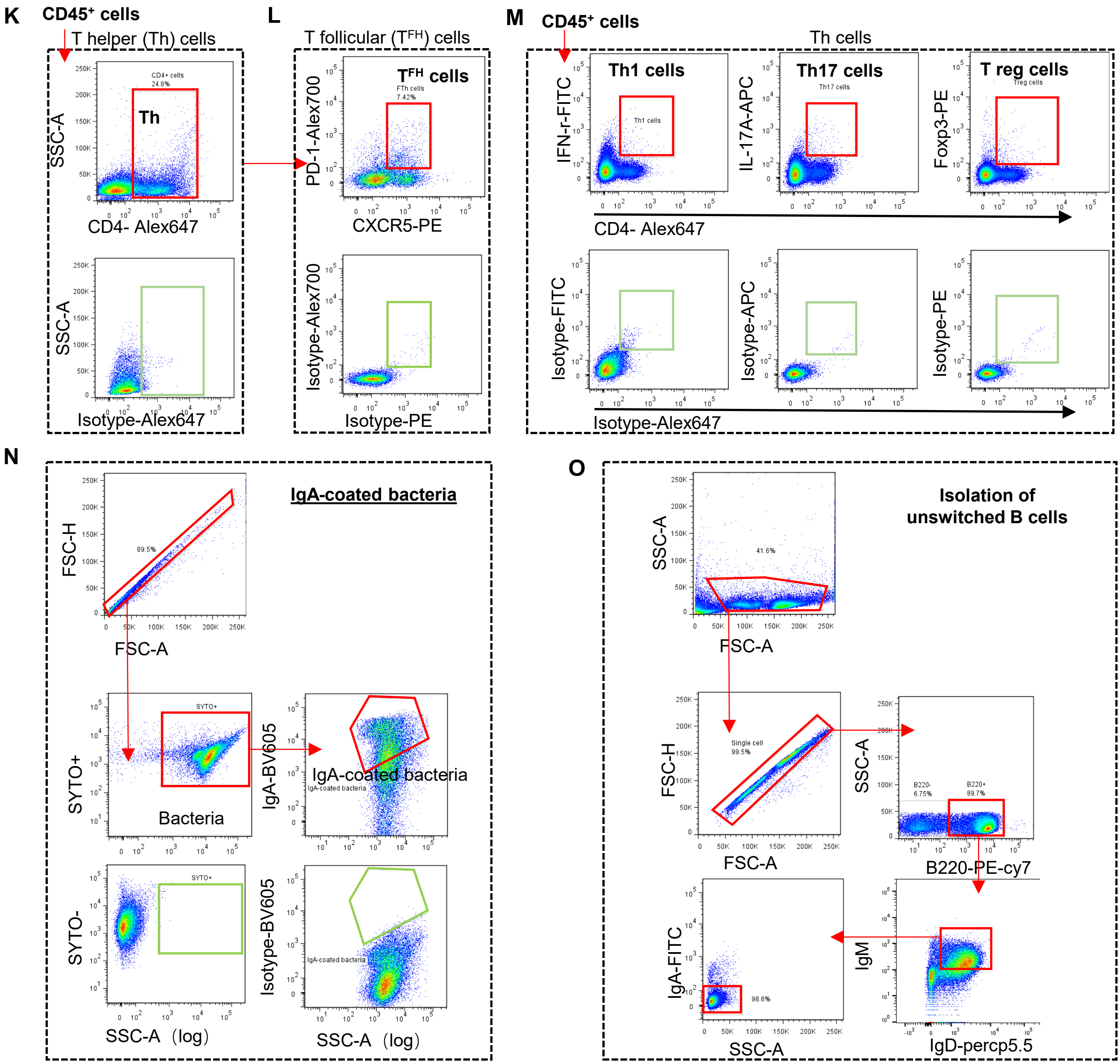

Supplementary Figure 29. Flow cytometry gating strategies. Total singlets were initially gated based on forward scatter area (FSC-A) and height (H). Subsequently, lymphocytes were gated using CD45 expression (**A**). The subsets of interest were then gated as follows: B220$^+$ B cells (**B**), B220$^+$ IgM$^+$ IgD$^+$ unswitched B cells (**C**), CD27$^+$B220+ IgA$^+$ and IgG1$^+$ memory B cells (**D**), IgA$^+$B220$^-$, IgG1$^+$B220$^-$ plasma cells, and IgA$^+$, IgG1$^+$, and IgM$^+$B220$^+$ B cells (**E**), CD138$^+$B220^{lo} IgA$^+$ plasmablasts and IgG1$^+$ plasmablasts (**F**), GL7^{hi}B220$^+$ IgA germinal center B cells (**G**), CD11b$^+$Ly6G$^+$ neutrophil (**H**), IgA$^+$B220$^+$ CD5$^+$ B cells and CD5$^-$ B2 cells, CD4+ T helper cells (**I**), TGF-b1$^+$CD3$^+$ T cells and TGF-b1$^+$CD11c$^+$CD11b$^+$ Myeloid cells (**J**), CD4$^+$ T helper cells (Th)(**K**), CD4$^+$ CXCR5$^+$ PD-1$^+$ T follicular cells (**L**), CD4$^+$ IFN-r$^+$ Th1 cells, CD4$^+$ IL-17A$^+$ Th17 cells, and CD4$^+$ Foxp3$^+$ T regulatory cells (**M**). The IgA-coated bacteria were gated based on SYTO$^+$ population (**N**). B220$^+$ gated IgM$^+$IgD$^+$IgA$^-$ unswitched B cells (**O**). The corresponding isotype controls are shown.

Mouse model of iWAT removal					
	Group	Week-0	Week-2	Week-4	Week-5
**Body weight (g)**	iSham	25.13±1.47	25.18±1.59	26.09±1.65	
	iFR	26.15±1.56	25.88±1.42	27.13±1.66	
**Food intake (g/mouse, day)**	iSham	3.22±0.34	4.04±0.73	4.50±0.35	
	iFR	3.26±0.29	4.61±0.18	5.06±0.20	
**Blood Glucose (mmol/ml)**	iSham	7.89±0.64	8.71±0.55	9.18±1.31	
	iFR	8.11±0.59	9.24±1.43	9.86±094	
**Triglyceride (mg/dL)**	iSham				66.2±3.12
	iFR				77.3±11.4
**ALT (U/L)**	iSham				18.2±2.71
	iFR				21.1±1.62
**AST (U/L)**	iSham				33.3±6.1
	iFR				41.8±5.7
**Adiponectin (μg/ml)**	iSham				24.0±4.7
	iFR				18.9±1.6
**Leptin (μg/ml)**	iSham				1.98±0.97
	iFR				2.68±0.61
**BAFF (ng/ml)**	iSham				4.107±0.795
	iFR				3.754±0.560
**TGFB1 (pg/ml)**	iSham				54.45±10.54
	iFR				71.62±15.27*
**Serum-EVs retinoic acid (ng/ml, mg protein)**	iSham				9.75 ±0.5
	iFR				8.33±0.5*
**Serum IgA (mg/ml)**	iSham				0.50±0.13
	iFR				0.53±0.27
**Fecal IgM (ng/ml, g feces)**	iSham				54.44±24.38
	iFR				46.29±19.77

Supplementary Table 1. **Metabolic parameters and circulating biomarkers in the iSham mice and iFR mice.** Mouse body weight and food intake were monitored at 0, 2, and 4 week post-iWAT removal surgery. Serum was collected under fed conditions at different time points, followed by measurement of glucose, triglyceride, adiponectin, leptin, alanine transaminase (ALT), aspartate aminotransferase (AST), B-cell activating factor (BAFF), TGFB1, and RA in the extracellular vesicles (EVs). Serum IgA and fecal IgM at 5-week post-iWAT removal surgery. *n*=8 per group. Data are displayed as mean ± SEM. Statistical analysis was performed by a two-tailed Mann-Whitney U test on TGFB1 measurement, and others were performed using a two-tailed Student's t-test. Significant P-values: $^*p<0.05$.

Mouse model of eWAT removal					
	Group	Week-0	Week-2	Week-4	Week-5
**Body weight (g)**	Sham	28.7±1.9	29.0±1.9	29.0±3.2	
	eFR	27.3±2.8	26.1±2.5*	26.1±2.7*	
**Food intake (g/mouse, day)**	Sham	4.2±0.2	5.2±0.1	4.2±0.1	
	eFR	4.1±0.6	4.8±0.5	4.5±0.5	
**Blood Glucose (mmol/ml)**	Sham	8.8±0.8	8.0±0.7	8.1±0.6	
	eFR	8.3±0.7	7.1±0.7*	7.0±0.5*	
**Triglyceride (mg/dL)**	Sham				115.2±20.6
	eFR				104.5±24.4
**ALT (U/L) (U/L)**	Sham				18.8±6.9
	eFR				18.7±4.9
**LPS (EU/L)**	Sham				0.23±0.03
	eFR				0.27±0.05
**Retinoic acid (ng/ml)**	Sham				0.32±0.1
	eFR				0.32±0.04
**Serum IgA (mg/ml)**	Sham				0.32±0.2
	eFR				0.52±0.3
**Fecal IgM (ng/ml, g feces)**	Sham				49.7±17.7
	eFR				43.3±19.1

Supplementary Table 2. Metabolic parameters and circulating biomarkers in the iSham mice and eFR mice. Mouse body weight and food intake were monitored at 0, 2, and 4 week post-eWAT removal surgery. Serums were collected under fed conditions at different time points, followed by measurement of glucose, triglyceride, LPS, alanine transaminase (ALT), RA, and IgA. Fecal IgM level. *n*=6 per group. Data are displayed as mean ± SEM. Statistical significance was determined using a two-tailed Student's t-test. Significant P-values: *p<0.05.

iWAT-EVs

	PG.Genes	PG.ProteinDescriptions	Relative abundance			
			iWAT-EVs(1)	iWAT-EVs(2)	iWAT-EVs(3)	iWAT-EVs(4)
EVs protein	CD63	CD63 antigen	206.9	151.2	172.0	211.0
	CD9	CD9 antigen	175.0	165.8	155.0	242.6
	CD81	CD81 antigen	126.2	121.9	120.6	134.4
	GAPDH	Glyceraldehyde-3-phosphate dehydrogenase	9046.1	8839.4	8747.8	8699.9
	ALDOA	Fructose-bisphosphate aldolase A	6097.6	5988.2	6238.8	6179.4
	PGK1	Phosphoglycerate kinase 1	4702.3	4719.8	4817.9	4925.2
Mitochondrial protein	TOM20	Translocase of outer mitochondrial membrane 20	N.D.	N.D.	N.D.	N.D.
Cellular protein	GM130	Golgi matrix protein GM130	N.D.	N.D.	N.D.	N.D.
IgA class switching protein	LTF	Lactotransferrin	7806.9	2960.4	2852.4	2844.7
	TGFB1	Transforming growth factor β-1 proprotein	60.5	58.6	61.9	46.1

Supplementary Table 3. Proteins in the iWAT-EVs. iWAT-EVs were isolated from iWAT of 10-12-week-old C57BL/6J mice as described in method. Extracted protein from iWAT-EVs was used for proteomics analysis. The value is presented as relative abundance. N.D., Non-detectable.

AAV-*Cyp26c1* iWAT-injected-mouse model

	Group	Week 0	Week 3	Week 4
**Body weight (g)**	AAV-*GFP*	24.3±1.2	25.3±1.1	26.1±1.3
	AAV-*Cyp26c1*	23.3±1.4	24.5±1.7	24.9±2.0
**Food intake (g/mouse, day)**	AAV-*GFP*	3.8±0.6	3.7±0.4	4±0.7
	AAV-*Cyp26c1*	3.8±0.4	3.8±0.9	4±0.8
**Triglyceride (mg/dL)**	AAV-*GFP*			59.7±14.9
	AAV-*Cyp26c1*			55.8±2.4
**ALT (U/L) (U/L)**	AAV-*GFP*			21.8±3.3
	AAV-*Cyp26c1*			24.2±1.9
**Serum IgA (mg/ml)**	AAV-*GFP*			0.7±0.5
	AAV-*Cyp26c1*			0.9±0.3
**Fecal IgM (ng/ml, g feces)**	AAV-*GFP*			56.2±32.2
	AAV-*Cyp26c1*			49.8±22.7
**Retinoic acid in serum (ng/ml)**	AAV-*GFP*			0.26±0.012
	AAV-*Cyp26c1*			0.16±0.025*
**Retinoic acid in serum-EVs (ng/ml,mg protein)**	AAV-*GFP*			6.45±0.57
	AAV-*Cyp26c1*			3.58±2.03*
**Retinoic acid in eWAT-EVs (ng/ml,mg protein)**	AAV-*GFP*			1.15±0.18
	AAV-*Cyp26c1*			1.01±0.19
**Retinoic acid in liver-EVs (ng/ml,mg protein)**	AAV-*GFP*			2.10±1.20
	AAV-*Cyp26c1*			3.50±1.45

Supplementary Table 4. **Metabolic parameters and circulating biomarkers in the AAV-*Cyp26c1* iWAT injected mice.** Mouse body weight and food intake were monitored at week 0, 3, and 4 post-AAV injection. Serum triglyceride, alanine transaminase (ALT), and IgA levels were measured. Fecal IgM was measured under the fed condition. RA in serum, serum-EVs, eWAT-EVs, and liver-EVs were measured by LC-MS/MS. *n*=5 per group. Data are displayed as mean ± SEM. Statistical analysis was performed by a two-tailed Mann-Whitney U test on TGFB1 measurement, and others were performed using a two-tailed Student's t-test. Significant P-values: *p<0.05.

Bacteria	Average AAV-*GFP*	Average AAV-*Cyp26c1*	Fold change (AAV-*Cyp26c1*/AAV-*GFP*)	P value
f__*Coleofasciculaceae*	0.0000	0.0000	0.366	0.024
f__*Helicobacteraceae*	0.0036	0.0046	1.265	0.002
f__*Melioribacteraceae*	0.0001	0.0002	2.229	0.005
g__*Curtobacterium*	0.0000	0.0001	3.841	0.050
g__*Lactobacillus*	0.0023	0.0033	1.423	1.9458E-05
g__*SFB*	0.0057	0.0088	1.539	0.013

Supplementary Table 5. Ageing-induced gut microbiota are reversed by iWAT-EVs treatment in a RA-dependent manner. Metagenomic analysis of cecal microbiota from the mice post-injection of AAV-*GFP* and AAV-*Cyp26c1* into iWAT for 4 weeks. The table lists significantly changed microbiota at the family level (f) and genus level (g), consistent with changes observed in the aging mouse model. These changes are partially reversed by iWAT-EVs treatment, through a RA-dependent mechanism. *n*=5 per group. Data are displayed as averaged relative bacterial abundance. Statistical analysis was performed by a two-tailed Student's t-test. Significant P-values: *p<0.05.

Figure1 K.	iFR vs. iSham
Taxonomy	P value
Lachnospiraceae	0.0355
Muribaculaceae	0.0469
Clostridiales_vadinBB60_group	0.0135
Burkholderiaceae	0.0199
Bacteroidaceae	0.0068
Peptococcaceae	0.0399
Enterobacteriaceae	0.0236
Deferribacteraceae	0.0297
Rikenellaceae	0.0002

Figure1 L.	iFR vs. iSham
Taxonomy	P value
NK3B31_group	0.0412
Lachnoclostridium	0.0471
Rikenella	0.0428
uncultured	0.0412

Taxonomy	P value
Ruminococcaceae_NK4A214_group	0.0146
[Eubacterium]_xylanophilum_group	0.0045
Bacteroides	0.0365
Romboutsia	0.0328
uncultured	0.0098
Bilophila	0.0396
Prevotellaceae_UCG-001	0.0232
Escherichia-Shigella	0.0204
Ruminococcaceae_UCG-013	0.0423
Parasutterella	0.0433
Ruminiclostridium_6	0.0463
UBA1819	0.0028
Mucispirillum	0.0130
GCA-900066225	0.0190
Alistipes	0.0047
Anaerotruncus	0.0051
Candidatus_SFB	0.0074

**Supplementary Figure 7 D.**	**iFR vs. iSham**
Phylum	**P value**
Bacteroidota	0.0103
Cyanobacteria	0.0071
Actinobacteriota	0.0102
Acidobacteriota	0.0345

**Supplementary Figure 7 E.**	**iFR vs. iSham**
Family	**P value**
Lachnospiraceae	0.0166
Firmicutes_unclassified	0.0328
Prevotellaceae	0.0000
Ruminococcaceae	0.0136
Desulfovibrionaceae	0.0237
Bacteroidaceae	0.0371
Peptococcaceae	0.0004
Gastranaerophilales_unclassified	0.0380
Acholeplasmataceae	0.0011
Chloroplast_unclassified	0.0133
Clostridia_UCG-014_unclassified	0.0413
Pseudomonadaceae	0.0265
Clostridiales_Family_XIV._Incertae_Sedis	0.0117
Peptostreptococcaceae	0.0037
Tannerellaceae	0.0031
Saccharimonadaceae	0.0457
Aeromonadaceae	0.0433
Bifidobacteriaceae	0.0192
Sutterellaceae	0.0000
Moraxellaceae	0.0232
Eggerthellaceae	0.0364
Monoglobaceae	0.0143
Paenibacillaceae	0.0429
Acidaminococcaceae	0.0000
Kaistiaceae	0.0464

**Supplementary Figure 7 F.**	**iFR vs. iSham**
Genus	**P value**
Firmicutes_unclassified	0.0328
Muribaculaceae_unclassified	0.0379
Tyzzerella	0.0000
Prevotellaceae_UCG-001	0.0000
Muribaculum	0.0044
Acetatifactor	0.0072

**Supplementary Figure 7 F.**	**iFR vs. iSham**
Genus	**P value**
Oscillospiraceae_unclassified	0.0132
Kineothrix	0.0008
Desulfovibrio	0.0164
Rikenella	0.0019
Paramuribaculum	0.0424
Bacteroides	0.0401
ASF356	0.0075
Peptococcaceae_unclassified	0.0004
Bilophila	0.0003
A2	0.0132
Gastranaerophilales_unclassified	0.0380
Anaeroplasma	0.0011
UCG-003	0.0021
GCA-900066575	0.0245
Chloroplast_unclassified	0.0133
Rikenellaceae_RC9_gut_group	0.0001
Tuzzerella	0.0000
Clostridium_sensu_stricto_1	0.0000
UCG-009	0.0011
Faecalibacterium	0.0007
Ligilactobacillus	0.0444
Clostridia_UCG-014_unclassified	0.0413
Pseudomonas	0.0265
Clostridiales_Family_XIV._Incertae_Sedis_unclassified	0.0117
Lachnospiraceae_FCS020_group	0.0197
Parabacteroides	0.0027
Duncaniella	0.0001
Candidatus_Saccharimonas	0.0457
Romboutsia	0.0069
Aeromonas	0.0433
Bifidobacterium	0.0192
Parasutterella	0.0009
Fournierella	0.0004
Erysipelotrichaceae_unclassified	0.0262
Christensenellaceae_unclassified	0.0088
Monoglobus	0.0143
Paenibacillus	0.0429
Phascolarctobacterium	0.0000
Paludicola	0.0185
Sutterella	0.0185
Kaistia	0.0464

Supplementary Figure 22 C.	AAV-Cyp26c1 vs. AAV-GFP
Taxonomy	**P value**
p__Verrucomicrobiota	0.0002
p__Actinomycetota	0.0050
p__Spirochaetota	0.0009
p__Myxococcota	0.0431
p__Candidatus_Melainabacteria	0.0136
p__Synergistota	0.0041
p__Chloroflexota	0.0117
p__Euryarchaeota	0.0002
p__Hofneiviricota	0.0269
p__Fibrobacterota	0.0168
p__Cyanobacteriota	0.0115
p__Candidatus_Thermoplasmatota	0.0230
p__Thermotogota	0.0170
p__Elusimicrobiota	0.0436
p__Ignavibacteriota	0.0173

Supplementary Figure 22 E.	AAV-Cyp26c1 vs. AAV-GFP
Taxonomy	**P value**
g__Oscillibacter	0.0047
g__Muribaculum	0.0009
g__Pseudoflavonifractor	0.0405
g__Bacillota_unclassified	0.0024
g__Acetatifactor	0.0284
g__Paramuribaculum	0.0418
g__Clostridium	0.0046
g__Heminiphilus	0.0000
g__Alistipes	0.0004
g__Akkermansia	0.0002

Supplementary Figure 22 D.	AAV-Cyp26c1 vs. AAV-GFP
Taxonomy	**P value**
f__Eubacteriales_unclassified	0.0267
f__Clostridiaceae	0.0104
f__Bacillota_unclassified	0.0023
f__Rikenellaceae	0.0004
f__Akkermansiaceae	0.0002
f__Coriobacteriaceae	0.0051
f__Odoribacteraceae	0.0211
f__Barnesiellaceae	0.0486
f__Erysipelotrichaceae	0.0077
f__Tannerellaceae	0.0043

**Supplementary Figure 27 D.**			**P value**	
Taxonomy	Young vs Aged-Veh	Aged-Veh vs Aged-EV	Aged-EV△RA vs Aged-EV	Aged-EV△RA vs Aged-Veh
Melioribacteraceae	0.0115	0.0019	0.2492	0.1954
Halomonadaceae	0.0439	0.0028	0.3362	0.5915
Conexibacteraceae	0.0240	0.0054	0.4881	0.1901
Nostocaceae	0.0265	0.0001	0.0744	0.8053
Thiovulaceae	0.0004	0.0001	0.1135	0.0401
Coleofasciculaceae	0.0300	0.0300	1.0000	0.1135
Alcanivoracaceae	0.0058	0.0047	0.3352	0.3559
Pasteurellaceae	0.0281	0.0172	0.0867	0.5595
Microbacteriaceae	0.0111	0.0037	0.0708	0.6222
Helicobacteraceae	0.0001	0.0005	0.2304	0.1994

**Supplementary Figure 27 E.**			**P value**	
Taxonomy	Young vs Aged-Veh	Aged-Veh vs Aged-EV	Aged-EV△RA vs Aged-Veh	Aged-EV△RA vs Aged-Veh
Granulicatella	0.0300	0.0300	0.2070	0.2070
Xanthobacter	0.0240	0.0498	0.7049	0.7049
Conexibacter	0.0240	0.0054	0.1901	0.1901
Halomonas	0.0336	0.0012	0.4852	0.4852
Cellulomonas	0.0447	0.0320	0.3600	0.3600
Nostoc	0.0091	0.0000	0.8634	0.8634
Actinomadura	0.0038	0.0447	0.7216	0.7216
Thermobispora	0.0240	0.0240	0.3903	0.3903
Campylobacter	0.0010	0.0002	0.4078	0.4078
Lactobacillus	0.0042	0.0022	0.0643	0.0643
Alcanivorax	0.0045	0.0033	0.3045	0.3045
Pasteurella	0.0256	0.0175	0.5605	0.5605
Curtobacterium	0.0111	0.0038	0.6214	0.6214
SFB	0.0240	0.0170	0.3559	0.3559
Helicobacter	0.0001	0.0005	0.1993	0.1993

Supplementary Table 6. The actual p-value is represented as the relative bacterial abundance
