# Supplementary material for: Subcutaneous white adipose tissue–derived extracellular vesicles maintain intestinal homeostasis via IgA biosynthesis in aging mice
Source: J Clin Invest. 2025 Nov 17;135(22):e188947. doi: 10.1172/JCI188947 (PMC12618071; doi:10.1172/JCI188947)

Figure3. J

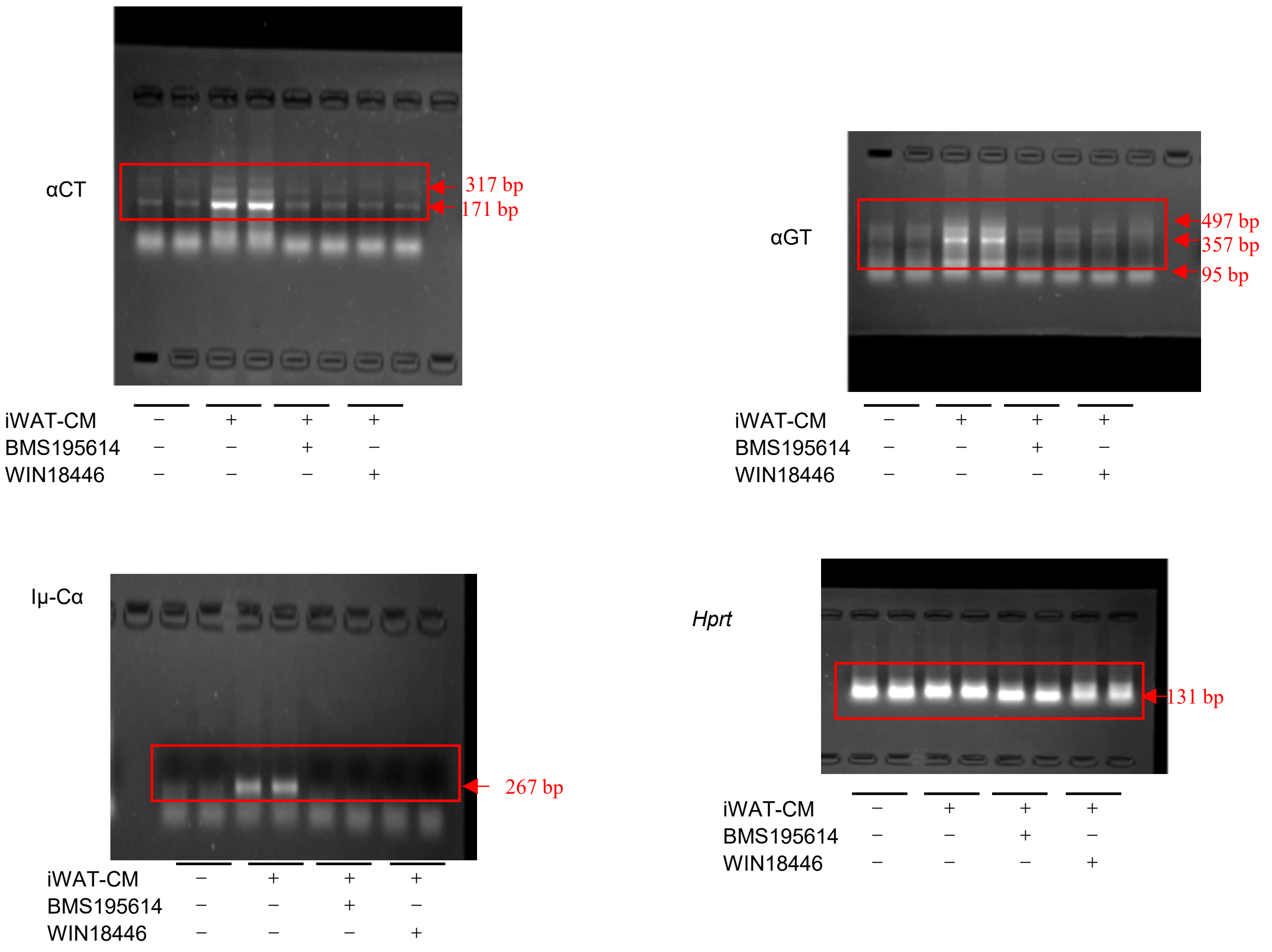
αCT
317 bp
171 bp
iWAT-CM − + + +
BMS195614 − − + −
WIN18446 − − − +
αGT
497 bp
357 bp
95 bp
iWAT-CM − + + +
BMS195614 − − + −
WIN18446 − − − +
Iμ-Cα
267 bp
iWAT-CM − + + +
BMS195614 − − + −
WIN18446 − − − +
Hprt
131 bp
iWAT-CM − + + +
BMS195614 − − + −
WIN18446 − − − +

Figure7. A

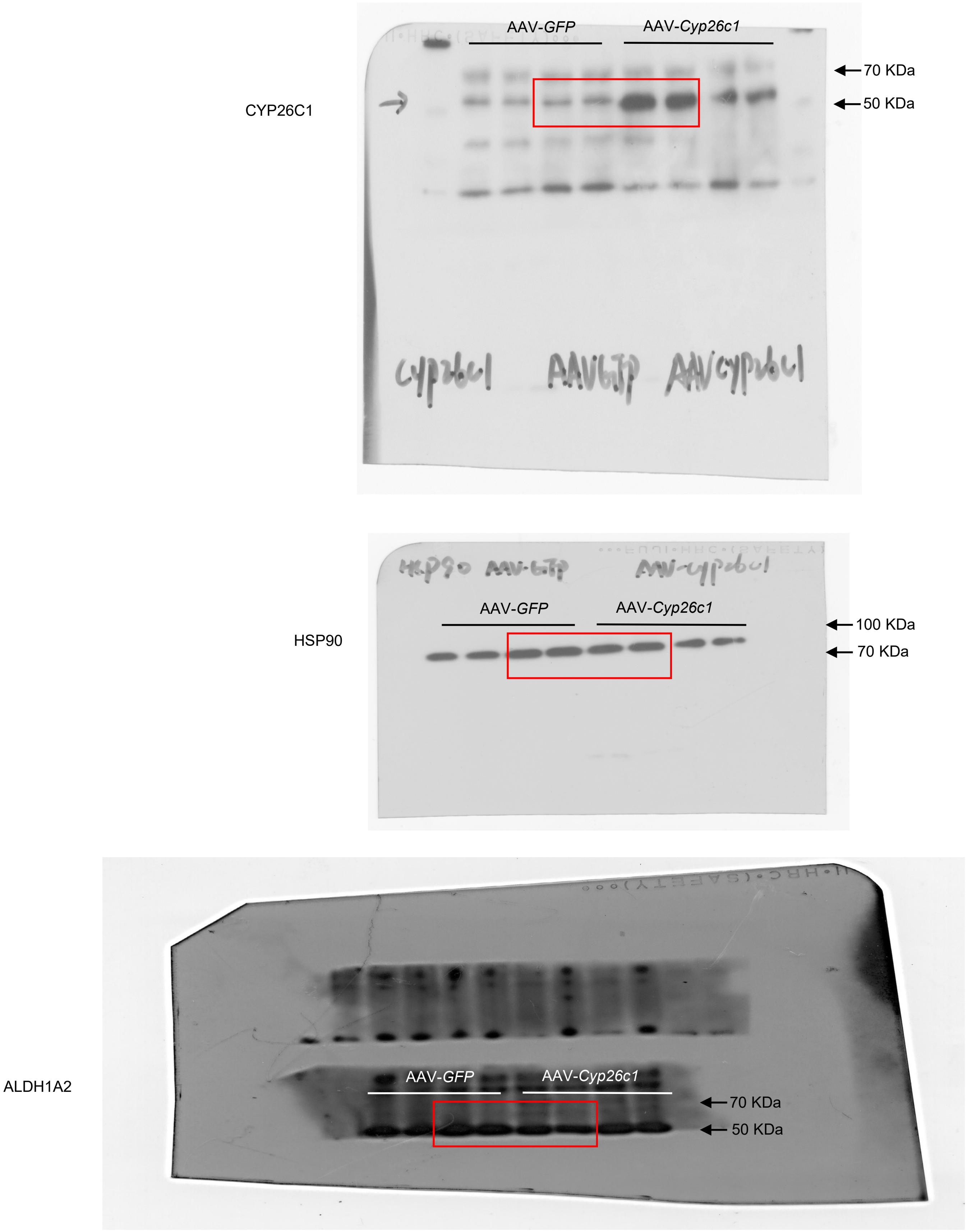
CYP26C1
AAV-*GFP*
AAV-*Cyp26c1*
70 KDa
50 KDa
HSP90
AAV-*GFP*
AAV-*Cyp26c1*
100 KDa
70 KDa
ALDH1A2
AAV-*GFP*
AAV-*Cyp26c1*
70 KDa
50 KDa

Supplementary Figure 1F

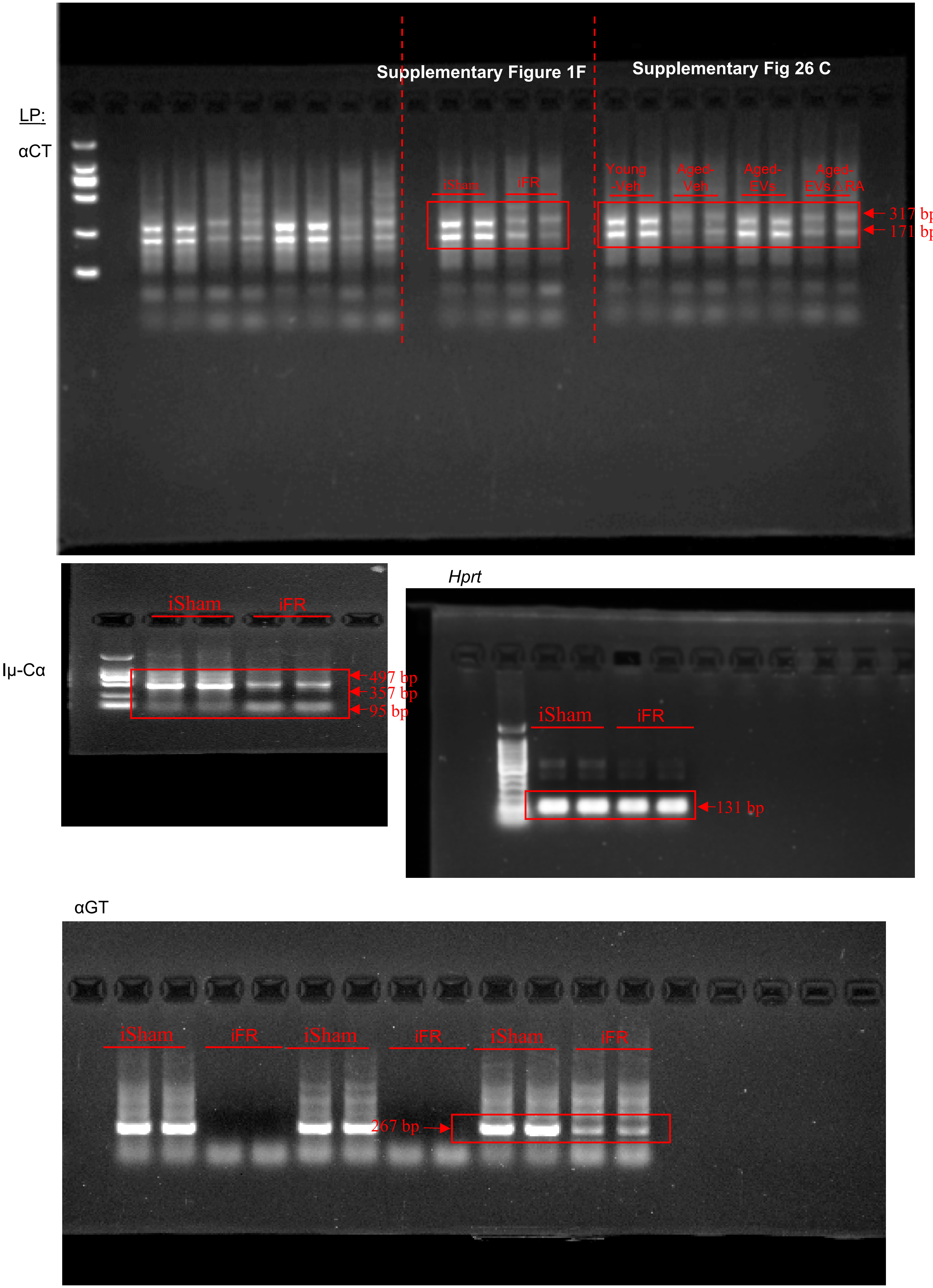

Supplementary Fig 12E

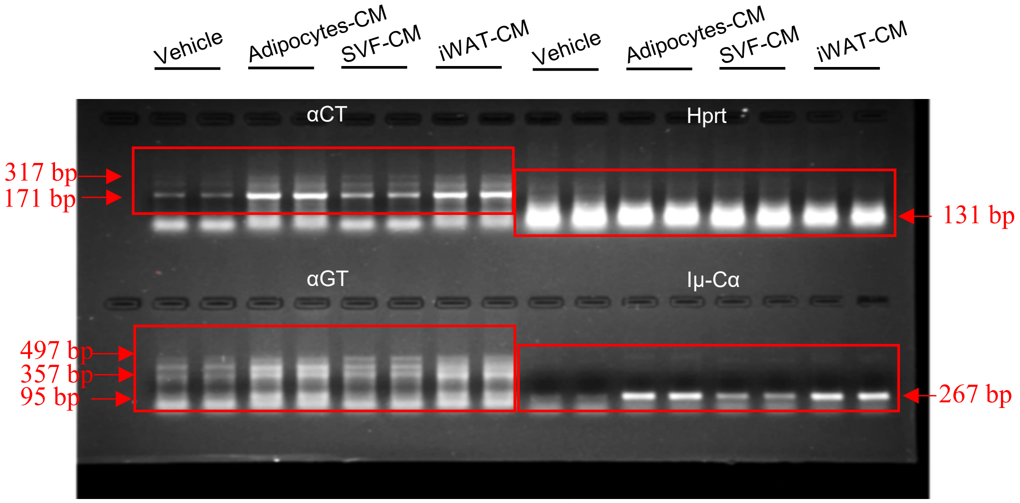

Supplementary Fig 14A

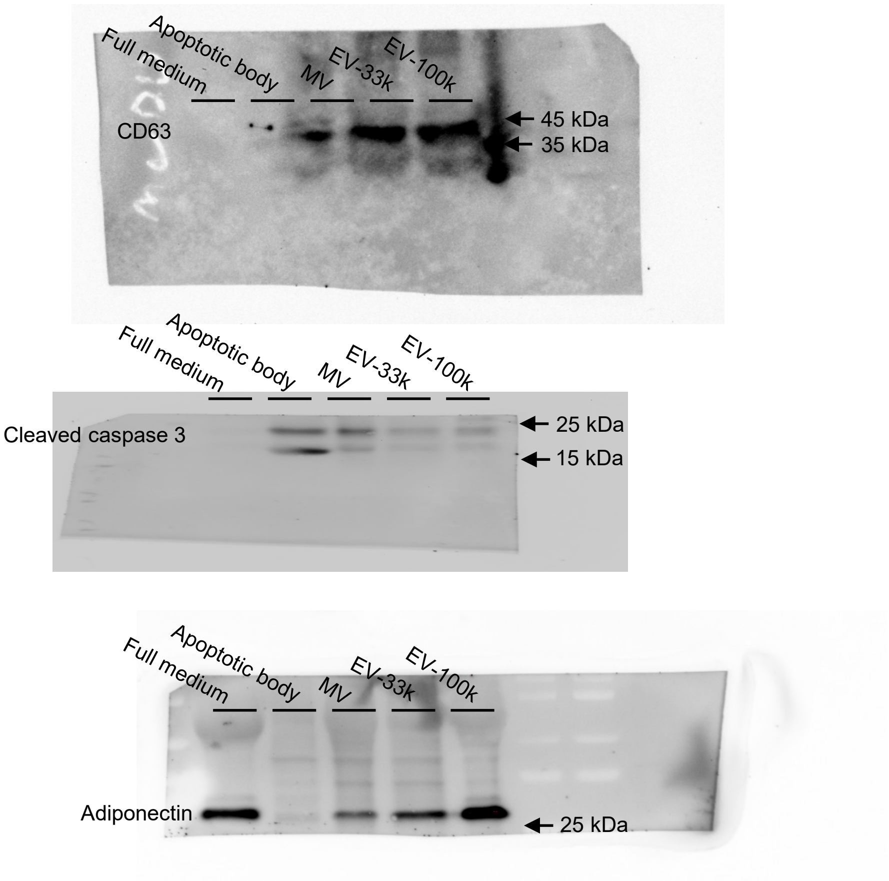

Supplementary Fig 14G

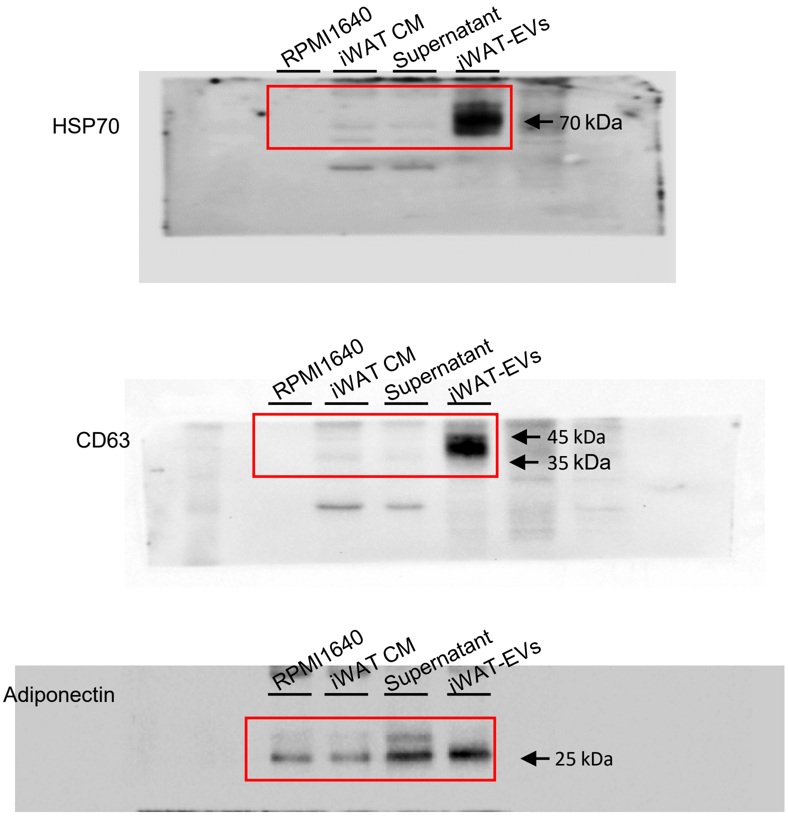

Supplementary Fig 16B

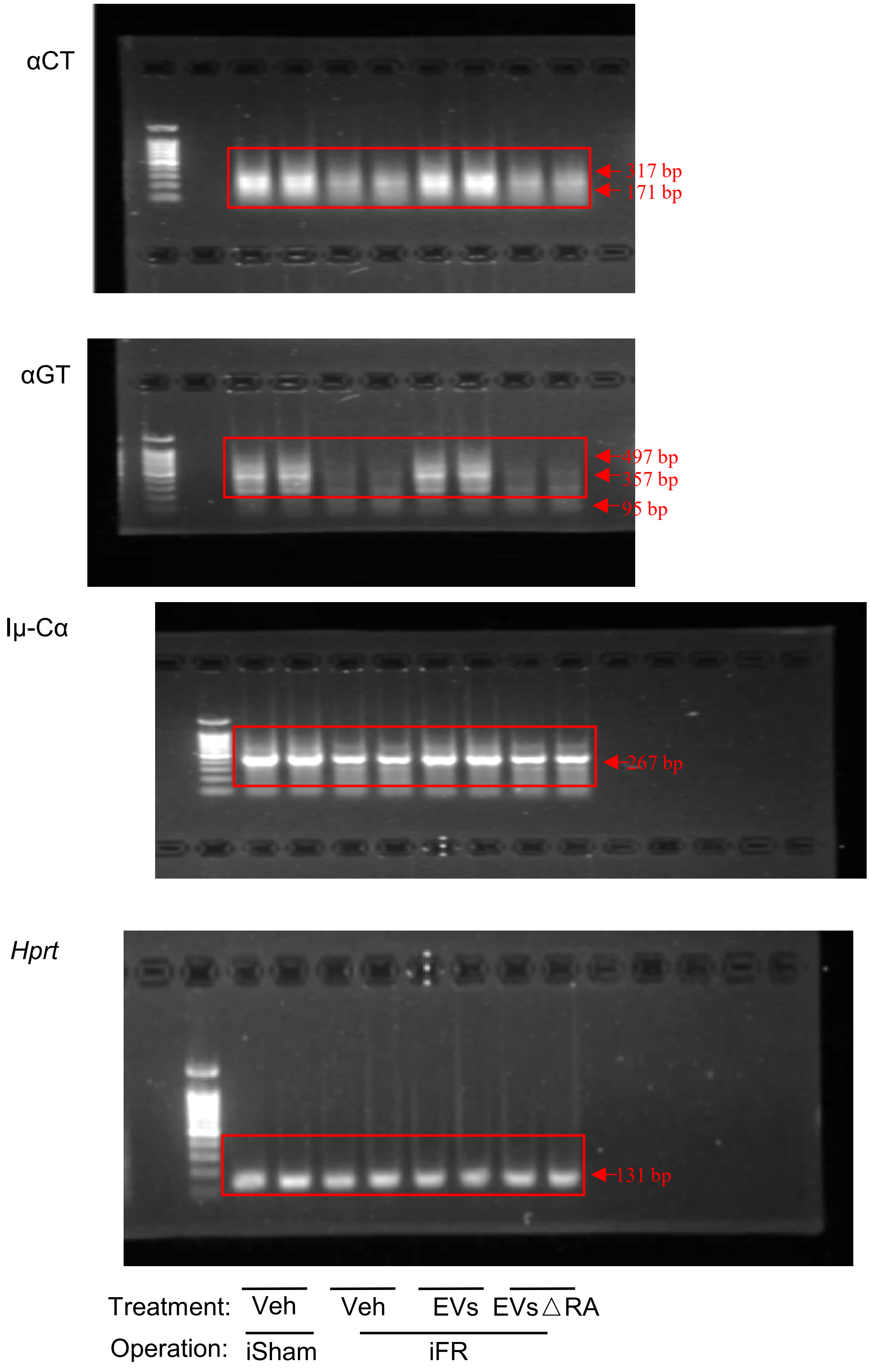

Supplementary Fig 20E

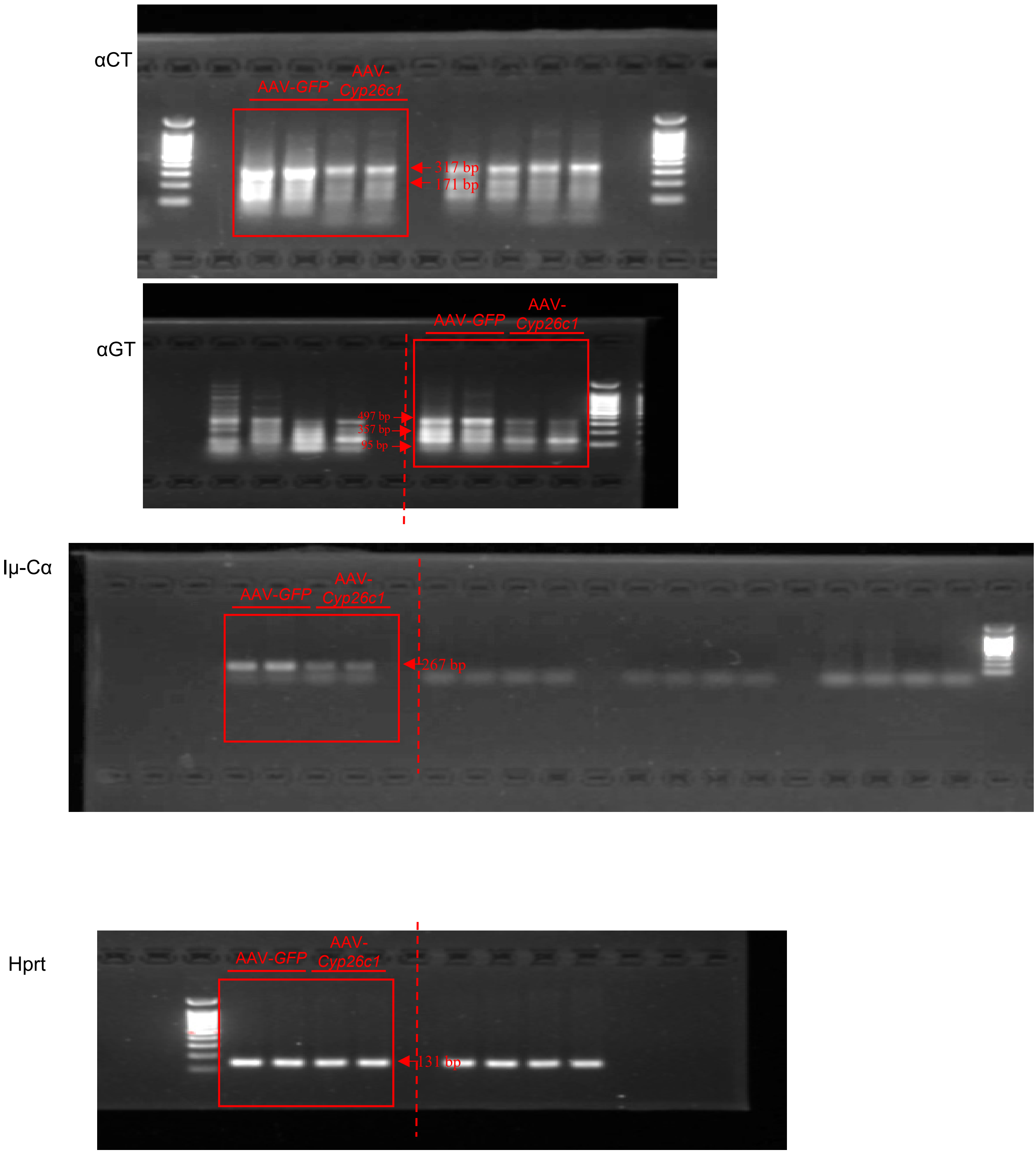

Supplementary Fig 24 D

CYP26C1

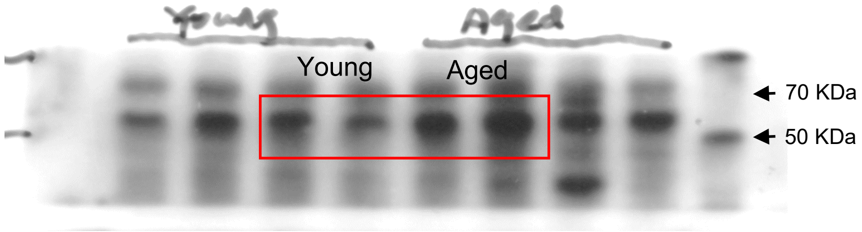

ALDH1A2

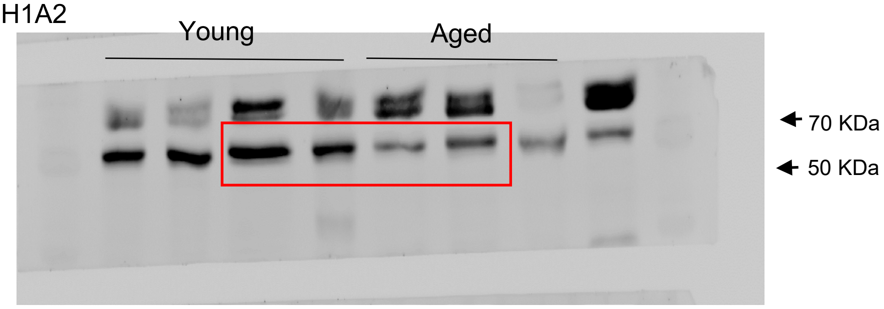

HSP90

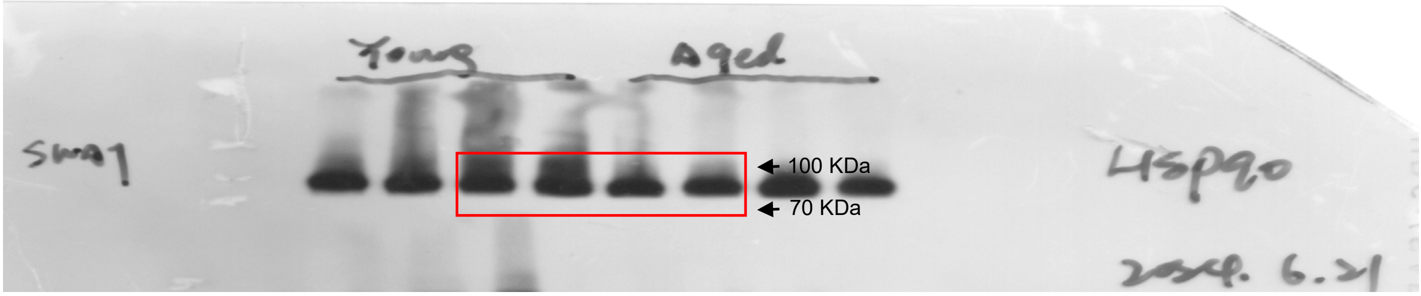

Supplementary Fig 25 C

CYP26C1

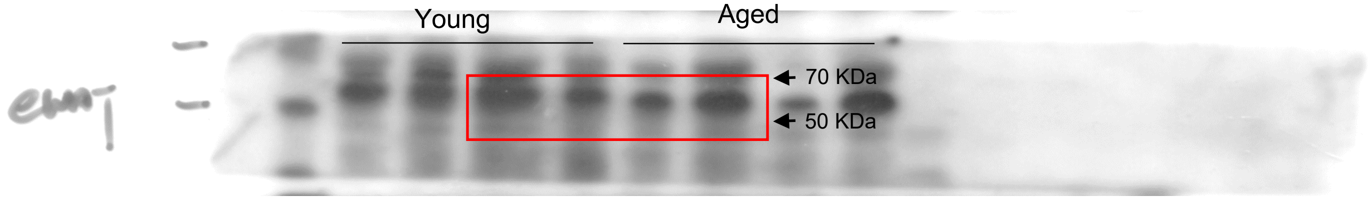

ALDH1A2

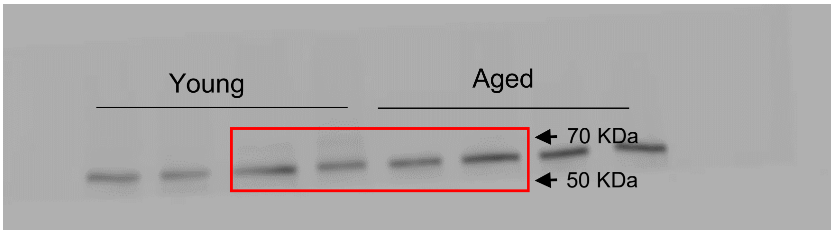

HSP90

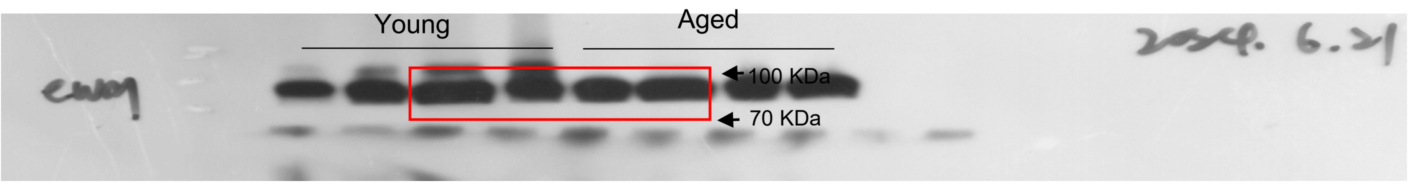

Supplementary Fig 25 D

CYP26C1

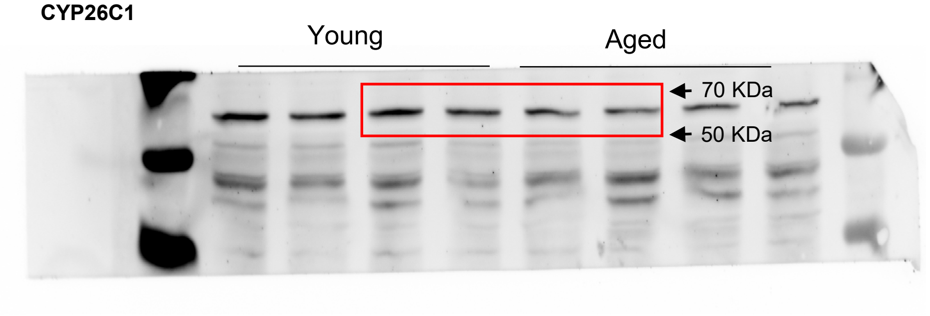

ALDH1A2

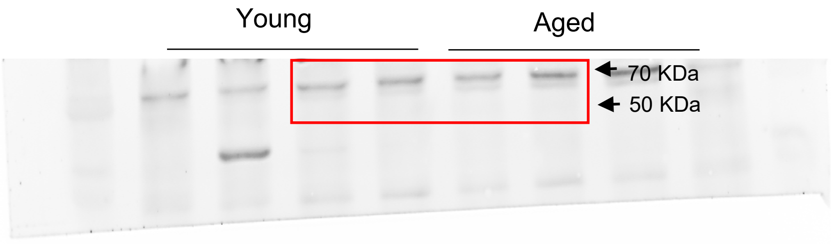

HSP90

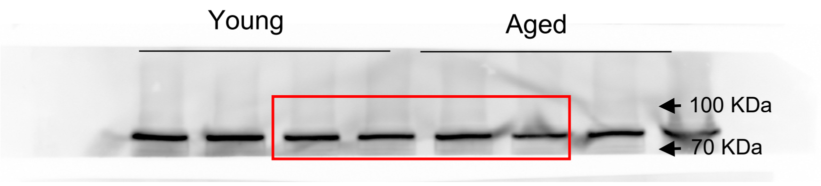

Supplementary Fig 26 C

LP:

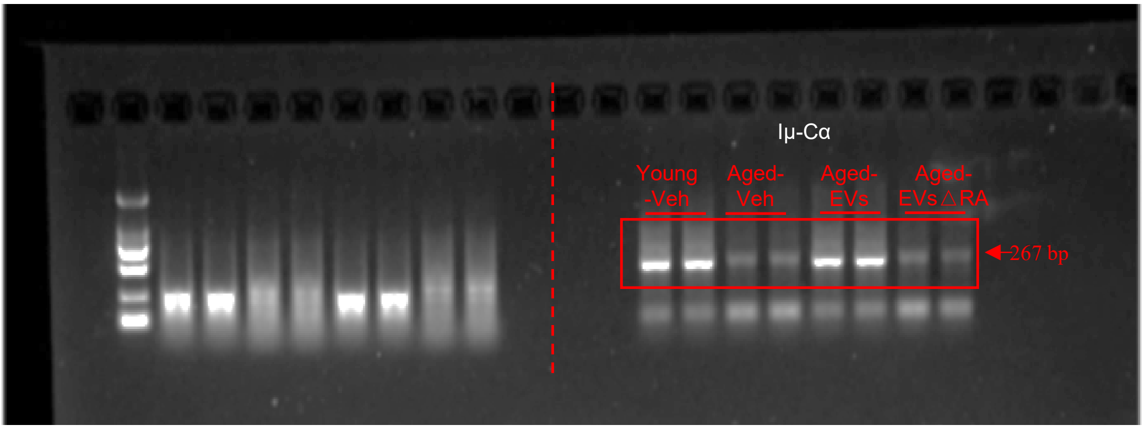

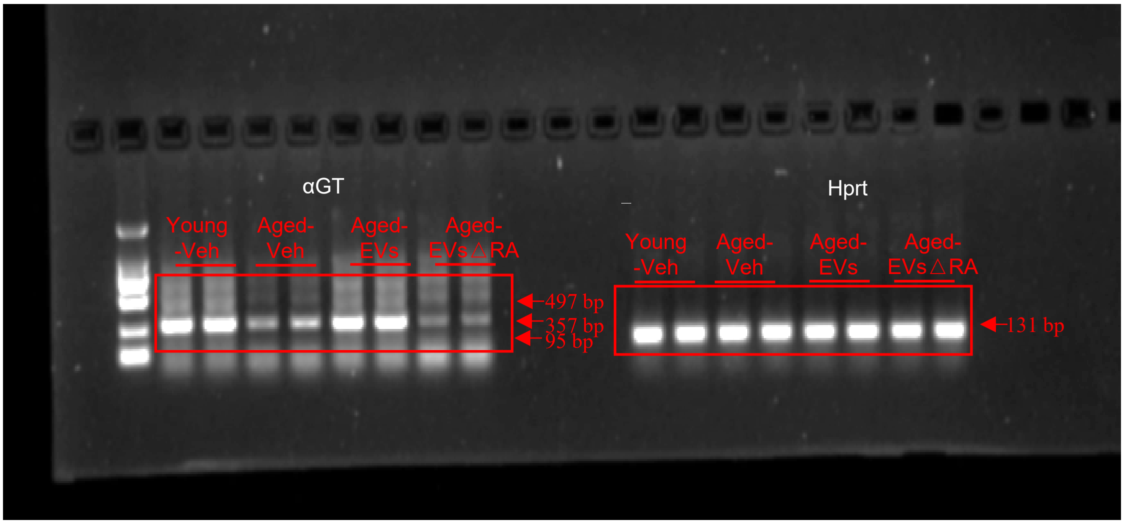

Supplement: Unedited blot and gel images [file jci-135-188947-s070.pdf]
